# Embedded Fe‐Cu Pairs Enable Tandem Nitrate‐to‐Ammonia Electroreduction

**DOI:** 10.1002/adma.202514840

**Published:** 2025-10-02

**Authors:** Yuxiao Liu, Xia Zhang, Solmaz Feizpoor, Hsiao‐Chien Chen, Linfeng Li, Yunpeng Zuo, Shengji Tian, Mengni Liu, Wenyu Hu, Muhammad Humayun, Kaifu Huo, Chade Lv, Yuanjie Pang, Dingsheng Wang, Xin Wang, Chundong Wang

**Affiliations:** ^1^ School of Integrated Circuits State Key Laboratory of New Textile Materials and Advanced Processing Huazhong University of Science and Technology Wuhan 430074 P. R. China; ^2^ Dual Master Program in Nano‐Electronic Engineering and Design Chang Gung University Taoyuan 33302 Taiwan; ^3^ Kidney Research Center Department of Nephrology Chang Gung Memorial Hospital Linkou Taoyuan 33305 Taiwan; ^4^ Department of Chemistry City University of Hong Kong Hong Kong 999077 P. R. China; ^5^ MIIT Key Laboratory of Critical Materials Technology for New Energy Conversion and Storage School of Chemistry and Chemical Engineering Harbin Institute of Technology Harbin 150001 P. R. China; ^6^ Department of Physics College of Science Shihezi University Xinjiang 832003 P. R. China; ^7^ Department of Physics Southern University of Science and Technology Shenzhen 518055 P. R. China; ^8^ Energy Water and Environment Lab College of Sciences and Humanities Prince Sultan University Riyadh 11586 Saudi Arabia; ^9^ School of Optical and Electronic Information Wuhan National Laboratory for Optoelectronics Huazhong University of Science and Technology Wuhan 430074 P. R. China; ^10^ Department of Chemistry Tsinghua University Beijing 100084 P. R. China

**Keywords:** active hydrogen, ammonia, bimetallic electrocatalyst, *e*‐NO_3_RR, three‐step relay mechanism

## Abstract

Electrochemical nitrate reduction (*e*‐NO_3_RR) to ammonia (NH_3_) represents a transformative technology that seamlessly integrates environmental remediation with resource regeneration. This approach is crucial for restoring equilibrium in the global nitrogen cycling, advancing green chemistry, and accelerating the transition toward a sustainable circular economy. However, under pH‐neutral conditions, the simultaneous occurrence of two competing reactions (Hydrogen Evolution Reaction and NO_3_RR) at the same active sites results in considerable interference, significantly limiting the catalytic efficiency and selectivity. Here a Fe‐Cu pair (Cu‐N_3_/Fe_3_‐N_8_) electrocatalyst is meticulously designed, achieving a NH_3_ production rate of 18.83 mg∙h^‒1^∙mg_cat_^‒1^ at −0.65 V versus the reversible hydrogen electrode (RHE), accompanied with a Faradaic efficiency of 97.1%. This as‐prepared Fe‐Cu pair overcomes the limitations of conventional bimetallic catalysts, which typically rely on direct atomic coupling. The electron‐deficient region formed by Cu–N_3_ enhances the adsorption of nitrate, while the electron‐rich domain generated by the Fe_3_–N_8_ cluster facilitates the adsorption of nitrite and promotes water activation. The spatially separated charge gradient optimizes the adsorption energies of multi‐step reaction intermediates, thereby establishing a relay mechanism. The work provides valuable insights into the design of multi‐active‐site electrocatalysts and offers a promising approach to addressing critical challenges in nitrogen resource conversion.

## Graphical abstract

A tandem Fe‐Cu pair catalyst, composing a single copper atom (Cu–N_3_) coordinated to adjacent iron clusters (Fe_3_N_8_), is designed. The electron‐deficient copper sites markedly enhance nitrate adsorption, while the electron‐rich iron clusters facilitate nitrite adsorption and water activation, thereby accelerating the electroreduction of nitrate and ultimately enabling efficient ammonia production.

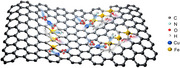

## 1 Introduction

Ammonia (NH_3_) is well‐known as a crucial component in modern agricultural and chemical industries. In recent years, it also attracts tremendous interest due to its potential as a carbon‐free energy carrier.^[^ ^1^, ^2^, ^3^ ^]^ Nonetheless, conventional industrial NH_3_ production predominantly relies on the Haber‐Bosch process, which generally operates under high temperature and high‐pressure conditions, accounting for ≈ 1%–2% of global energy consumption and 1.4% of annual CO_2_ emissions, thereby exacerbating both energy consumption and greenhouse gas effects.^[^ ^4^, ^5^, ^6^ ^]^ Consequently, the development of sustainable and energy‐efficient ammonia synthesis methods is of the utmost importance.^[^ ^7^ ^]^ On the other hand, anthropogenic activities, especially the ones in the current modern society continue to release reactive nitrogen into ecosystems, disrupting the balance of the global nitrogen cycle.^[^ ^8^, ^9^, ^10^ ^]^ Nitrate (NO_3_^−^) is a prominent nitrogen‐containing compound found in high quantities in industrial and agricultural effluents, posing substantial risks to ecological balance.^[^ ^11^, ^12^, ^13^, ^14^ ^]^ In this regard, the electrochemical nitrate reduction reaction (*e*‐NO_3_RR) stands out as a promising eco‐friendly approach for ammonia synthesis, which enables simultaneous nitrate removal and NH_3_ production using renewable electricity.^[^ ^15^, ^16^ ^]^

The development of highly active and selective electrocatalysts is indispensable for *e*‐NO_3_RR due to its nature of multi‐step proton and electron transfer processes, which leads to slow reaction kinetics and limited overall efficiency.^[^ ^17^, ^18^, ^19^, ^20^, ^21^ ^]^ Copper‐based electrocatalysts have sparked new interest in the *e*‐NO_3_RR applications due to their promising characteristics, such as natural abundance, versatile electrochemical activity, and tunable electronic structures with Lowest Unoccupied Molecular Orbital (LUMO) π^*^ orbitals that are complementary to nitrate.^[^ ^22^, ^23^, ^24^ ^]^ Recent studies have demonstrated the promise of copper‐based single‐atom catalysts (Cu SACs) for *e*‐NO_3_RR application.^[^ ^25^, ^26^, ^27^ ^]^ Nevertheless, the capacity of Cu SACs to overcome the linear scaling relationship between the catalytic surface adsorption strength and different reaction intermediates is restricted by their single active site in multi‐electron transfer processes.^[^ ^28^, ^29^, ^30^, ^31^, ^32^ ^]^ Impressively, catalysts with a Cu/Fe bimetallic site interact effectively with most of the nitrogen‐containing intermediates and show a high affinity for NO_3_^−^ adsorption.^[^ ^30^, ^33^, ^34^ ^]^ Further studies indicate that multi‐core Fe sites (e.g., iron clusters) exhibit more favorable states for oxygen intermediates bonding than isolated Fe sites.^[^ ^35^, ^36^ ^]^ Consequently, developing Cu/Fe hybrid catalysts with precisely controlled atomic configurations for enhanced *e*‐NO_3_RR activity is highly desirable yet challenging.

In this study, we designed a novel tandem electrocatalyst (designated as CuFe_x_‐NC), consisting of a copper single atom (Cu‐N_3_) coupled with an adjacent iron cluster (Fe_3_‐N_8_) decorated on nitrogen‐doped carbon. The CuFe_x_‐NC catalyst exhibits a Faradaic efficiency (FE) of 97.1% and an NH_3_ yield rate of 18.83 mg∙h^‒1^∙mg_cat_^‒1^ under neutral conditions at −0.65 V versus the reversible hydrogen electrode (RHE) for *e*‐NO_3_RR. Comprehensive mechanistic investigations, including Kinetic isotope effects (KIEs), electron paramagnetic resonance (EPR) spectroscopy, differential electrochemical mass spectrometry (DEMS), in situ Fourier transform infrared (FTIR) spectroscopy, and density functional theory (DFT) calculations, were employed to clarify the synergistic effect of bimetallic sites, which underlies the exceptional *e*‐NO_3_RR performance. Our newly constructed Cu‐N_3_/Fe_3_‐N_8_ structure facilitates electron redistribution within the trinuclear Fe cores while preserving Fe‐N_4_ activity. This configuration creates a spatial charge polarization gradient, characterized by electron‐deficient Cu sites and electron‐rich Fe clusters, which promotes abundant H^*^ generation at Fe sites and efficient NO_3_^−^ adsorption and activation at Cu sites. All these effects synergistically enable highly efficient electrochemical nitrate reduction to ammonia.

## 2 Results and Discussion

### 2.1 Preparation and Characterization of Electrocatalyst

**Figure 1:**
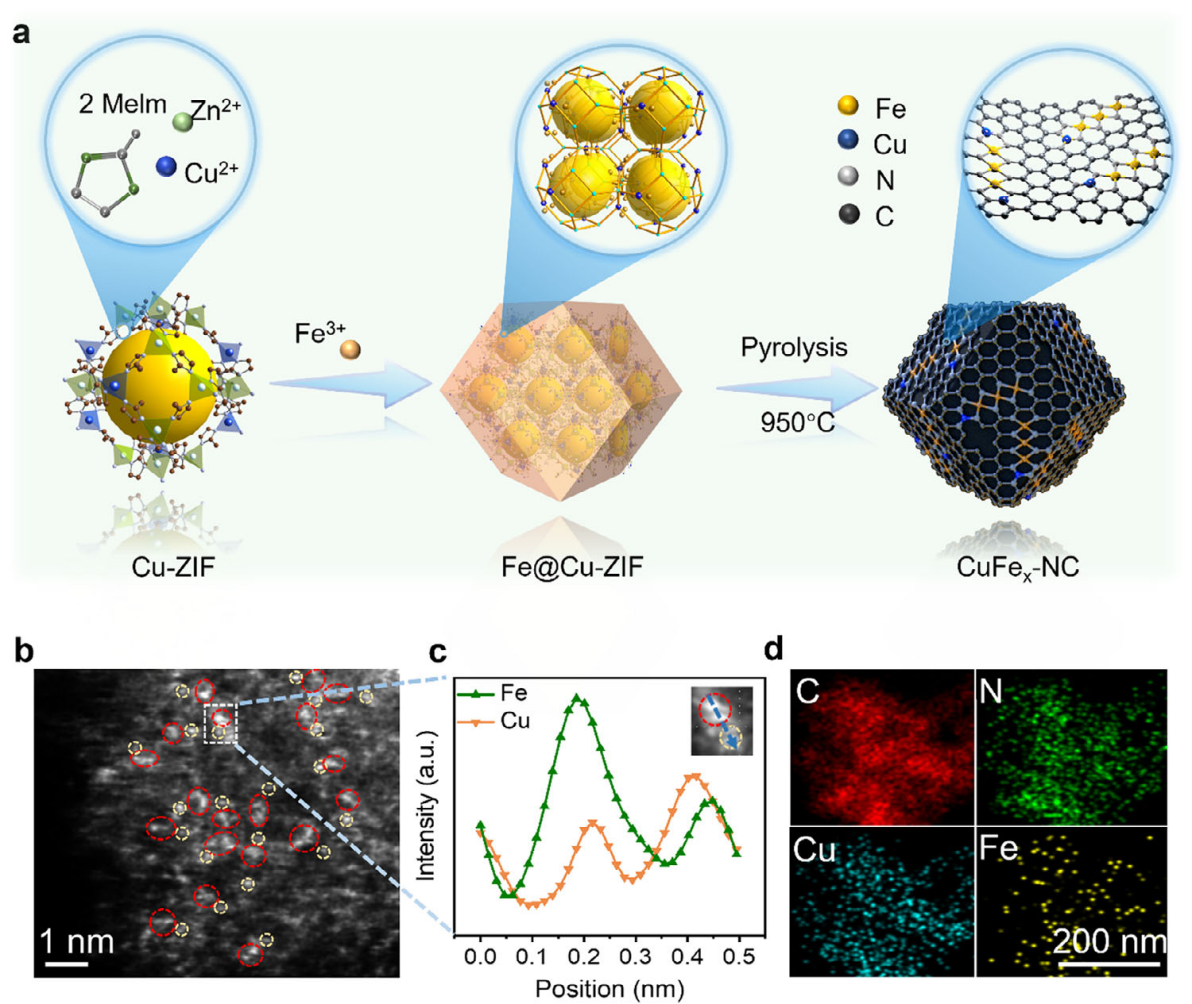
Preparation and structure characterization of CuFe_x_‐NC. a) Schematic diagram for the synthesis of CuFe_x_‐NC catalyst. b) HAADF‐STEM image of CuFe_x_‐NC (The red circle represents a cluster, while the yellow circle denotes a single atom.). c) EDX linear scanning signal intensity spectra of CuFe_x_‐NC. d) EDX mapping images of CuFe_x_‐NC.

The step‐wise synthesis route of the CuFe_x_‐NC catalyst is illustrated in **Figure** **1**a. First, a Cu‐doped porous zeolite imidazolate framework (Cu/ZIF) was prepared (Figure S1a, Supporting Information). Subsequently, Fe^3+^ solution was introduced into a homogeneously dispersed Cu/ZIF n‐propanol solution, by which the Fe^3+^ containing precursor (Fe@Cu/ZIF) was formed (Figure S1b, Supporting Information). A pyrolysis treatment at 950 °C was further carried out, yielding the final product of nitrogen‐doped carbon (NC) framework with coordinated Cu and Fe atoms (Figure S1c, Supporting Information). For comparison, control samples of Cu‐NC and Fe‐NC were also synthesized using analogous procedures. The X‐ray diffraction (XRD) analysis of the phase structures of all samples revealed broad graphitic peaks, indicating that no oxide or metallic nanocrystals were present (Figure S2a, Supporting Information).^[^ ^37^ ^]^ The two distinct bands identified in the Raman spectra are the D‐band (1345 cm^−1^) for disordered carbon and the G‐band (1582 cm^−1^) for graphitic carbon, as shown in Figure S2b (Supporting Information). CuFe_x_‐NC reveals a higher D to G band intensity ratio (I_D_/I_G_) than Cu‐NC and Fe‐NC, indicating an enhanced defect density.^[^ ^38^ ^]^ Also, it is noteworthy that CuFe_x_‐NC possesses a higher specific surface area (1301.2 m^2^∙g^−1^) compared to Cu‐NC (1103.1 m^2^∙g^−1^) and Fe‐NC (963.3 m^2^∙g^−1^) (Figure S3, Supporting Information).

No nanoparticles are discerned in the as‐prepared CuFe_x_‐NC catalyst as evidenced by the transmission electron microscopy (TEM) images (Figure S4, Supporting Information). Concurrently, uniformly distributed bright spots on the substrate surface were observed using high‐angle annular dark‐field scanning electron microscopy (HAADF‐STEM) imaging, further confirming the absence of metal nanoparticles (Figure 1b). Moreover, the Energy Dispersive X‐Ray Spectroscopy (EDX) intensity line scanning analysis across selected regions, transitioning from cluster regions (red‐circled) to monatomic regions (yellow‐circled), demonstrated sequential signals for Fe and Cu (Figure 1c), evidencing the presence of single atoms in proximity to clusters. The EDX mapping of Cu, Fe, C, and N elements further demonstrating their homogeneous distribution over the carbon matrix (Figure 1d).

**Figure 2:**
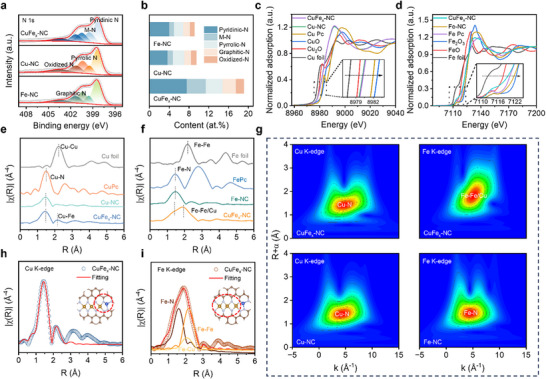
Structural analysis of CuFe_x_‐NC. a) N 1s XPS spectra of CuFe_x_‐NC, Cu‐NC, and Fe‐NC. b) Different N contents in CuFe_x_‐NC, Cu‐NC, and Fe‐NC. XANES spectra of c) Cu K‐edge and d) Fe K‐edge. Fourier‐transform EXAFS curves of e) Cu K‐edge and f) Fe K‐edge. g) WT‐EXAFS spectra of CuFe_x_‐NC, Cu‐NC, and Fe‐NC. EXAFS fitting for CuFe_x_‐NC in R‐space at the h) Cu K‐edge and i) Fe K‐edge. (Inset: model of Cu‐N_3_/Fe_3_‐N_8_. Fe, yellow; Cu, blue; N, gray; C, brown spheres).

To understand the chemical states of the as‐prepared CuFe_x_‐NC, X‐ray photoelectron spectroscopy (XPS) was employed.^[^ ^39^ ^]^ Fe, Cu, N, and C elements were detected in the XPS survey spectrum of CuFe_x_‐NC (Figure S5a, Supporting Information), supporting the findings from EDS mapping (Figure 1d). As displayed in **Figure** **2**a, the N 1s spectra were deconvoluted into five distinct peaks at 398.4, 399.5, 400.2, 401.2, and 403.3 eV, which represent pyridinic nitrogen, metal coordinated nitrogen (M‐N), pyrrolic nitrogen, graphitic nitrogen, and oxidized nitrogen,^[^ ^40^ ^]^ respectively. According to the Cu 2p spectra (Figure S5c, Supporting Information), both CuFe_x_‐NC and Cu‐NC mainly display the Cu(I) oxidation state. By comparison, it was noticed that CuFe_x_‐NC shows a higher binding energy shift, suggesting an enhanced oxidation state of Cu in CuFe_x_‐NC compared to that in Cu‐NC. Fe 2p spectra were also collected as shown in Figure S5d (Supporting Information). Since a lower binding energy shift is discerned in the Fe 2p spectra, it informs that Fe valence states in CuFe_x_‐NC are reduced relative to that in Fe‐NC. Additionally, it can be confirmed that Fe‐Fe clusters did not form due to the absence of signals indicative of zero‐valent iron. It is noteworthy that the formation of Fe‐N moieties in CuFe_x_‐NC is accompanied by a significant increase in the M─N bond content due to the incorporation of iron atoms into Cu‐NC (Figure 2b; and Table S1, Supporting Information). These findings suggest that it is a Fe‐N configuration with Fe clusters involved, rather than an Fe‐Fe arrangement.

To gain an in‐depth understanding of the chemical environment and coordination configurations of Cu and Fe sites in CuFe_x_‐NC, Cu‐NC, and Fe‐NC, X‐ray absorption near‐edge spectroscopy (XANES) and extended X‐ray absorption fine spectroscopy (EXAFS) spectra were collected.^[^ ^41^ ^]^ The Cu K‐edge XANES spectrum (Figure 2c) reveals that the absorption profiles for CuFe_x_‐NC and Cu‐NC are intermediate between the references Cu_2_O and CuO, with a closer alignment to Cu_2_O, suggesting a predominance of Cu(I) oxidation states.^[^ ^42^ ^]^ More specifically, the slight positive shift detected in CuFe_x_‐NC compared to Cu‐NC illustrates an increased copper oxidation state (the inset of Figure 2c). On the other hand, the Fe K‐edge XANES spectrum (Figure 2d) demonstrates that the valence state of Fe in both CuFe_x_‐NC and Fe‐NC lies between Fe (II) and Fe (III), as proven by the absorption spectra of CuFe_x_‐NC and Fe‐NC situated between FeO and Fe_2_O_3_.^[^ ^43^ ^]^ Additionally, evidence of electron transfer between Cu and Fe centers is shown by the slight negative shift in CuFe_x_‐NC relative to Fe‐NC, indicating reduced iron valence states (the inset of Figure 2d). The presence of a characteristic pre‐edge peak at ≈ 7113 eV in the Fe K‐edge spectrum confirms that CuFe_x_‐NC contains square‐planar or centrosymmetric Fe‐N_4_ structures.^[^ ^44^ ^]^ The higher pre‐edge peak intensity of CuFe_x_‐NC compared to those of Fe‐NC and FePc indicates that Cu reduces the Fe‐N_4_ symmetry.^[^ ^45^, ^46^ ^]^

Fourier transform EXAFS (FT‐EXAFS) of Cu K‐edge for CuFe_x_‐NC reveals a primary peak at ≈ 1.5 Å corresponding to Cu‐N scattering, being consistent with the cases of Cu‐NC and CuPc (Figure 2e). The absence of a distinct second shell peak at 2.1 Å in Cu‐NC represents Cu‐Fe coupling after Fe incorporation.^[^ ^30^ ^]^ The absence of metallic copper particles was verified since the spectral profiles of CuFe_x_‐NC and Cu‐NC varied considerably from Cu foil. The FT‐EXAFS of Fe K‐edge of CuFe_x_‐NC (Figure 2f) displays a prominent peak between 1.4 and 2.1 Å, which corresponds to a Fe‐N coordination at 1.45 Å and a Fe‐Fe path at 2 Å. Based on the differences in peak intensity between the Fe and Cu K‐edges, the 2 Å feature was attributed to Fe‐Fe coordination rather than Fe‐Cu scattering contributions.^[^ ^35^ ^]^ It can be deduced that the presence of copper alters the Fe‐Fe distance compared to that in Fe foil (2.3 Å), confirming the presence of Cu‐Fe coupling in CuFe_x_‐NC.^[^ ^47^ ^]^ Wavelet transform EXAFS (WT‐EXAFS) were collected as well, which provides additional structural insights. As shown in Figure 2g, the Cu K‐edge exhibits maximum intensities for CuFe_x_‐NC and Cu‐NC at ≈ 4.9 and 4.5 Å, respectively, which correspond to the Cu‐N scattering paths.^[^ ^48^ ^]^ Similarly, the WT contour of the Fe K‐edge indicates that the maximum intensity of the CuFe_x_‐NC is at ≈ 5.1 Å, which is a shift from the maximum intensity of 4.5 Å (associated with the Fe‐N bond scattering path) for Fe‐NC. This observation spans both coordination shells and consistent with Fe‐N cluster formation. Furthermore, the distinct WT contour pattern, in comparison to the Fe foil, confirms the presence of isolated Fe clusters rather than crystalline iron structures (Figure S6, Supporting Information).^[^ ^49^ ^]^

EXAFS fitting was further conducted to clarify the local coordination environments of Cu and Fe in the synthesized catalysts. The fitting of Cu‐NC and Fe‐NC reveal M‐N shells with coordination numbers of 4, confirming the formation of M‐N_4_ moieties (Figure S7 and Table S2, Supporting Information). The fitting for CuFe_x_‐NC reveals a Cu‐N shell with a coordination number of 3, indicating the formation of a Cu‐N_3_ moieties (Figure 2h; Table S3, Supporting Information). Analysis of the Fe‐Fe bond intensity in FT‐EXAFS of CuFe_x_‐NC indicates the presence of Fe trimers rather than Fe‐N dimers.^[^ ^35^ ^]^ Structural modeling was subsequently performed using fixed Cu‐N_3_ and Fe‐N trimer configurations (Figure S8, Supporting Information). According to Fe K‐edge EXAFS spectral simulation for the four different Fe_3_N_x_ samples, Fe_3_‐N_8_‐1 is the most probable configuration for the Fe cluster, with the best agreement between the Fe_3_‐N_8_‐1 fitting model and experimental results (Figure S9 and Table S4, Supporting Information). Density functional theory (DFT) calculations were employed to evaluate two possible CuFe_x_‐NC configurations (Figure S10, Supporting Information). The Cu‐N_3_/Fe_3_‐N_8_‐1 model is in good agreement with the collected experimental R‐space data as well (Figure S11a and Table S4, Supporting Information). In particular, the k‐space oscillations closely match the experimental spectra (Figure S11b, Supporting Information). The validity of the proposed Cu‐N_3_/Fe_3_‐N_8_‐1 configuration is further supported by consistent fitting results for the Cu K‐edge across all three models (Figure S12 and Table S3, Supporting Information).

EXAFS simulation was employed to elucidate the Cu‐Fe bonding structure of the synthesized catalyst.^[^ ^50^ ^]^ Among the five predicted models (termed as Cu‐N_3_/Fe_3_‐N_8_‐1, Cu‐N_3_/Fe_3_‐N_8_‐2, Cu‐N_3_/FeN_x_@Fe_x_‐1, Cu‐N_3_/FeN_x_@Fe_x_‐2, Cu‐N_3_/Fe_3_‐N_8_‐3), the Cu K‐edge spectrum of Cu‐N_3_/Fe_3_‐N_8_‐1 most accurately reproduces the experimental spectral features, with simulations of the Cu K‐edge and Fe K‐edge XANES and EXAFS closely matching the experimental results. A more detailed comparison of the simulated EXAFS for the five predicted Cu/Fe models can be found in Figure S13 (Supporting Information). As such, the Cu‐N_3_/Fe_3_‐N_8_‐1 is recognized as the optimal configuration for CuFe_x_‐NC. As expected, the Cu and Fe K‐edge EXAFS fitting of the Cu‐N_3_/Fe_3_‐N_8_‐1 configuration are in line with the experimental results (Figure 2h,i). Additionally, the K‐space oscillations of Cu/Fe K‐edge fitting curves closely matched the experimental spectra (Figure S14, Supporting Information), further supporting the validity of our proposed Cu‐N_3_ /Fe_3_‐N_8_‐1 configuration.

### 2.2 Electrocatalytic NO_3_RR Performance

**Figure 3:**
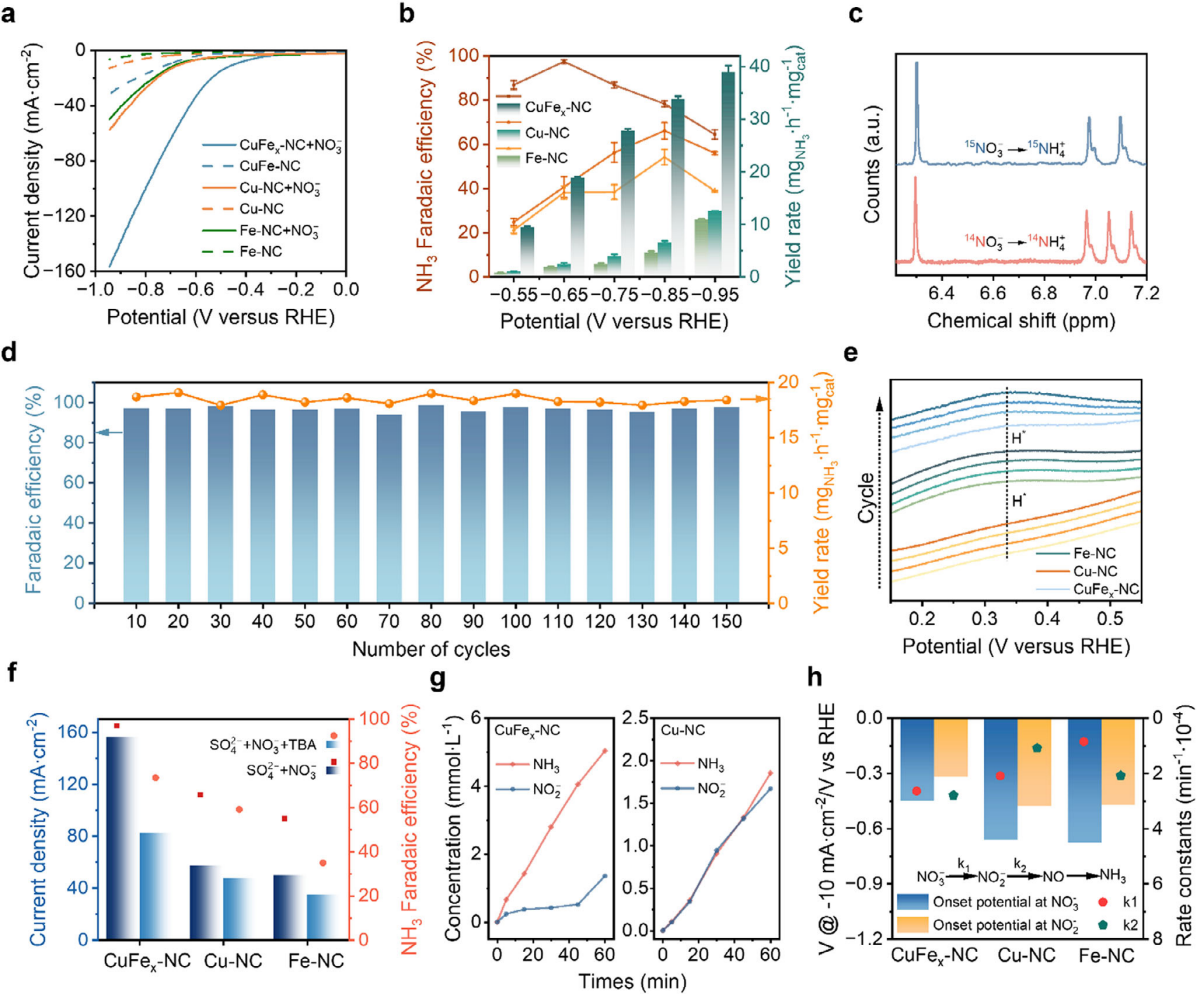
Electrochemical NO_3_RR performance of the catalysts. a) LSV curves in solution with or without nitrates. b) Diagram of Faraday efficiency and yield rate of CuFe_x_‐NC, Cu‐NC, and Fe‐NC. c) ^1^H NMR spectra of ^15^NH_3_ and ^14^NH_3_ when ^15^NO_3_^−^ and ^14^NO_3_^−^ were used as the N sources, respectively. d) Cycling performance at −0.65 V (versus RHE) of CuFe_x_‐NC in 0.5 M Na_2_SO_4_ and 0.1 m NaNO_3_ alkaline solution. e) CV curves of CuFe_x_‐NC, Cu‐NC, and Fe‐NC. f) H∙ free radical quenching experiment. g) The product concentration of CuFe_x_‐NC and Cu‐NC products with respect to electrolysis time. h) LSV‐derived onset potentials at 10 mA∙cm^−2^ and the corresponding calculated reaction constants of CuFe_x_‐NC, Cu‐NC, and Fe‐NC for NO_3_^−^ and NO_2_^−^ reduction.

*E*‐NO_3_RR evaluation was conducted using a three‐electrode system in an H‐type electrolytic cell containing 0.5 m Na_2_SO_4_ and 0.1 M NaNO_3_ as the electrolyte. **Figure** **3**a depicts the linear sweep voltammetry (LSV) curves of the samples measured in Na_2_SO_4_ electrolyte with and without NO_3_^−^. The significant increase in the current density of the catalysts in the presence of NO_3_^−^ clearly reveals the occurrence of electrolytic NO_3_^−^ reduction on these catalysts. Furthermore, CuFe_x_‐NC requires a lower driving potential compared to Cu‐NC and Fe‐NC at the same current density, indicating the enhanced *e*‐NO_3_RR performance. Quantification of the reduction products following chronoamperometric testing was conducted using UV–vis spectrophotometry and Gas chromatography. Calibration curves for this method are presented in Figures S15–S18 (Supporting Information). CuFe_x_‐NC exhibits optimal Faraday efficiency (FE) for NH_3_ and the highest NH_3_ yield rates at ‐0.65 V versus RHE (Figure 3b), i.e., 97.1% FE and 18.83 mg∙h^‒1^∙mg_cat_^‒1^ NH_3_ yield. The distribution and quantification of additional reaction products are represented in Figure S19 and Table S5 (Supporting Information). The accelerated reaction kinetics at the bimetallic active sites are concluded from the positive shift in the FE peak potential for CuFe_x_‐NC (−0.65 V versus RHE) compared to the Cu‐NC and Fe‐NC (−0.85 V vs RHE). Isotope labeling experiments utilizing ^14^NO_3_^−^ and ^15^NO_3_^−^ solutions were performed to elucidate the reaction mechanism. Using ^1^H NMR, it determines that NH_3_ is produced from the reduction of NO_3_^−^, with no contribution from interfering nitrogen species. (Figure 3c). In addition, the formation of NH_4_^+^ product formation is also verified through ^1^H NMR spectroscopy (Figure S20, Supporting Information), with quantitative results corroborating colorimetric measurements (Figure S21, Supporting Information). The long‐term operational stability of CuFe_x_‐NC was verified through durability testing in 0.5 m Na_2_SO_4_ and 0.1 M NaNO_3_. The results show that the catalyst maintains its performance over 150 cycles (1 h per cycle), with a yield rate of 18.47 mg∙h^‒1^∙mg_cat_^‒1^ and ≈ 96.8% FE (Figure 3d; Figure S22, Supporting Information). Noteworthy, the properties of the catalyst remained stable after the reaction (Figures S23–S25 and Table S6, Supporting Information). The performance comparison presented in Table S7 (Supporting Information) highlights the superior catalytic activity of CuFe_x_‐NC in the *e*‐NO_3_RR, outperforming most of the previously reported catalysts. This enhanced performance underscores its potential as a highly efficient and practical candidate for sustainable ammonia production.

Systematic electrochemical measurements were conducted to demonstrate the advanced nature of the bimetallic sites and to clarify their specific roles. In cyclic voltammetry (CV) curves of CuFe_x_‐NC, Cu‐NC, and Fe‐NC, H^*^ peaks at ≈ 0.3 V versus RHE were discerned, for which the peak intensities follow the orders of CuFe_x_‐NC > Fe‐NC > Cu‐NC (Figure 3e; and Figure S26, Supporting Information). This observation suggests that H^*^ was generated during e‐NO_3_RR, with Fe sites displaying a significant capacity for H^*^ generation compared to Cu sites. A hydrogen radical (H•) quencher experiment was conducted employing tert‐butanol (TBA) as an indicator to further verify the participation of H^*^ in *e*‐NO_3_RR. The significant decrease in current density and FE (NH_3_) after TBA addition emphasizes the critical role of H^*^ involvement in *e*‐NO_3_RR and its necessity for achieving the optimal reaction rate (Figure 3f; Figure S27, Supporting Information). Within the potential window of −0.4 to −1 V versus RHE, Cu‐NC demonstrates higher NO_3_^−^‐to‐NH_3_ conversion efficiency and superior current density compared to Fe‐NC (Figure S28a, Supporting Information), indicating preferential production of NO_2_^−^ at Cu sites. What's more, LSV curves measured in NO_2_^−^‐containing solutions reveal higher current densities for Fe‐NC than Cu‐NC (Figure S28b, Supporting Information). Cu‐NC shows approximately equal yields (50%) of NH_3_ and NO_2_^−^ (Figure 3g) at maximum FE potential, signifying synchronized concentration evolution at Cu sites. In contrast, CuFe_x_‐NC exhibits a linear increase in NH_3_ concentration over time with Fe assistance, while NO_2_^−^ concentrations remain relatively low and plateau rapidly. The nonlinear increase in NO_2_^−^ concentration indicates minimal accumulation of NO_2_^−^ in the electrolyte, enabling efficient diffusion from Cu to Fe sites for subsequent reduction to NH_3_ through H^*^ coupling. This observation highlights the synergistic interactions between Cu and Fe sites, where H^*^ and NO_2_^−^ species generated at Fe and Cu sites, respectively, exhibit mutual migration capabilities, thereby accelerating overall reaction kinetics.

To unveil the transport and diffusion mechanisms of H^*^, NO_3_^−^, and NO_2_^−^ between Cu and Fe sites, as well as understanding the reaction kinetics, a comparative analysis of the reduction onset potentials for NO_3_^−^ and NO_2_^−^ at a current density of 10 mA∙cm^−2^ was carried out. The results show that CuFe_x_‐NC exhibits positive potential shifts of 228  and 213 mV relative to Cu‐NC and Fe‐NC, respectively, during NO_3_^−^ reduction (Figure 3h; Table S8, Supporting Information). Although the conversion of NO_3_^−^ to NO_2_^−^ is the primary driver of this enhancement, the simultaneous catalysis of NO_3_^−^ to NH_3_ at Cu and Fe sites also contributes to the increased overpotential. CuFe_x_‐NC exhibits comparable reduction potentials for both NO_3_^−^ and NO_2_^−^ species, in contrast to the behavior observed in Cu‐NC and Fe‐NC (Figure S28, Supporting Information). In accordance with the temporal evolution of NO_2_^−^ and NH_3_ concentrations (Figure 3g), this observation demonstrates effective diffusion of NO_2_^−^ from Cu to Fe sites for subsequent reduction to NH_3_. The first‐order reaction rate constant (k_1_) for converting NO_3_^−^ to NO_2_^−^ and the second‐order reaction rate constant (k_2_) for transforming NO_2_^−^ to NH_3_ were determined by kinetic analysis (Figure S29 and Table S8, Supporting Information). The preferential reduction of NO_3_^−^ to NO_2_^−^ at Cu sites, followed by NO_2_^−^ desorption, is demonstrated by the observation that k_1_ for Cu‐NC was twice as large as k_2_ (Figure 3h). In contrast, the k_1_ value for Fe‐NC is approximately half of k_2_, indicating a rapid conversion of NO_2_^−^ to NH_3_ at Fe sites. These findings provide further evidence that CuFe_x_‐NC undergoes a bimetallic relay catalysis mechanism.^[^ ^51^ ^]^ Furthermore, electrochemical impedance spectroscopy (EIS) measurements revealed that the charge transfer resistance (R_ct_) of CuFe_x_‐NC in nitrate‐containing electrolyte was significantly lower than that of Cu‐NC and Fe‐NC, and substantially smaller than that measured in nitrate‐free electrolyte (Figure S30, Supporting Information). These results demonstrate that the Cu–Fe dual sites synergistically enhance nitrate ion adsorption and facilitate interfacial charge transfer.

Additionally, assessments of the electrochemical active surface area (ECSA) indicate that CuFe_x_‐NC exhibits a larger active area compared to Cu‐NC and Fe‐NC (Figure S31, Supporting Information). Moreover, the ECSA‐normalized current density further confirmed the superior intrinsic catalytic activity of CuFe_x_‐NC (Figure S32, Supporting Information).

### 2.3 Catalytic Mechanism

**Figure 4:**
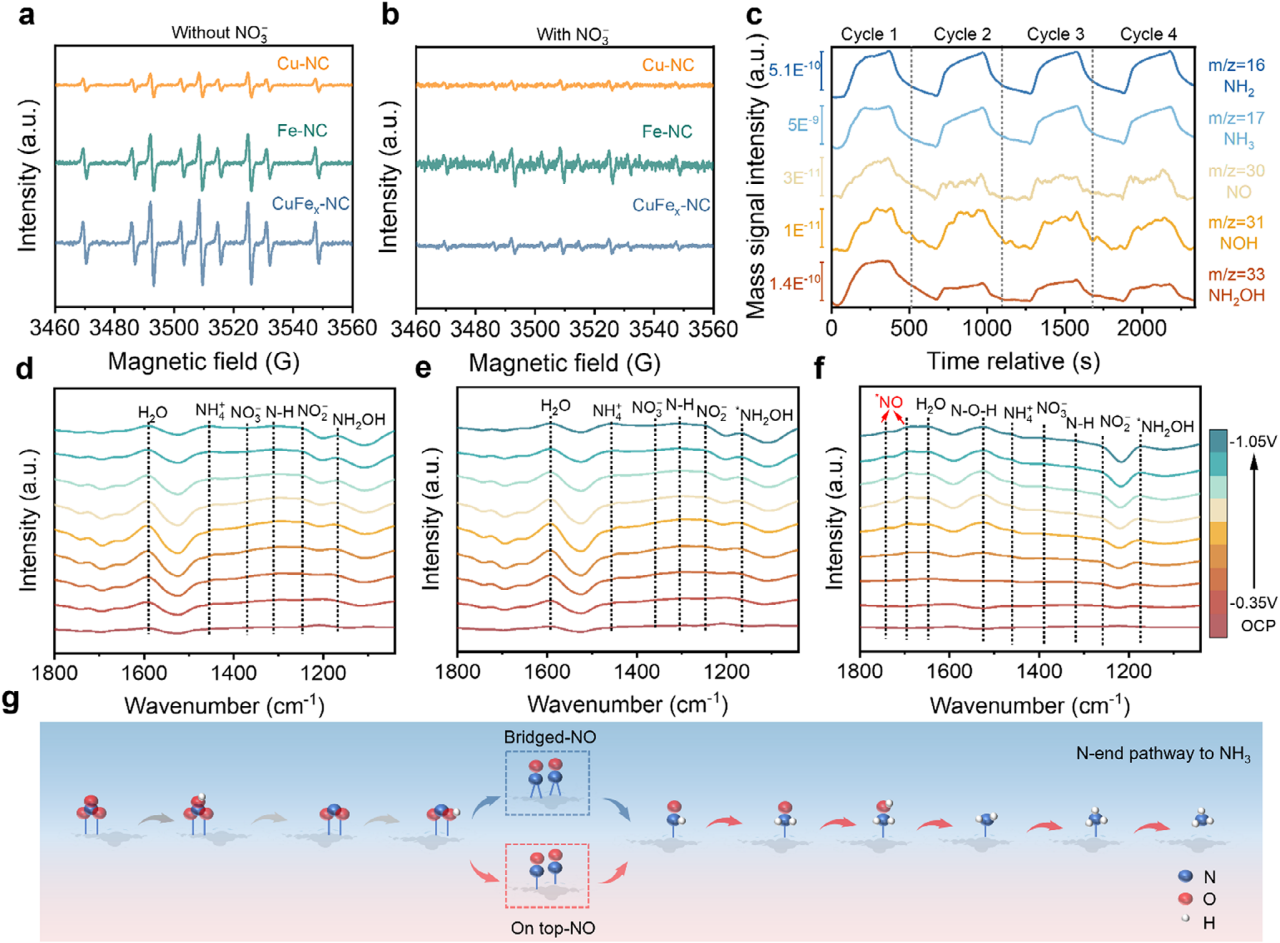
Reaction path analysis of NO_3_RR. EPR spectra of CuFe_x_‐NC, Cu‐NC, and Fe‐NC after 5 min of electrocatalysis in a solution of Na_2_SO_4_ a) without NO_3_^−^ and b) with NO_3_^−^. c) DEMS of CuFe_x_‐NC. In situ FTIR spectra of d) Cu‐NC, e) Fe‐NC, and f) CuFe_x_‐NC. g) Schematic illustration for the deduced *e*‐NO_3_RR reaction pathway over CuFe_x_‐NC.

In situ measurements were used to further elucidate the reaction mechanism. Electron paramagnetic resonance (EPR) spectroscopy was employed to investigate H^*^ spillover processes with 5,5‐dimethyl‐1‐pyrroline N‐oxide (DMPO) as a trapping agent.^[^ ^52^ ^]^ The formation of H• was confirmed by the EPR spectra of CuFe_x_‐NC, Fe‐NC, and Cu‐NC under nitrate‐free conditions, which present nine distinct signals with an intensity ratio of 1:1:1:2:1:2:1:1 (**Figure** **4**a). The significantly stronger EPR signal intensity of Fe‐NC compared to Cu‐NC indicates preferential H^*^ adsorption/desorption at Fe sites. Cu‐NC shows negligible DMPO‐H signals in the presence of nitrate (Figure 4b), suggesting that Cu sites undergo rapid H^*^ consumption via coupling with NO_3_^−^. In contrast, Fe‐NC demonstrates disproportionate rates of H^*^ generation and consumption by maintaining comparable DMPO‐H signals regardless of the presence of nitrate. These EPR results are consistent with H• quenching experiments, which reveal that the spillover of H^*^ from Fe to Cu sites facilitates NH_3_ generation through NO_3_^−^ reduction. Also, it is evidenced by the weaker signals observed in CuFe_x_‐NC compared to the Fe‐NC (Figure 3f).

In order to identify the intermediates in *e*‐NO_3_RR, differential electrochemical mass spectrometry (DEMS) measurement was carried out for four cycles, monitoring the m/z signals for hydroxylamine (NH_2_OH, 33), NOH (31), NO (30), NH_3_ (17), and NH_2_ (16) (Figure 4c). Although Cu‐NC and Fe‐NC exhibit similar characteristic signals (Figure S33, Supporting Information), they are distinguished from CuFe_x_‐NC by representing distinct NO_2_ (m/z 46) and H_2_ (m/z 2) signals, indicating significant byproduct formation. Comparative analysis demonstrated a lower NO_2_ signal intensity for Fe‐NC compared to Cu‐NC, thereby confirming the enhanced NO_2_ reactivity at Fe sites. Additionally, the prominent H_2_ signals from Fe‐NC indicate substantial H^*^ generation at Fe sites with incomplete utilization in *e*‐NO_3_RR, being consistent with EPR observations (Figure 4b).

The reaction intermediates in *e*‐NO_3_RR were further characterized using in situ FTIR spectroscopy. The progressive intensification of the N‐O antisymmetric stretching vibration band at ≈1250 cm^−1^ during potential scanning from OCP to −1.05 V versus RHE (Figure 4d–f) confirms the transformation of NO_3_^−^ to NO_2_^−^ as a key mechanistic step.^[^ ^25^ ^]^ The rapid conversion kinetics from NO_3_^−^ to NO_2_^−^ can be validated by the weak absorption peak of NO_3_^‒^ at ≈1382 cm^‒1^, which is ascribed to the N‐O asymmetric stretching.^[^ ^53^ ^]^ Simultaneously, the formation of NH_4_^+^ reaction product is verified by the absorption peak at ≈1460 cm^‒1^.^[^ ^54^, ^55^ ^]^ The peak at ≈1172 cm^‒1^ is attributed to the M‐N‐O (M = Fe/Cu) vibration of hydroxylamine (NH_2_OH),^[^ ^53^ ^]^ while the peaks at ≈1528 and 1320 cm^‒1^ are attributed to the bending vibration of N‐O‐H and the vibration of N‐H, respectively.^[^ ^55^, ^56^, ^57^ ^]^ Furthermore, the water‐splitting process, which supplies H^*^ for the deoxygenation and hydrogenation of NO_3_^‒^ in *e*‐NO_3_RR, is verified by the presence of the peak at ≈1648 cm^‒1^.^[^ ^58^, ^59^ ^]^ It is worth mentioning that CuFe_x_‐NC implies distinct peaks at ≈ 1690 and 1740 cm^‒1^, assigned to NO top‐site adsorption in contrast to the Cu‐NC and Fe‐NC.^[^ ^55^, ^60^ ^]^ This observation suggests a transition from bridge to top‐site NO adsorption configuration, where top‐adsorbed NO exhibits enhanced reactivity for H.^[^ ^61^, ^62^ ^]^ Combining the identified intermediates by both in situ FTIR and DEMS, it can be determined that the reaction pathway is as follows: ^*^NO_3_ → ^*^NO_2_ → ^*^NO → ^*^NOH→ ^*^NH_2_O → ^*^NH_2_OH → ^*^NH_2_ → ^*^NH_3_ (Figure 4g).

### 2.4 Theoretical Analysis

**Figure 5:**
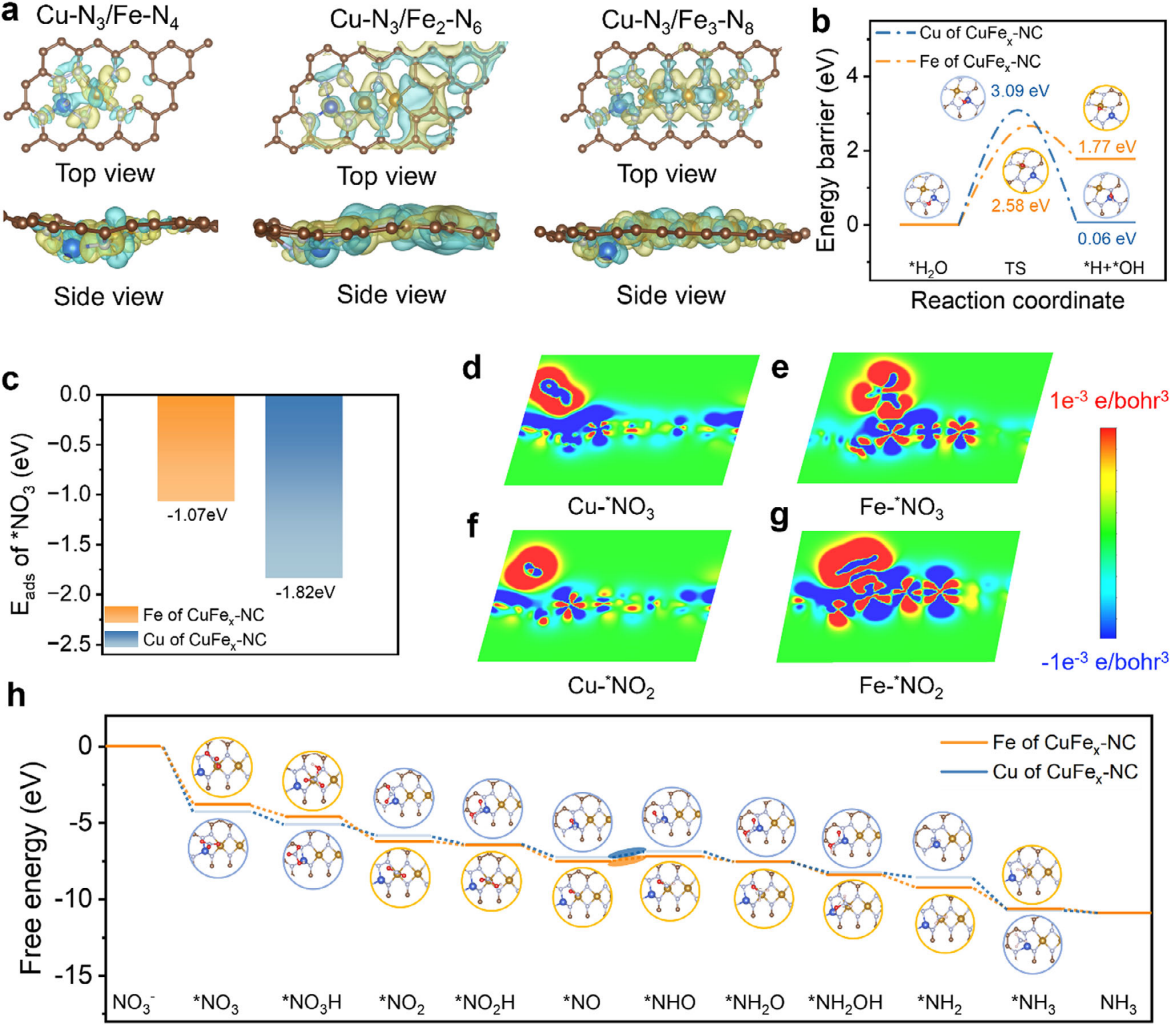
Theoretical calculations. a) Differential charge density of three models with different Fe atom numbers. b) The kinetic energy barriers for H_2_O activation on Cu and Fe sites of CuFe_x_‐NC. c) Adsorption energy of NO_3_^─^ and NO_2_^─^ at different metal sites on CuFe_x_‐NC. Differential charge density: adsorbed species of ^*^NO_3_ on d) Cu site and e) Fe site of CuFe_x_‐NC, and adsorbed species ^*^NO_2_ on f) Cu site and g) Fe site of CuFe_x_‐NC (blue and red represent charge depletion and accumulation, respectively). h) Free energy profiles of CuFe_x_‐NC (Cu and Fe sites) for *e*‐NO_3_RR.

To clarify the reaction mechanism as well as understand the reason of the remarkable *e*‐NO_3_RR activity of CuFe_x_‐NC, density functional theory (DFT) calculations were performed. The advantages of the Fe_3_‐N_8_ cluster were evaluated by analyzing the differential charge distributions of three structural models (Cu‐N_3_/Fe‐N_4_, Cu‐N_3_/Fe_2_‐N_6_, and Cu‐N_3_/Fe_3_‐N_8_) with varying Fe atom configurations (**Figure** **5**a; Figure S34, Supporting Information). Comparative analysis reveals enhanced charge accumulation around Fe atoms in Cu‐N_3_/Fe_3_‐N_8_ configuration compared to the Cu‐N_3_/Fe‐N_4_ and Cu‐N_3_/Fe_2_‐N_6_, creating electron‐rich regions that promote NO_2_^−^ binding. In addition, the charge depletion at Cu sites is more pronounced than in diatomic arrangements, leading to electron‐deficient regions that facilitate NO_3_^−^ adsorption.^[^ ^63^ ^]^ Bader charge plots reveal that the Fe sites adjacent to the Cu‐N_3_ moieties serve as the electron‐rich centers, accumulating electrons from both the copper and neighboring Fe sites (Figure S35, Supporting Information). This asymmetric charge redistribution effectively stabilizes the system by reducing its total energy, thereby significantly enhancing the thermodynamic stability of the tri‐nuclear FeN_4_ configuration.

In light of the critical role of ^*^H in *e*‐NO_3_RR, the kinetics of water dissociation at Cu and Fe sites were investigated. As demonstrated in Figure 5b and Figure S36 (Supporting Information), the calculations reveal a strong preference for ^*^H formation at Fe sites, with an activation energy barrier of 2.58 eV compared to 3.09 eV at Cu sites. According to the adsorption energy results of ^*^NO_3_ and ^*^NO_2_ intermediates on CuFe_x_‐NC metal sites (Figures S37–S39, Supporting Information), it is evident that NO_3_^−^ is preferentially adsorbed at Cu sites (−0.972 eV) over Fe sites (Fe_1_, −0.70 eV). Conversely, Fe_1_ sites exhibited a greater binding affinity for NO_2_^−^ (Figure 5c; Table S9, Supporting Information). Analysis of the charge density difference between the metal sites (Cu and Fe_1_) and the intermediates (^*^NO_3_ and ^*^NO_2_) (Figure 5d–g) shows that ^*^NO_3_ binding enhances electron transfer at Cu sites (Figure S40, Supporting Information), whereas ^*^NO_2_ binding at the Fe1 site has a greater binding strength (Figure S41, Supporting Information). These findings demonstrate that synergistic dual‐site interactions between Cu single atoms and Fe clusters simultaneously enhance both water activation and nitrate adsorption capabilities. To elucidate the *e*‐NO_3_RR pathway involving hydrogenation and stepwise deoxygenation on CuFe_x_‐NC, free energy calculations were performed (Figure 5h). Preferential NO_3_^−^ adsorption at Cu sites with a lower initial free energy barrier (ΔG = −4.28 eV) compared to the Fe sites (−3.8 eV) was identified. The transition from ^*^NO_3_H→^*^NO_2_ shows prominent site‐dependent variations in ΔG. It is worth noting that N‐containing intermediates migrate favorably between the metal sites, as the rate‐determining step (RDS) of ^*^NO→^*^NHO displays a lower activation energy at Fe sites (0.32 eV) than Cu sites (0.39 eV). It means that the process enables the sequential conversion of NO_3_^−^ to NO_2_^−^ at Cu‐sites, followed by binding at Fe‐sites for subsequent reduction steps. Considering all these insights, we can conclude that the advanced *e*‐NO_3_RR activity of CuFe_x_‐NC (Cu‐N_3_/Fe_3_‐N_8_ configuration) arises from optimized intermediate adsorption energetics during this relay catalysis process.

## 3 Conclusion

This study demonstrates the development of a synergistic dual‐metal‐site tandem catalyst for efficient *e*‐NO_3_RR. The CuFe_x_‐NC catalyst reveals exceptional performance, with a Faraday efficiency of 97.1% and an NH_3_ yield of 18.83 mg∙h^‒1^ ∙mg_cat_^‒1^ at −0.65 V versus RHE. Through a comprehensive mechanistic investigation combining in situ DEMS, in situ FTIR, EPR spectroscopy, and DFT calculations, we provide in‐depth mechanistic insights into the reaction pathways. It reveals that the exceptional *e*‐NO_3_RR performance of the Cu‐N_3_/Fe_3_‐N_8_ configuration arises from synergistic interactions between Fe clusters and Cu single‐atom sites, where H^*^ species is produced on Fe sites, promoting NO_3_^−^ reduction, while Cu sites favor the formation of NO_2_^−^, which diffuses to Fe sites for H^*^‐coupled NH_3_ production. This work is expected to provide fundamental design principles of tunable bonding interactions at dual‐metal sites with various reactants and intermediates, and provide a strategic framework for the development of high‐performance tandem catalysts with cooperative enhancement effects.

## Conflict of Interest

The authors declare no conflict of interest.

## Author Contributions

Y.L. and X. Z. contributed equally to this work. C.W. and X.W. directed the project. Y.L. and X.Z. performed materials synthesis, beach‐scale degradation experiments, and electrochemical experiments. H.C. and Y.Z. ran XAS data testing. X.Z. analyzed the results of FTIR experiments. L.L. and M.L. carried out and analyzed the DFT calculations. S.T. and C. L. conducted the DEMS test. W.H. performed AC‐TEM images. Y.L., F.S., M.H., D.W., K.H., Y.P., X.W., C.W. wrote the paper.

## Supporting information



Supporting Information

## Data Availability

The data that support the findings of this study are available from the corresponding author upon reasonable request.
