# Supplementary material for: Embedded Fe‐Cu Pairs Enable Tandem Nitrate‐to‐Ammonia Electroreduction
Source: Adv Mater. 2025 Oct 2;38(3):e14840. doi: 10.1002/adma.202514840 (PMC12801372; doi:10.1002/adma.202514840)
Supplement: Supplementary file 1 — Supporting Information [file ADMA-38-e14840-s001.docx]

Supporting Information

**Embedded Fe-Cu pairs enable tandem nitrate-to-ammonia electroreduction**

Yuxiao Liu, Xia Zhang, Solmaz Feizpoor, Hsiao-Chien Chen, Linfeng Li, Yunpeng Zuo, Shengji Tian, Mengni Liu, Wenyu Hu, Muhammad Humayun, Kaifu Huo, Chade Lv, Yuanjie Pang, Dingsheng Wang, Xin Wang*, Chundong Wang*

**Experimental section**

**1. Materials preparation**

*Materials and Chemicals:* 2-methylimidazole (MelM), zinc nitrate hexahydrate (Zn(NO_3_)_2_∙6H_2_O), cupper(Ⅱ) nitrate trihydrate (Cu(NO_3_)_2_∙3H_2_O), ferric(Ⅲ) nitrate nonahydrate (Fe(NO_3_)_3_∙9H_2_O), maleic acid (C_4_H_4_O_4_), sodium citrate (C_6_H_5_Na_3_O_7_), and ammonium chloride-^15^N (^15^NH_4_Cl) were purchased from the Aladdin Lt. Iron(Ⅲ) chloride hexahydrate (FeCl_3_∙6H_2_O), sodium sulfate (Na_2_SO_4_), sodium nitrate (NaNO_3_), sodium nitrite (NaNO_2_), methanol (CH_3_OH), salicylic acid (C_7_H_6_O_3_), sodium hydroxide (NaOH), sodium hypochlorite aqueous solution (NaClO), phosphoric acid (H_3_PO_4_), sulfamic acid (H_3_NO_3_S), and hydrochloric (HCl) were acquired from the Sinopharm Chemical Reagent Co., Ltd. Hexane (C_6_H_14_), ethanol (C_2_H_5_OH), and sulfanilamide (C_6_H_8_N_2_O_2_S) were supplied by the Cologne Chemicals Ltd. Nafion ® D-520 dispersion (5%w/w in water and 1-propanol, >1meq/g exchange capacity), deuterium oxide (D_2_O), and N-(1-Naphthyl) ethylenediamine dihydrochloride (C_12_H_16_Cl_2_N_2_) were purchased from 3A Materials ®(3A). Sodium nitrate-^15^N (Na^15^NO_3_) and sodium nitroprusside dihydrate (C_5_H_4_FeN_6_Na_2_O_3_) were obtained from D&B Ltd.

*Synthesis of CuFe_x_-NC and Reference Electrocatalysts:*

*Synthesis of Cu-ZIF:* Briefly, 1.97 g of 2-methylimidazole was ultrasonically dissolved in 120mL of methanol for 10 minutes, and then subjected to 120 mL of methanol solution containing 1.695 g Zn (NO_3_)_2_∙6H_2_O and 180 mg Cu (NO_3_)_2_∙3H_2_O. The above solution was stirred in a water bath at 60℃ for 12h. Afterward, the obtained precipitate was centrifuged, washed with methanol several times, and dried overnight under vacuum at 60℃ to obtain Cu-ZIF.

*Synthesis of CuFe_x_-NC:* About 80 mg of Cu-ZIF was ultrasonically uniformly dispersed in 13mL of n-hexane for 1h, then 50µL FeCl_3_∙6H_2_O aqueous solution containing 20 mg∙mL^‒1^ was added to it and stirred for 3h. Then, the obtained precipitate was centrifuged, washed with ethanol several times, and dried overnight under vacuum at 80℃. The collected samples were subjected to a tubular furnace, treated at 950℃ with a heating rate of 5℃/min, and then maintained for 2h under Ar atmosphere, to obtain CuFe_x_-NC product.

*Synthesis of Cu-NC:* The collected Cu-ZIF was put into a tube furnace and underwent the same heat treatment as that of CuFe_x_-NC. Finally, the control sample Cu-NC was obtained.

*Synthesis of Fe-NC:* Fe-NC was synthesized in a similar way to Cu-NC, where the copper source (Cu (NO_3_)_2_∙3H_2_O) was replaced by an iron source (Fe (NO_3_)_3_∙9H_2_O).

**2. Material Characterizations**

The Bruker AXS D8 diffractometer (Cu K*α* X-ray source) was used to record X-ray powder diffraction (XRD) patterns within the range of 5–80°. FTIR spectra were collected with a Bruker Tensor-27 in transmittance mode. A ZEISS G300 system was employed to conduct the scanning electron microscopy (SEM) measurement. Transmission electron microscopy (TEM), high-resolution TEM (HR-TEM), and energy dispersive spectroscopy (EDS) were implemented using a FEI Tecnai G2 F30 with an acceleration voltage of 200 kV. The spherical aberration corrected scanning transmission electron microscope (AC-STEM) was collected on Titan Cubed Themis G2 300 with an acceleration voltage of 300 kV. X-ray photoelectron spectroscopy (XPS) spectra were acquired using the Kratos AXIS Ultra DLD-600 W, after calibration with C 1 s peak at 284.8 eV. The Bruker EMX plus was used to operate electron paramagnetic resonance (EPR) spectroscopy. The BET surface area and BJH pore volumes were evaluated using N_2_ adsorption-desorption isotherms (TriStar 20). The Raman spectra of the as-prepared catalysts were measured using an Invia spectrometer with a custom-made electrochemistry cell (Gaoss Union C301-3) having a wavelength of 532 nm. ^1^H nuclear magnetic resonance (NMR) spectra were collected on Bruker AscendTM 600 MHZ. The absorbance was measured by a UV-1800SPC double beam ultraviolet/visible spectrophotometer.

**3. XAFS measurements and data analysis**

X-ray absorption spectroscopy (XAS) measurements were conducted at the SPring-8 12B2 Taiwan beamline of the Synchrotron Radiation Research Center (NSRRC) with total fluorescence yield mode under ambient conditions. XAFS data processing followed standard Athena procedures,^[1]^ including normalization and background subtraction, using iron and copper reference samples for energy calibration. k^3^-weighted EXAFS spectra were obtained through edge-jump normalization and back-edge background subtraction. Cu and Fe K-edge χ(k) data were transformed from k-space to R-space using Hanning windows (dk = 1.0 Å^‒1^) to investigate coordination shell contributions. Quantitative structural parameters were derived through EXAFS fitting analysis using the Artemis software package.^[1]^ Wavelet transform (WT) analysis of EXAFS data was performed using the Hama Fortran code,^[2-3]^ with parameters spanning R: 1-3 Å and k: 2-10 Å^‒1^. XANES theoretical calculations were implemented using the FDMNES code under a specified analysis model.^[4-5]^

**4. Electrochemical measurements**

Electrochemical nitrate reduction performance was evaluated using a CHI 760E electrochemical workstation in a three-electrode configuration with a bipolar membrane-separated H-type cell. The catalyst ink was prepared by ultrasonically dispersing 5 mg of catalyst in a solution containing 480 μL of ethanol, 480 μL of deionized (DI) water, and 40 μL of Nafion solution (5 wt%) for 1 h, to achieve homogeneous dispersion. The prepared ink was drop-cast onto a 1 × 1 cm^2^ carbon cloth substrate and dried at ambient temperature. The electrochemical cell employed a Pt sheet as the counter electrode and a Hg/HgCl as reference electrode in an electrolyte comprising 0.5 M Na_2_SO_4_ and 0.1 M NaNO_3_. All potentials were calibrated to a reversible hydrogen electrode (RHE) by the following formula:

$$E_{\text{RHE}}\text{ = }E_{\text{Hg/HgCl }}\text{+ 0.059}\times\text{pH + 0.2415 V}$$

Prior to measurements, electrolytes were purged with Ar gas for 30 minutes to eliminate O_2_ and N_2_. Cyclic voltammetry (CV) measurements were conducted at 10 mV∙s^−1^ until stable polarization curves were achieved. Linear sweep voltammetry (LSV) was performed at 5 mV∙s^−1^ in electrolyte containing 0.5 M Na_2_SO_4_ and 0.1 M NaNO_3_. NO_2_⁻ reduction studies were carried out under identical conditions using 0.1 M NaNO_2_ in Na_2_SO_4_ electrolyte. Chronoamperometric measurements were performed at various potentials for 30-minute intervals. Double-layer capacitance (C_dl_) was determined through CV measurements at multiple scan rates (1-5 mV∙s^−1^). C_dl_ values were calculated from the slope of Δj versus scan rate plots within a selected potential window, where Δj represents the difference between anodic (j_a_) and cathodic (j_c_) current densities. The resulting slope corresponds to twice the C_dl_ value.

**5. Isotope labeling experiments**

Isotope-labeled nitrate reduction experiments were conducted using Na^15^NO_3_ to verify the ammonia source. It contained 0.1 M Na^15^NO_3_ in the cathode chamber with 0.5 M Na_2_SO_4_ working as the supporting electrolyte. Post-reduction electrolyte containing ^15^NH_4_^+^ was collected and adjusted to pH 3.5 using 3 M HCl. Quantitative analysis was performed via ^1^H NMR spectroscopy (600 MHz) using maleic acid as an external standard. The calibration procedure involves preparation of standard solutions of ^15^NH_4_Cl (40-200 ppm) in 0.5 M Na_2_SO_4_. Aliquots (50 mL) of these standards were mixed with maleic acid (0.02 g). For NMR analysis, 0.5 mL of each mixture was combined with 50 μL deuterium oxide (D_2_O). Quantification was achieved using the peak area ratio between ^1^H-^15^N and maleic acid signals. This methodology was similarly applicable for ^14^NH_4_^+^ quantification when using Na^14^NO_3_ as the nitrogen source.

**6. Determination of ammonia**

Ammonia quantification was performed using the indophenol blue method. Post-electrolysis electrolyte samples were diluted to a detectable range. The colorimetric assay was conducted by combining 2 mL of diluted electrolyte with 2 mL of complexing reagent (1 M NaOH containing 5 wt% salicylic acid and 5 wt% sodium citrate). Subsequently, 1 mL of NaClO solution (3 wt%) and 0.2 mL of sodium nitroferricyanide solution (1 wt%) were sequentially added. After 2 h incubation under dark conditions, UV-visible spectroscopy analysis was performed, with NH_4_^+^ concentration determined from absorbance measurements at 655 nm. Calibration curves were established using standard NH_4_Cl solutions (with specific known concentrations).

**7. Determination of nitrite**

Nitrite (NO_2_⁻) concentration was determined via colorimetric analysis using the coupling reaction between p-aminobenzamide and N-(1-naphthyl) ethylenediamine dihydrochloride under acidic conditions. The chromogenic reagent was prepared by dissolving N-(1-naphthyl) ethylenediamine dihydrochloride (0.2 g), p-aminobenzamide (4 g), and phosphoric acid (10 mL) in DI water (50 mL) under continuous stirring. The colorimetric assay was performed by combining the chromogenic reagent (0.1 mL) with diluted electrolyte (1 mL), followed by 30-minute incubation at ambient temperature. NO_2_⁻ concentration was determined spectrophotometrically at 540 nm, with calibration curves plotted using standard NaNO_2_ solutions.

**8. Determination of nitrate**

Nitrate (NO_3_⁻) quantification was performed by UV spectrophotometric analysis. The analytical solution was prepared by combining diluted electrolyte (5 mL) with 1 M HCl (0.1 mL) and 0.8 wt% sulfamic acid solution (10 μL), then diluting to 10 mL under continuous stirring. Following 20-minute equilibration at ambient temperature, absorbance measurements were recorded at 220 nm and 275 nm. NO_3_⁻ concentration was calculated using the relationship A=A_200_-2×A_275_. Calibration curves were established using standard NaNO_3_ solutions.

**9. Determination of H_2_**

The hydrogen concentration in the headspace was precisely measured by In-situ gas chromatography electrochemical system (GIS-GC-1). The hydrogen gas content was quantitatively analyzed using the headspace method. Specifically, the total volume of the sealed H-type electrochemical cell was determined, from which the headspace volume was calculated by subtracting the volume occupied by the electrolyte solution.

**10. Calculation of Faradaic efficiency and yield rate**

The Faraday efficiency (FE) of NH_3_ was calculated using the following formula:

$$\text{FE}_{\text{NH}_{3}}\text{= }\frac{n_{\text{NH}_{3}}\times V\times F}{Q}$$

The Yield rate of NH_3_ was determined according to the following equation:

$$\text{Y}_{\text{NH}_{3}}\text{ = }\frac{C\times V}{t\times A\times m_{cat}}$$

The Faraday efficiency for H_2_ was calculated using the following formula:

$$\text{FE}(\text{H}_{2})=\frac{\frac{\text{V}_{\text{HS}}}{\text{V}_{\text{m}}}\times n_{H_{2}}\times G\%\times F}{Q}$$

Where F is Faraday's constant (96485 C∙mol^‒1^), C is the measured concentration of NH_3_ in the electrolyte, V is the volume of electrolytic liquid, V_HS_ is the volume of the headspace, V_m_ represents the volume of 1 mole of air at room temperature, n represents the number of transferred electrons, Q is the amount of electricity applied during electrolysis, G represents the hydrogen content, t is the electrolytic time (h), and A is the geometric area of the working electrode (1 cm^‒2^). m_cat_ is the loading capacity of catalyst (0.25 mg∙cm^‒2^).

**11. Electrochemical *in situ* FTIR spectrometry**

*In situ* Fourier transform infrared (FTIR) spectroscopy was conducted using a Bruker Tensor spectrometer coupled with a three-electrode H-cell configuration. The electrochemical cell comprised a catalyst-modified working electrode, platinum sheet counter electrode, and Hg/HgCl reference electrode in an electrolyte containing 0.5 M Na_2_SO_4_ and 0.1 M NaNO_3_. Background spectra were collected under open circuit potential following cyclic voltammetry activation of the catalyst. FTIR spectra were subsequently recorded at applied potentials ranging from -1.005 to -1.705 V versus Saturated calomel electrode (SCE) at 100 mV intervals.

**12. DEMS measurements**

DEMS measurements were performed using a differential electrochemical mass spectrometry (PM-DEMS, Shanghai Jingpu Ruo Technology Co., Ltd). The electrochemical cell composes a catalyst working electrode, Hg/HgCl reference electrode, and platinum sheet counter electrode, which was working in an electrolyte containing 0.5 M Na_2_SO_4_ and 0.1 M NaNO_3_. Continuous argon purging was maintained before and during measurements to eliminate atmospheric gases (N_2_ and O_2_). Mass-to-charge ratio signals were monitored during 60 seconds chronoamperometric measurements at peak Faradaic currents for each catalyst. During the measurement, the mass spectrometer signals were allowed to return to the baseline level. Measurements were repeated in quadruplicate under identical conditions to ensure the reproducibility.

**13. DFT calculations.**

DFT calculations were performed using the VASP package,^[6-7]^ with the Perdew−Burke−Ernzerhof (PBE) generalized gradient approximation (GGA) for the exchange-correlation potential.^[8]^ Electron-ion interactions were treated with the projector augmented wave (PAW) method.^[9]^ A cutoff energy of 400 eV was applied, and the Brillouin zone was sampled using a 3×3×2 Monkhorst-Pack k-point grid. Convergence criteria for energy and force in the self-consistent cycle were set at 10^‒4^ eV and -0.02 eV Å^‒1^, respectively.

The following pathways for CuFe_x_-NC involve the intermediates of *NO_3_, *NO_3_H, *NO_2_, NO_2_H, *NO, *NHO, *NH_2_O, *NH_2_OH, *NH_2_, and *NH_3_. The optimal reaction process is represented by the following formula:

$$\text{* + NO}_{3}^{-}\to*\text{NO}_{3}+\text{e}^{-}$$

$$\text{*NO}_{3}^{-}+\text{H}^{\text{+}}\text{ + e}^{-}\to*\text{NO}_{3}\text{H}$$

$$*\text{NO}_{3}\text{H}+\text{H}^{\text{+}}\text{ + e}^{-}\to*\text{NO}_{2}+\text{H}_{2}\text{O}$$

$$\text{*NO}_{2}+\text{H}^{\text{+}}\text{ + e}^{-}\to*\text{NO}_{2}\text{H}$$

$$*\text{NO}_{2}\text{H}+\text{H}^{\text{+}}\text{ + e}^{-}\to*\text{NO}+\text{H}_{2}\text{O}$$

$$\text{*NO}+\text{H}^{\text{+}}\text{ + e}^{-}\to*\text{NHO}$$

$$*\text{NHO}+\text{H}^{\text{+}}\text{ + e}^{-}\to*\text{NH}_{2}\text{O}+\text{H}_{2}\text{O}$$

$$*\text{NH}_{2}\text{O}+\text{H}^{\text{+}}\text{ + e}^{-}\to*\text{NH}_{2}\text{OH}$$

$$*\text{NH}_{2}\text{OH}+\text{H}^{\text{+}}\text{ + e}^{-}\to*\text{NH}_{2}+\text{H}_{2}\text{O}$$

$$\text{*NH}_{2}+\text{H}^{\text{+}}\text{ + e}^{-}\to*\text{NH}_{3}$$

$$*\text{NH}_{3}\to*+\text{NH}_{3}$$

Where * represents the active site.

The Gibbs free energy changes (ΔG) of the reaction are calculated according to the following equation:

$$\Delta\text{G}=\Delta\text{E + }\Delta ZPE-\text{T}\Delta\text{S}+\Delta\text{G}_{U}+\Delta\text{G}_{pH}$$

$$\Delta\text{G}_{\text{U}}\text{= -}eU$$

$$\Delta\text{G}_{\text{pH}}=\text{ }k_{B}T\times\ln10\times PH$$

where ΔE is the electronic energy difference directly obtained from DFT calculations, ΔZPE is the zero-point energy difference, T is the room temperature (298.15 K) and ΔS is the entropy change, U is the applied electrode potential, k_B_ is the Boltzmann constant, and pH value is set to 0.

The diffusion barriers for Li ion along the diffusion pathway are subsequently calculated by climbing image nudged elastic band (CI-NEB) methods.^[10]^


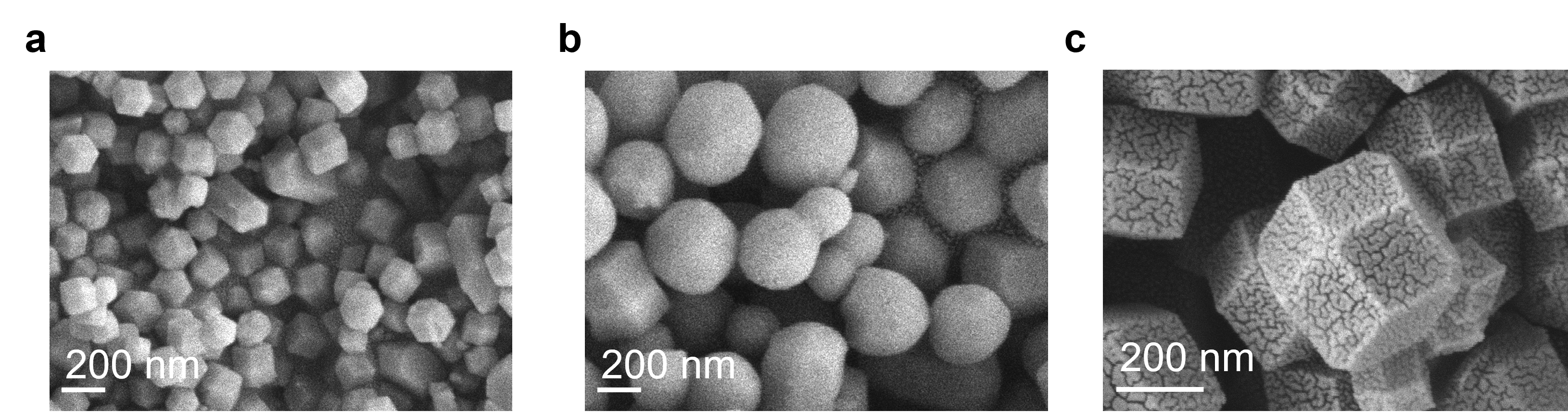


**Figure S1** SEM images of (a) Cu/ZIF, (b) Fe@Cu/ZIF, and (c) CuFe_x_-NC.


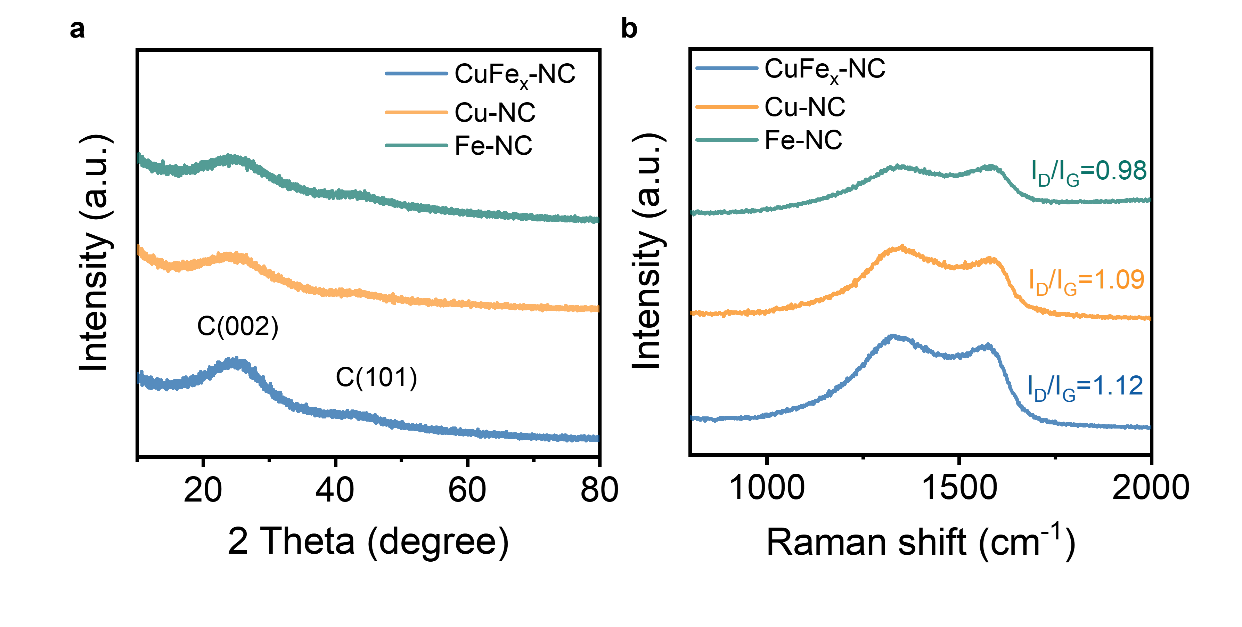


**Figure S2** (a) XRD patterns and (b) Raman spectra of CuFe_x_-NC, Cu-NC, and Fe-NC.


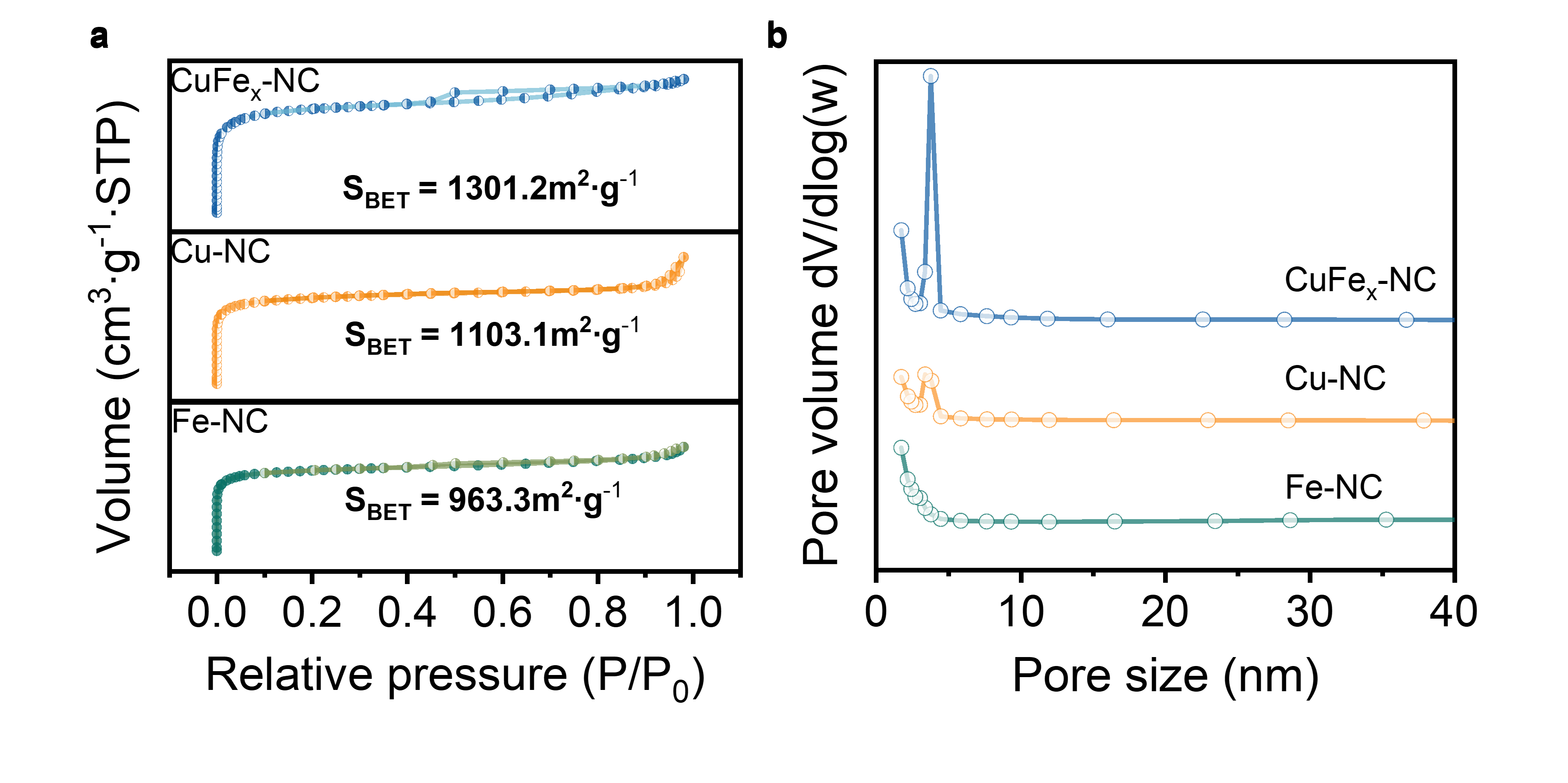


**Figure S3** (a) N_2_ adsorption and desorption isotherms, and (b) Pore diameter distributions of CuFe_x_-NC, Cu-NC, and Fe-NC.


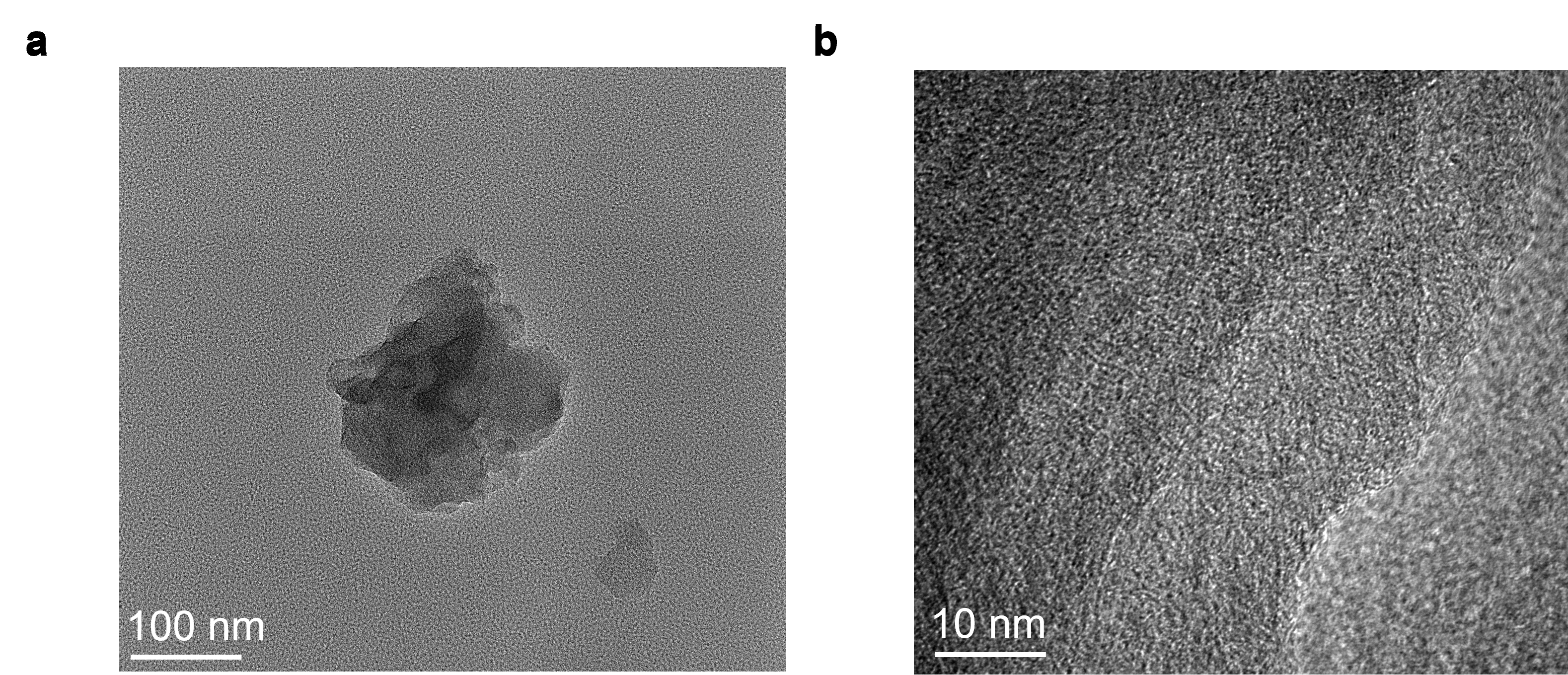


**Figure S4** (a) TEM and (b) HRTEM images of CuFe_x_-NC revealing randomly orientated graphitic layers without obvious metal particles.


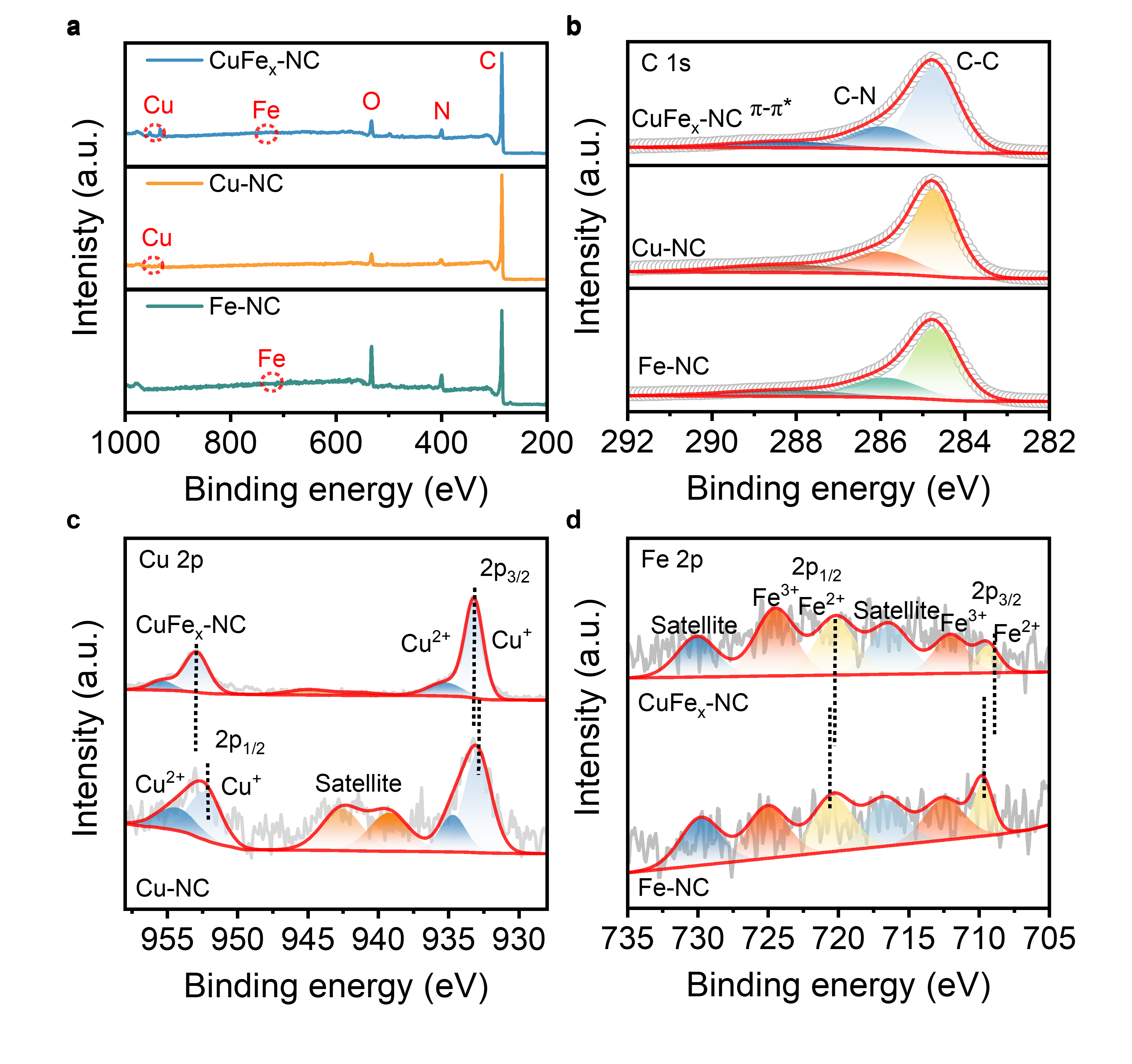


**Figure S5** (a) XPS survey spectra of CuFe_x_-NC, Cu-NC and Fe-NC. (b) C 1s spectra of CuFe_x_-NC, Cu-NC and Fe-NC, (c) Cu 2p spectra of CuFe_x_-NC and Cu-NC, (d) Fe 2p spectra of CuFe_x_-NC and Fe-NC.


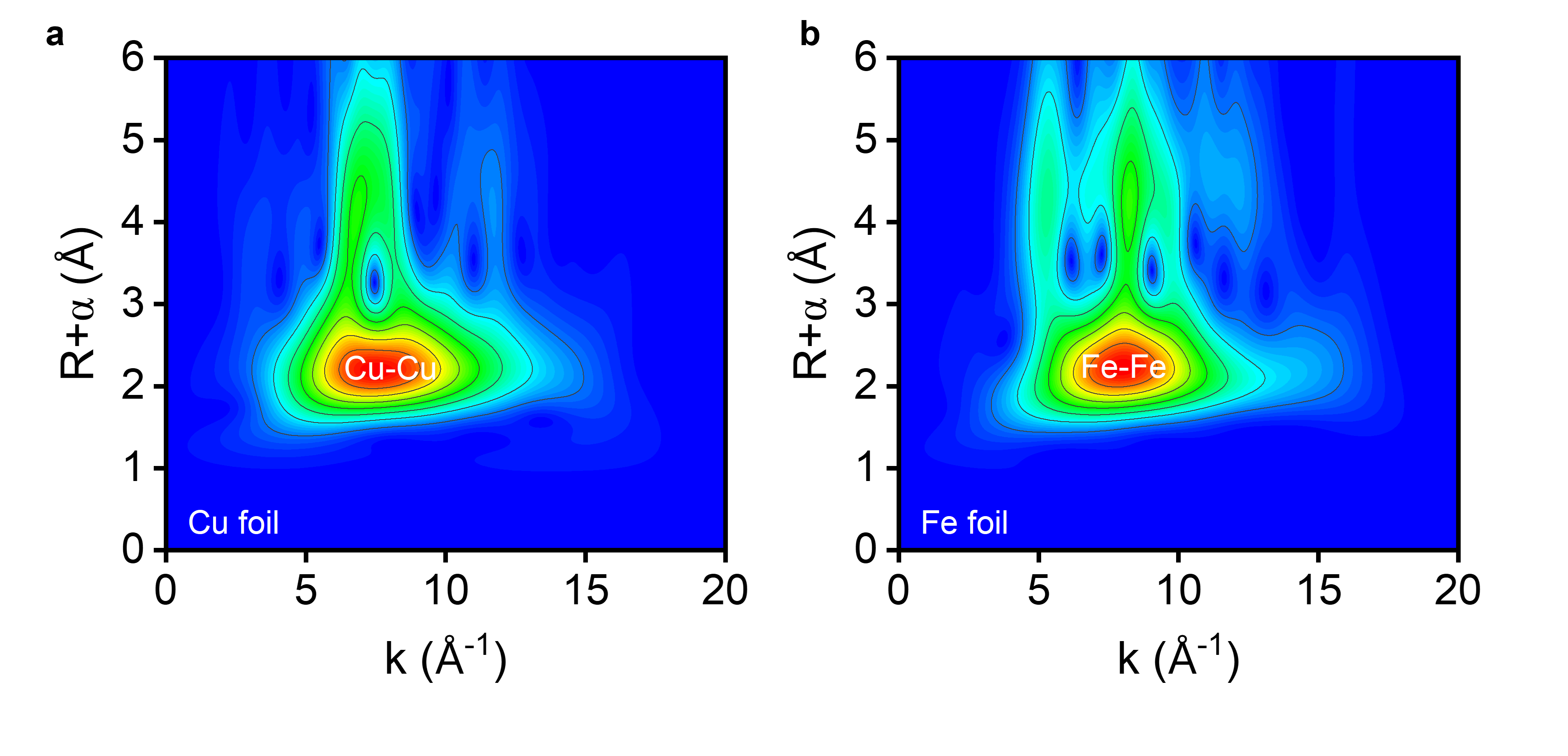


**Figure S6** WT-EXAFS of (a) Cu foil, and (b) Fe foil.


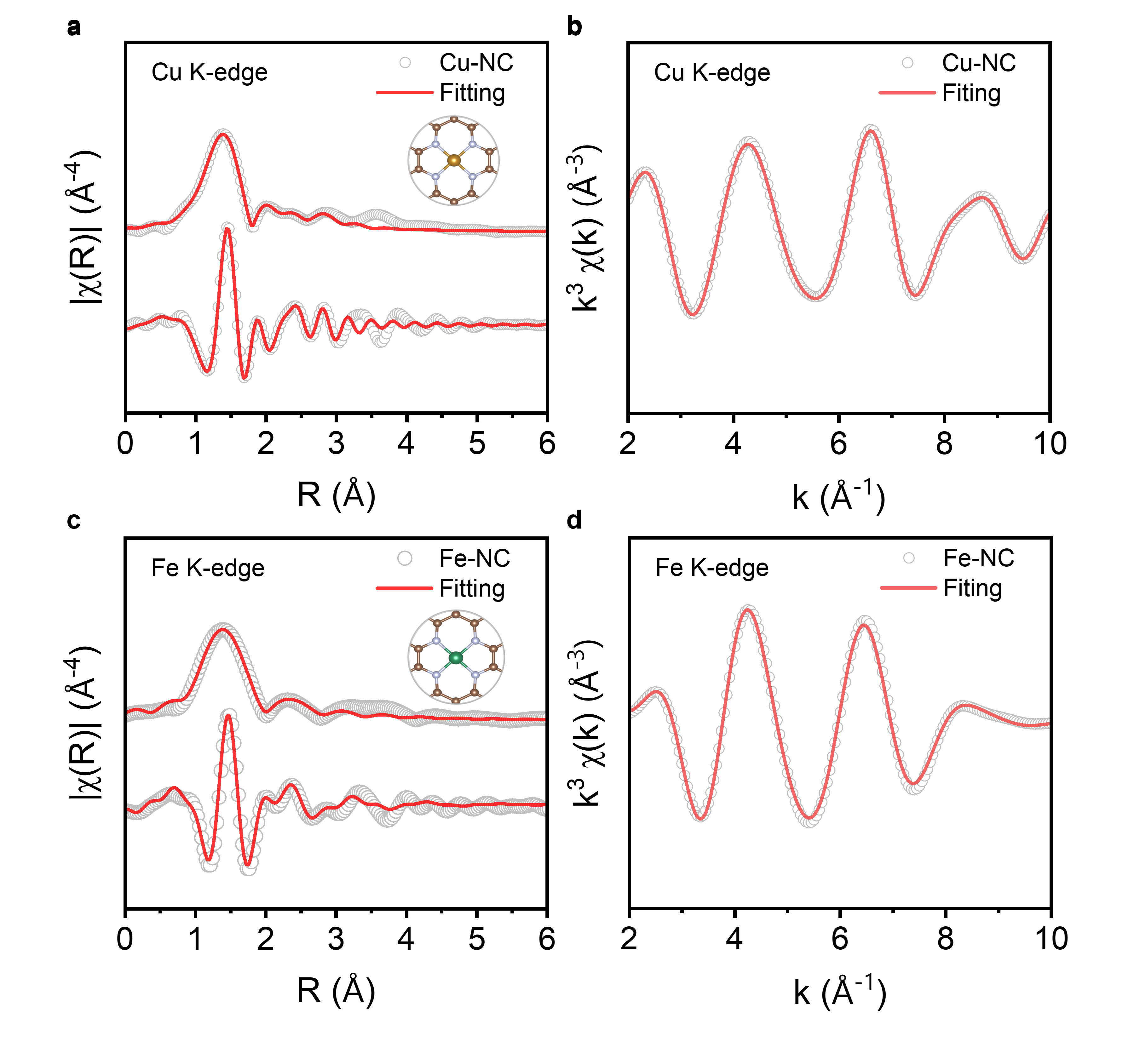


**Figure S7** (a, b) Cu K-edge experimental and FT-EXAFS fitting curves of Cu-NC and the corresponding fitting curves of k^3^-weighted and (c, d) Fe K-edge of Fe-NC.


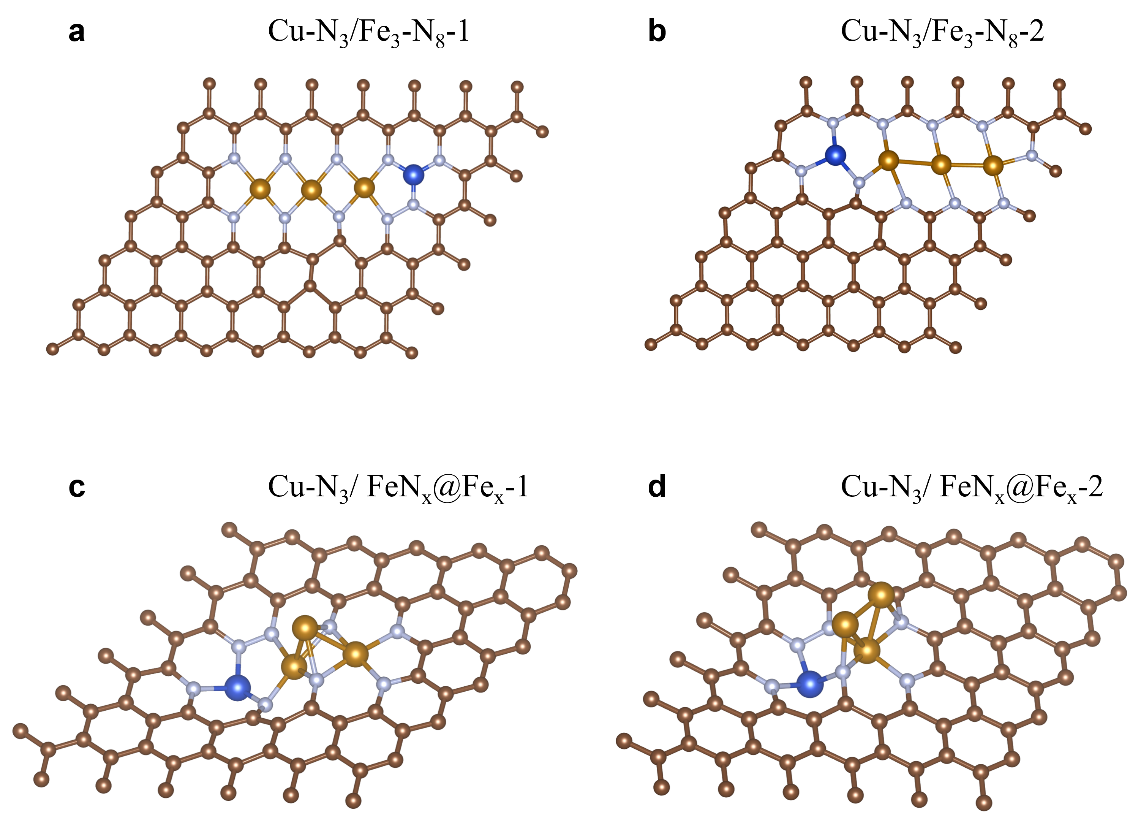


**Figure S8** The possible structural models of Fe cluster in CuFe_x_-NC.


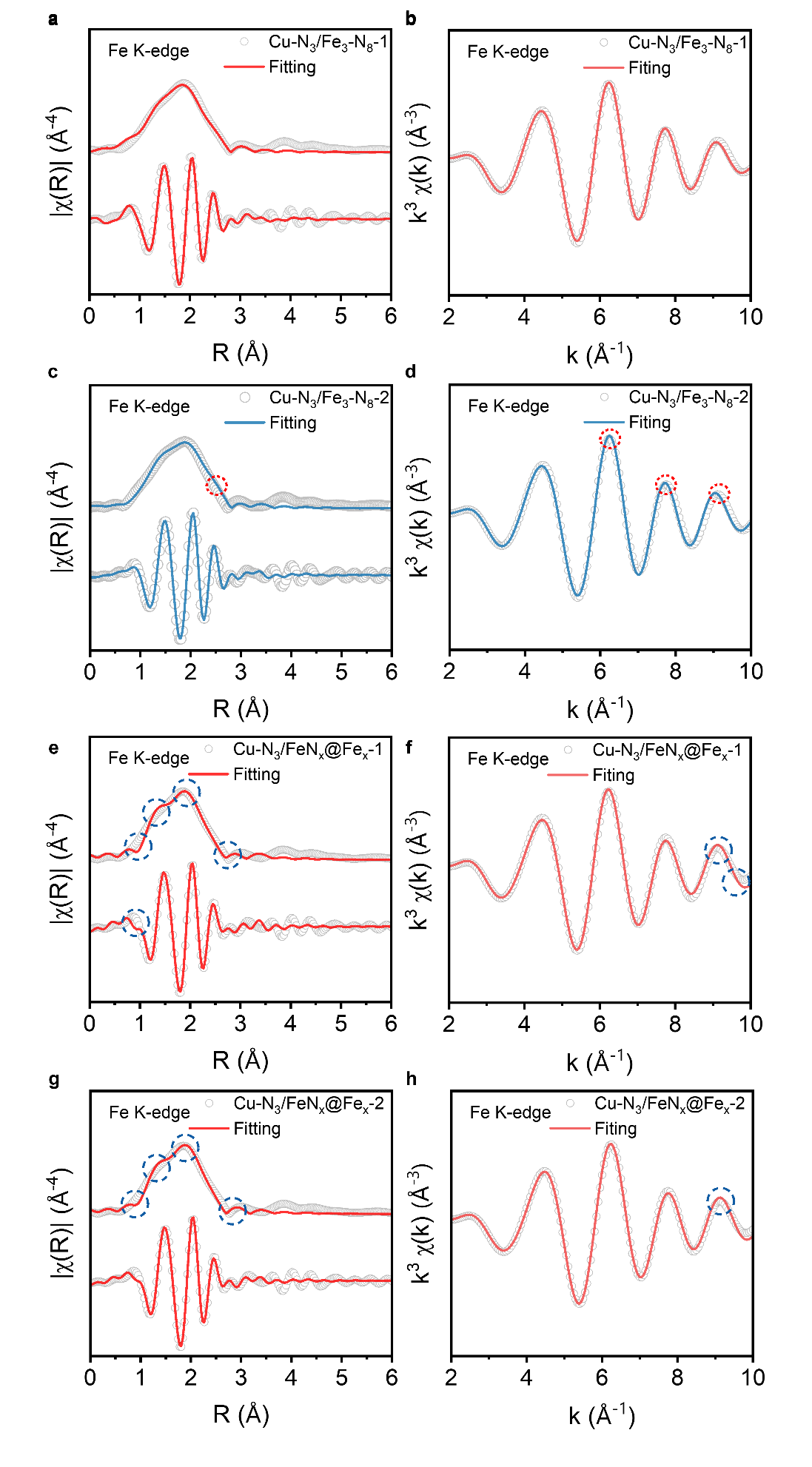


**Figure S9** (a, b) Fe K-edge experimental and FT-EXAFS fitting curves of CuFe_x_-NC and the corresponding fitting curves of k^3^-weighted k-space based on Fe_3_‒N_8_‒1 and (c, d) Fe_3_‒N_8_‒2, (e, f) Cu-N_3_/FeN_x_@Fe_x_-1 and (g, h) Cu-N_3_/FeN_x_@Fe_x_-2 models.


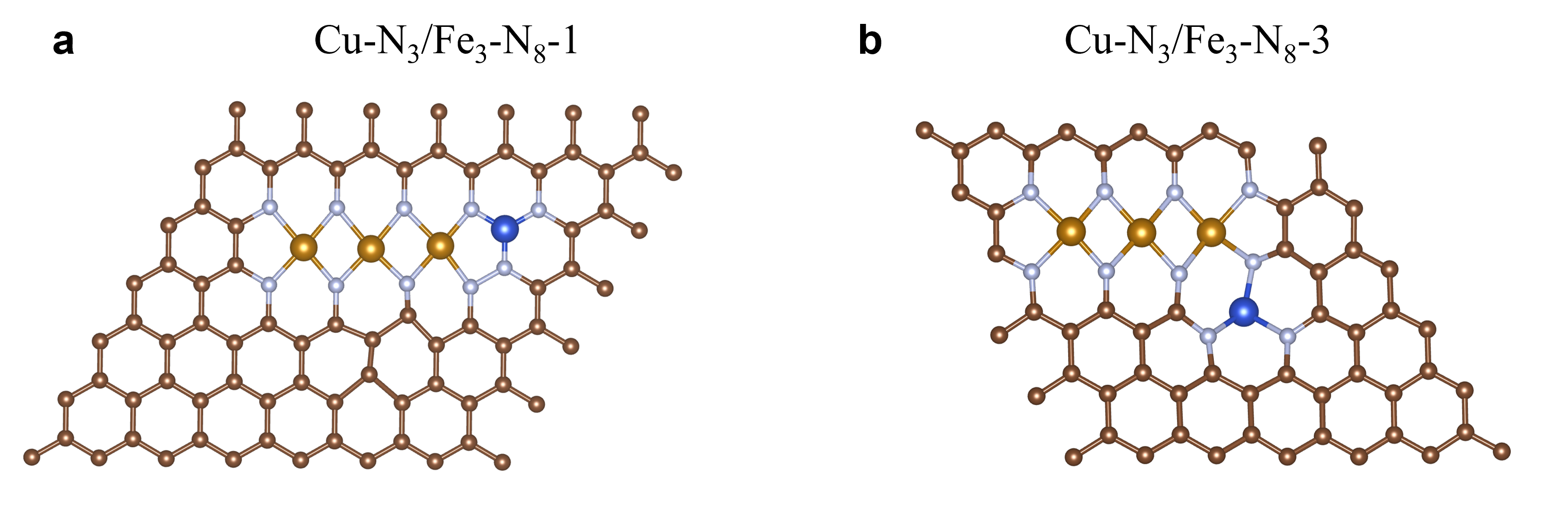


**Figure S10** The possible structural models of Cu-N_3_/Fe_3_-N_8_.


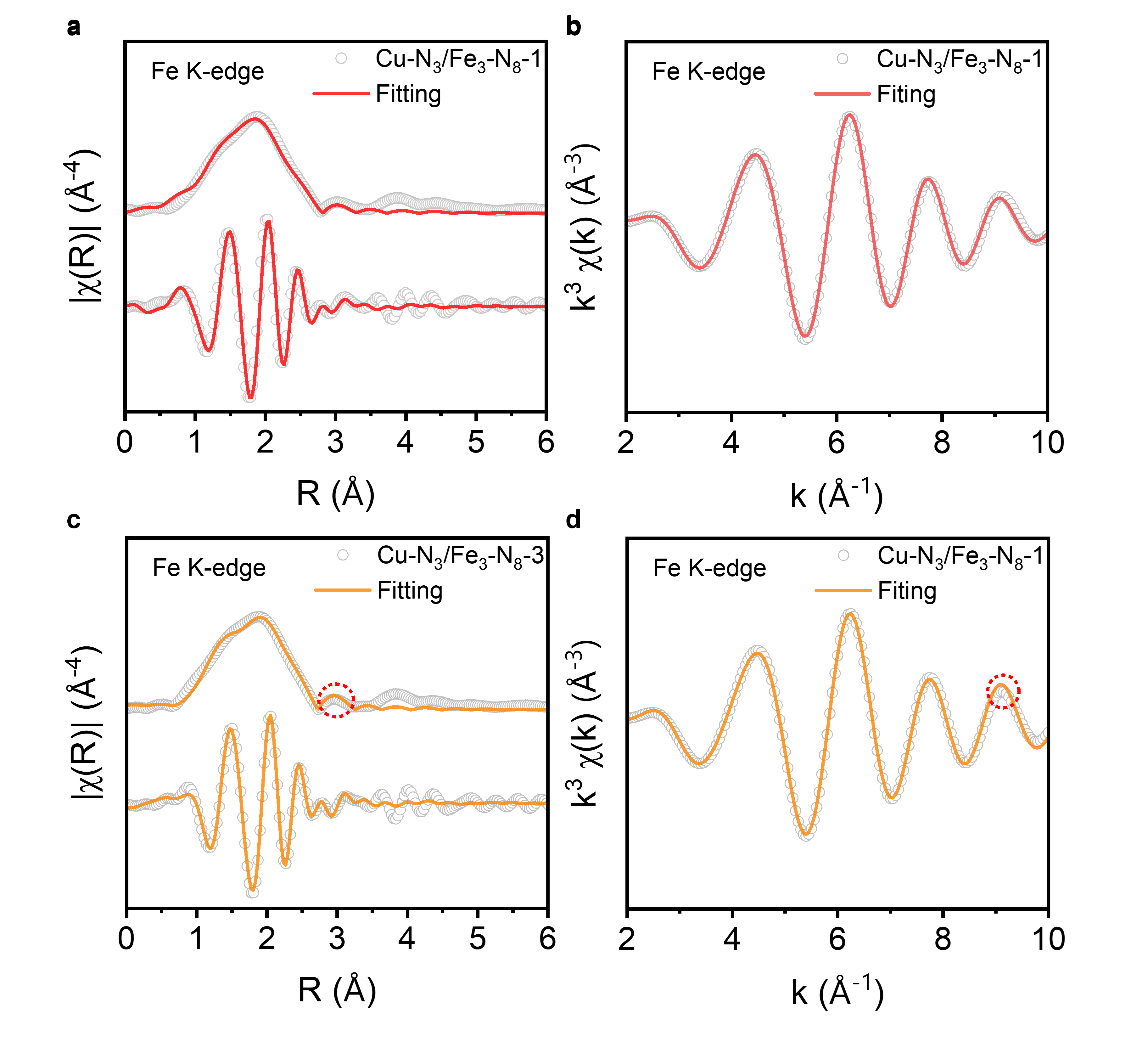


**Figure S11** (a, b) Fe K-edge experimental and FT-EXAFS fitting curves of CuFe_x_-NC and the corresponding fitting curves of k^3^-weighted k-space based on Fe_3_‒N_8_‒1 and (c, d) Fe_3_‒N_8_‒3 models.


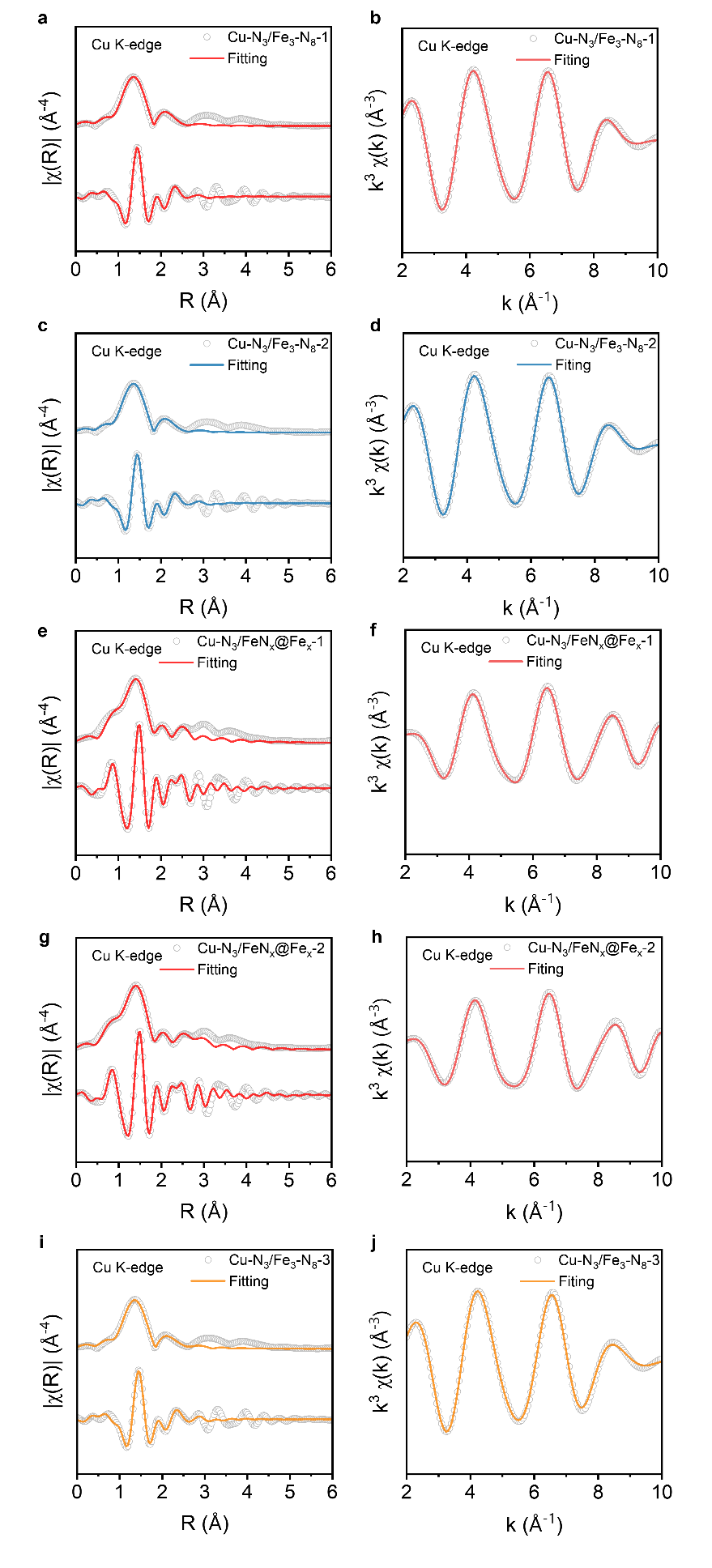


**Figure S12** (a, b) Cu K-edge experimental and FT-EXAFS fitting curves of CuFe_x_-NC and the corresponding fitting curves of k^3^-weighted k-space based on Cu-N_3_/Fe_3_‒N_8_‒1, (c, d) Cu-N_3_/Fe_3_‒N_8_‒2, (e, f) Cu-N_3_/FeN_x_@Fe_x_-1, (g, h) Cu-N_3_/FeN_x_@Fe_x_-2 and (i, j) Cu-N_3_/Fe_3_-N_8_-3 models.


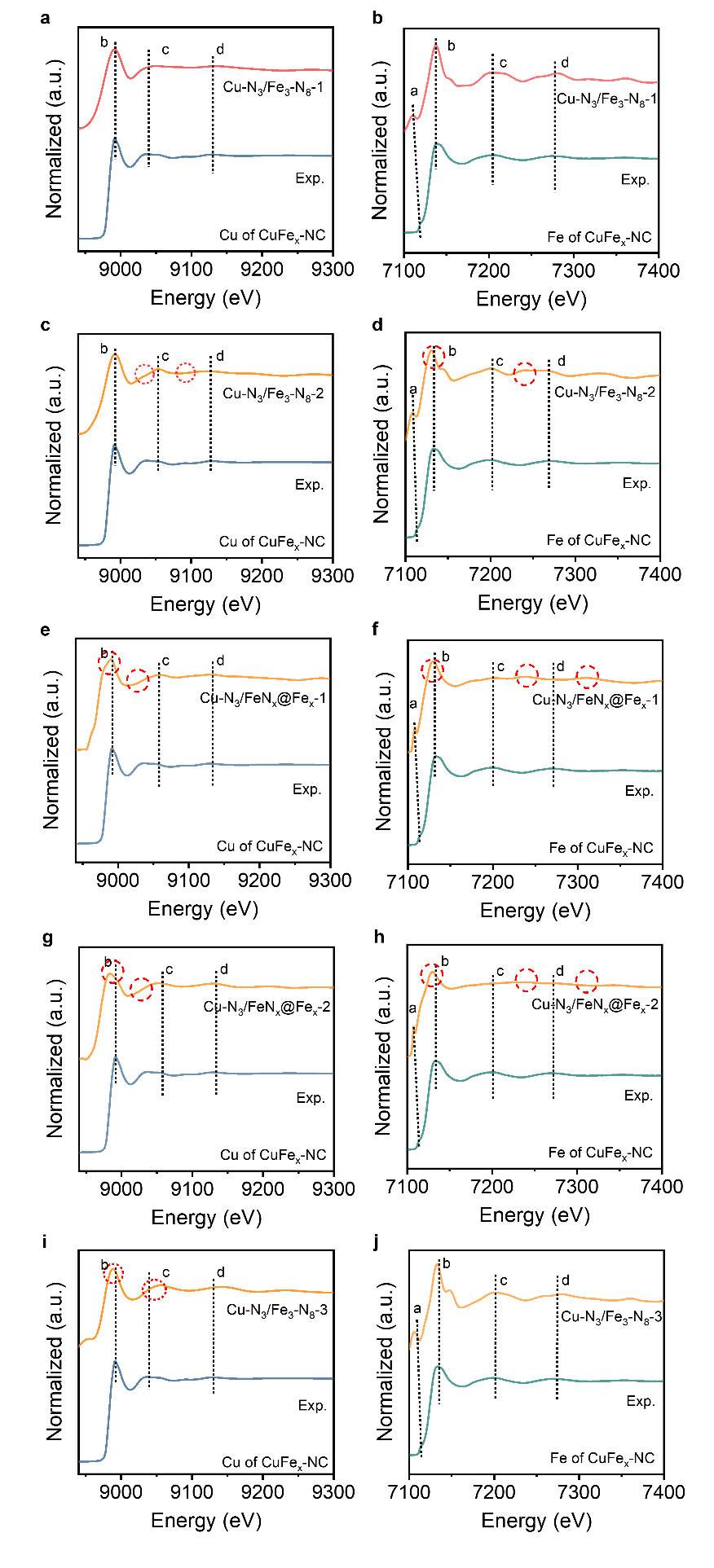


**Figure S****13** (a, b) Comparison between the experimental of Cu K-edge XANES spectrum and Fe K-edge XANES spectrum of Cu-N_3_/Fe_3_-N_8_-1, (c, d) Cu-N_3_/Fe_3_-N_8_-2, (e, f) Cu-N_3_/FeN_x_@Fe_x_-1, (g, h) Cu-N_3_/FeN_x_@Fe_x_-2 and (i, j) Cu-N_3_/Fe_3_-N_8_-3 models.


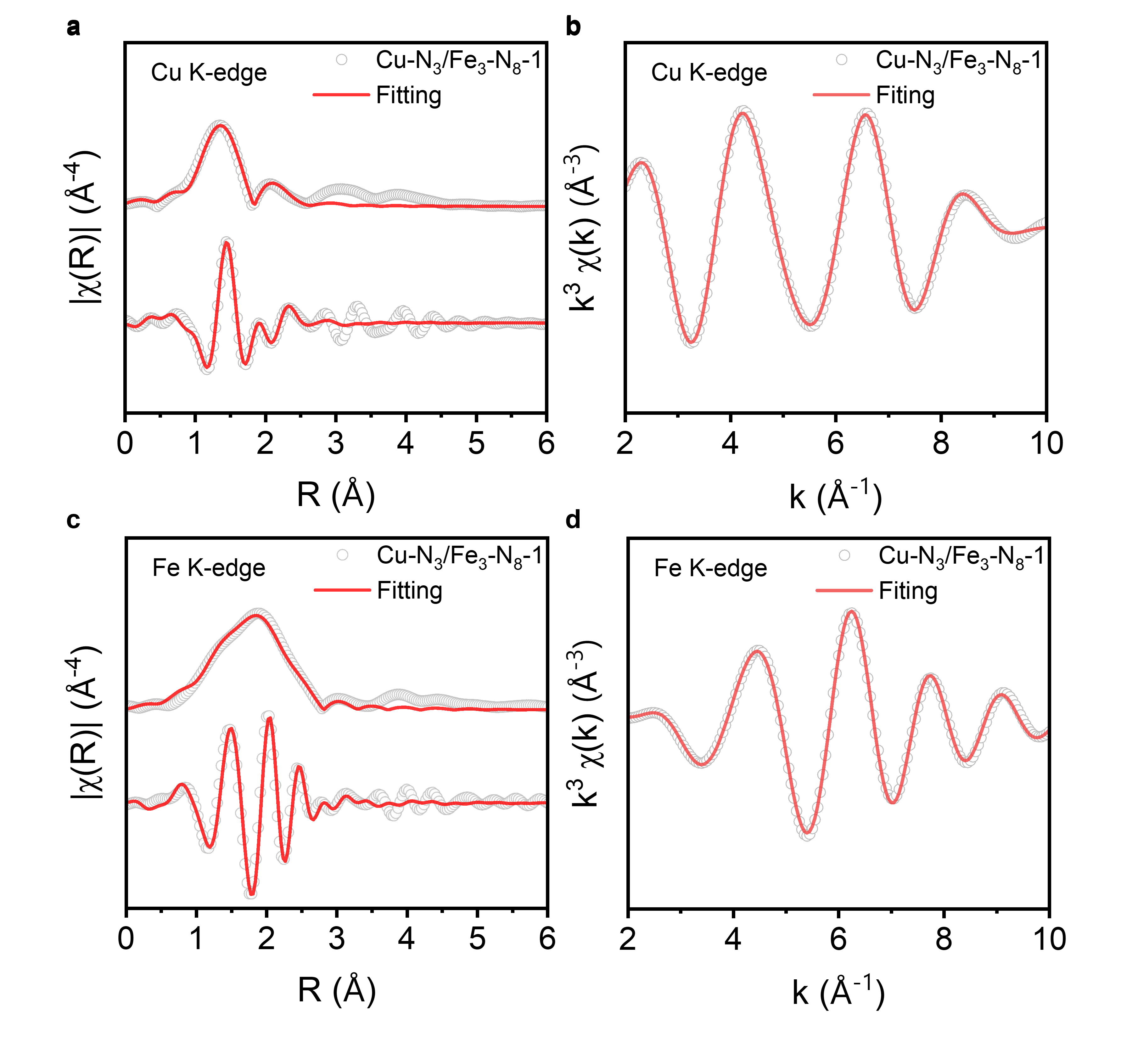


**Figure S14** Comparison between the experimental of EXAFs curves at R space and k space of Cu-N_3_/Fe_3_-N_8_-1.


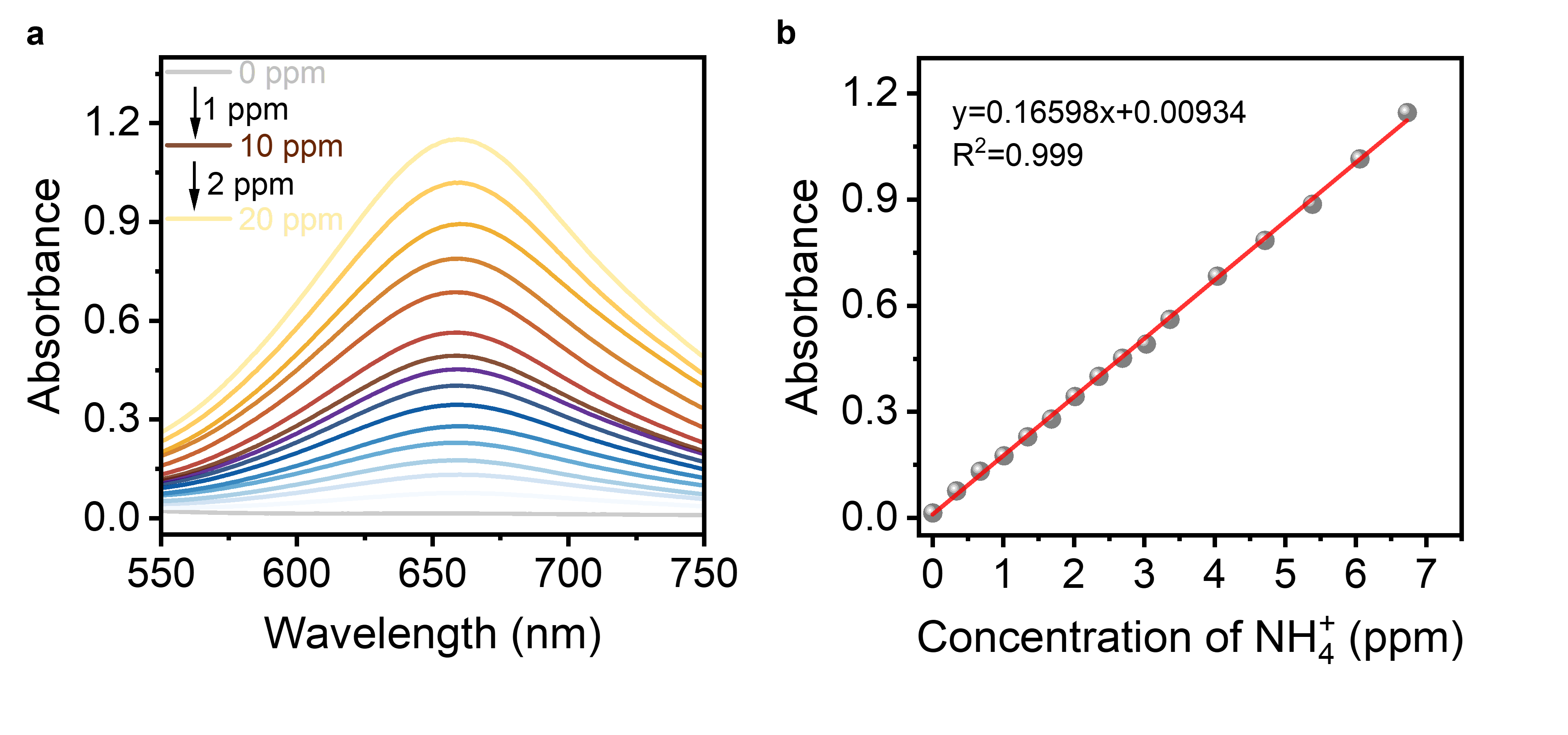


**Figure S15** (a) The UV-vis absorption of various NH_4_Cl concentration. (b) The linear fitting result of NH_4_^+^ calibration curves.


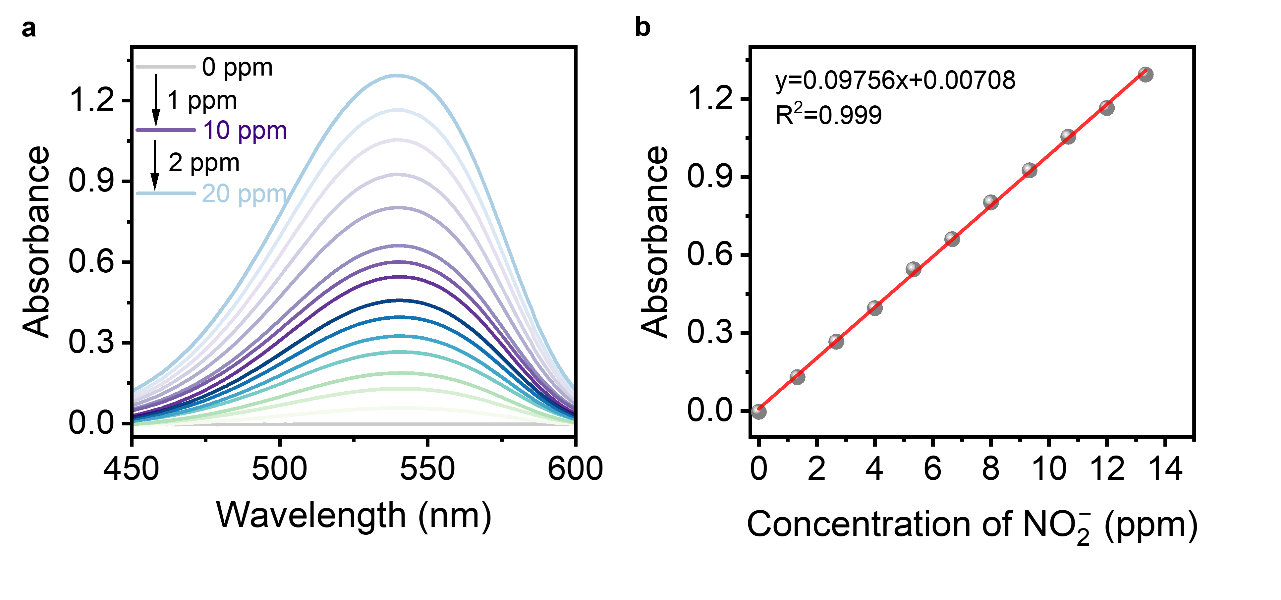


**Figure S16** (a) The UV-vis absorption of various NaNO_2_ concentration. (b) The linear fitting result of NO_2_^‒^ calibration curves.


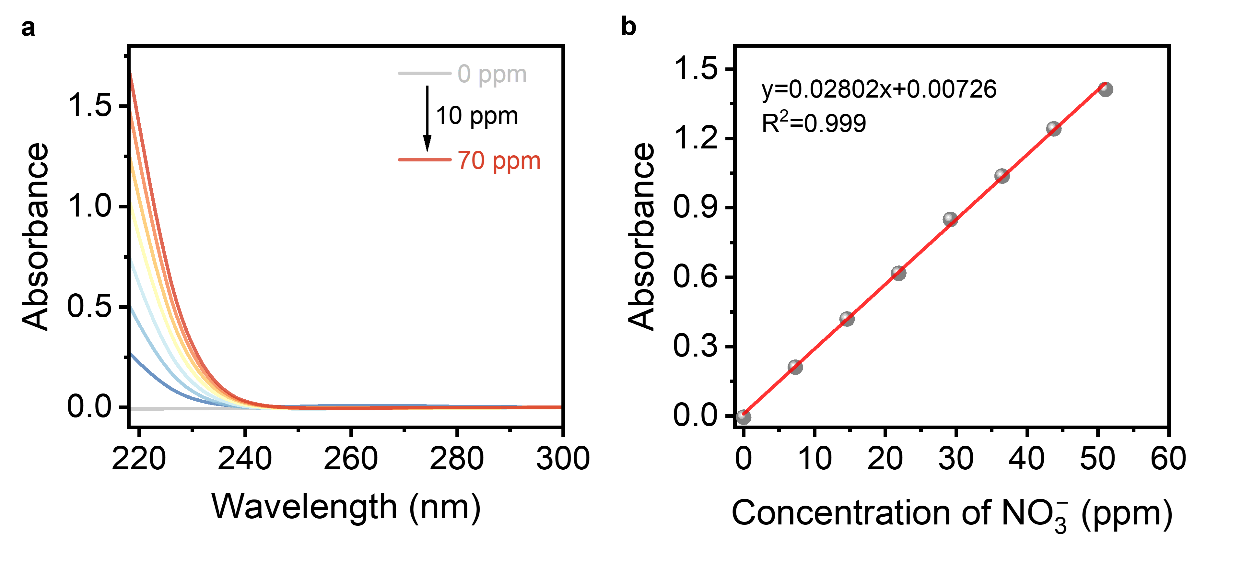


**Figure S17** (a) The UV-vis absorption of various NaNO_3_ concentration. (b) The linear fitting result of NO_3_^‒^ calibration curves.


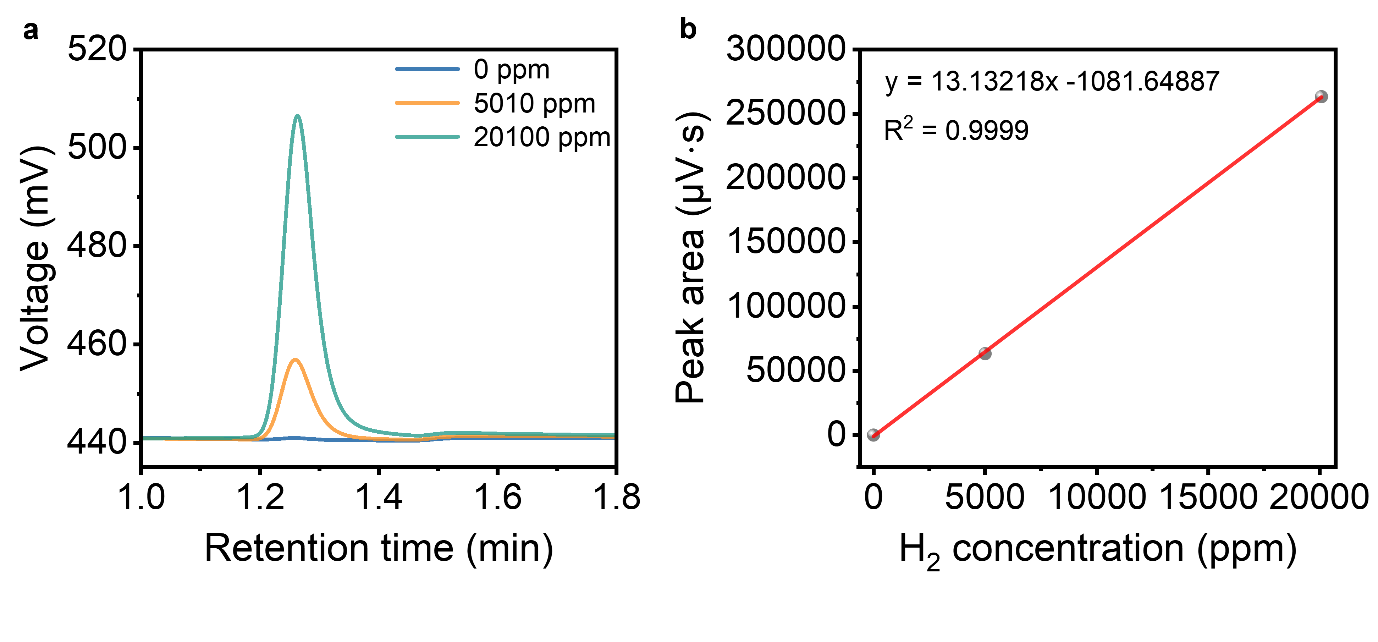


**Figure S18** (a) Gas chromatography results with different H_2_ contents. (b) The linear fitting result of H_2_ calibration curves.


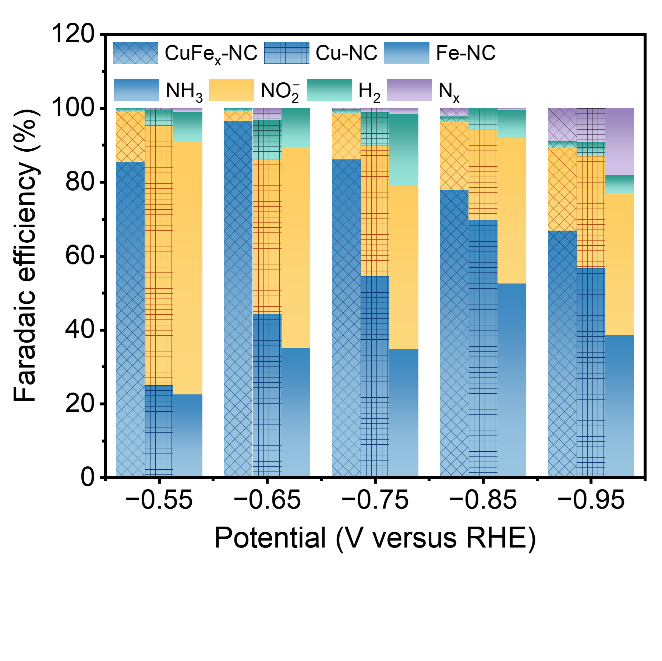


**Figure S19** The Faraday efficiencies of each product for the three catalysts under different reaction potentials (N_x_ refers to nitrogen-containing compounds, primarily consisting of NO and NH_2_OH.).


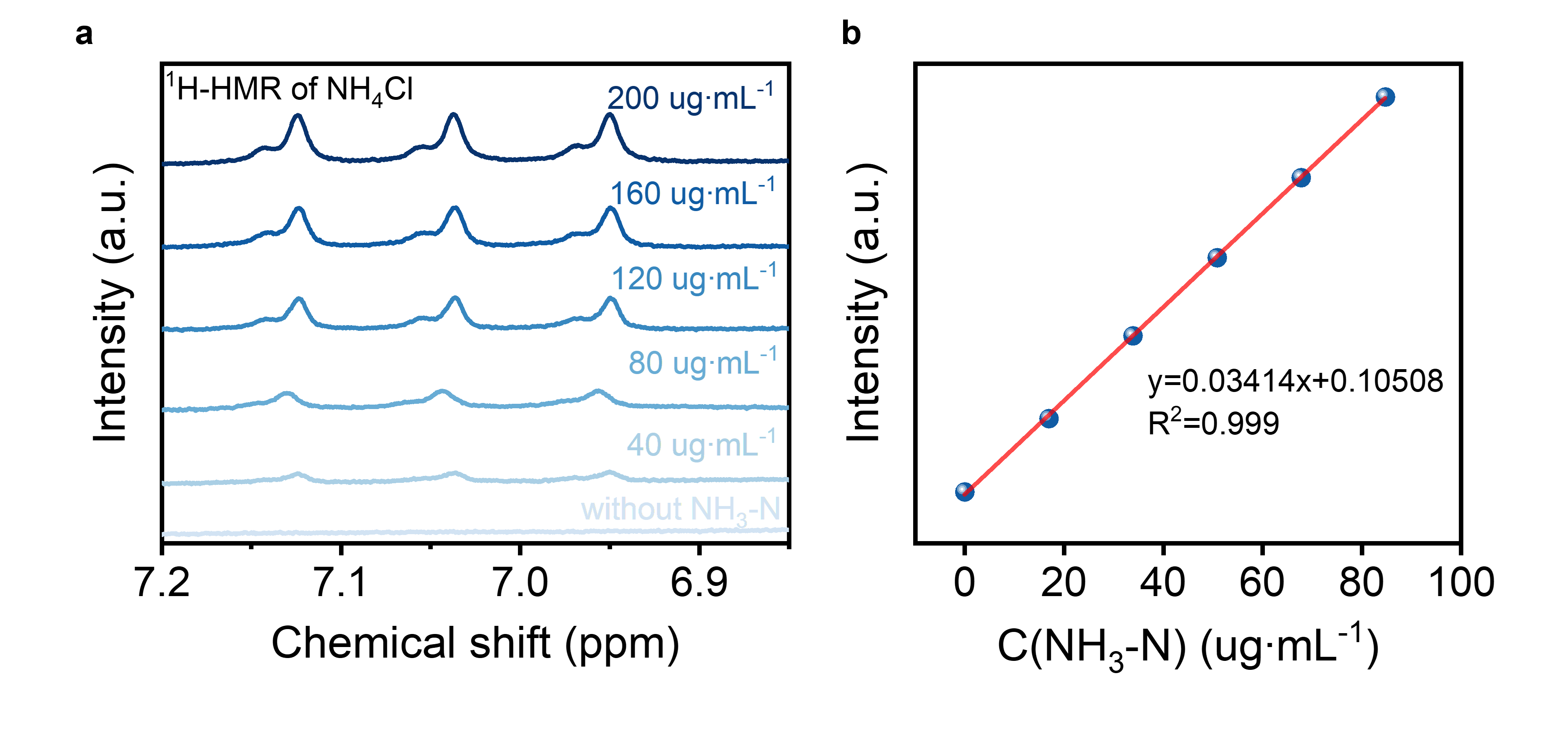


**Figure S20** (a) ^1^H-NMR spectra of NH_4_Cl with different concentrations. (b) The ^1^H-NMR calibration curve for determining NH_3_-N concentration.


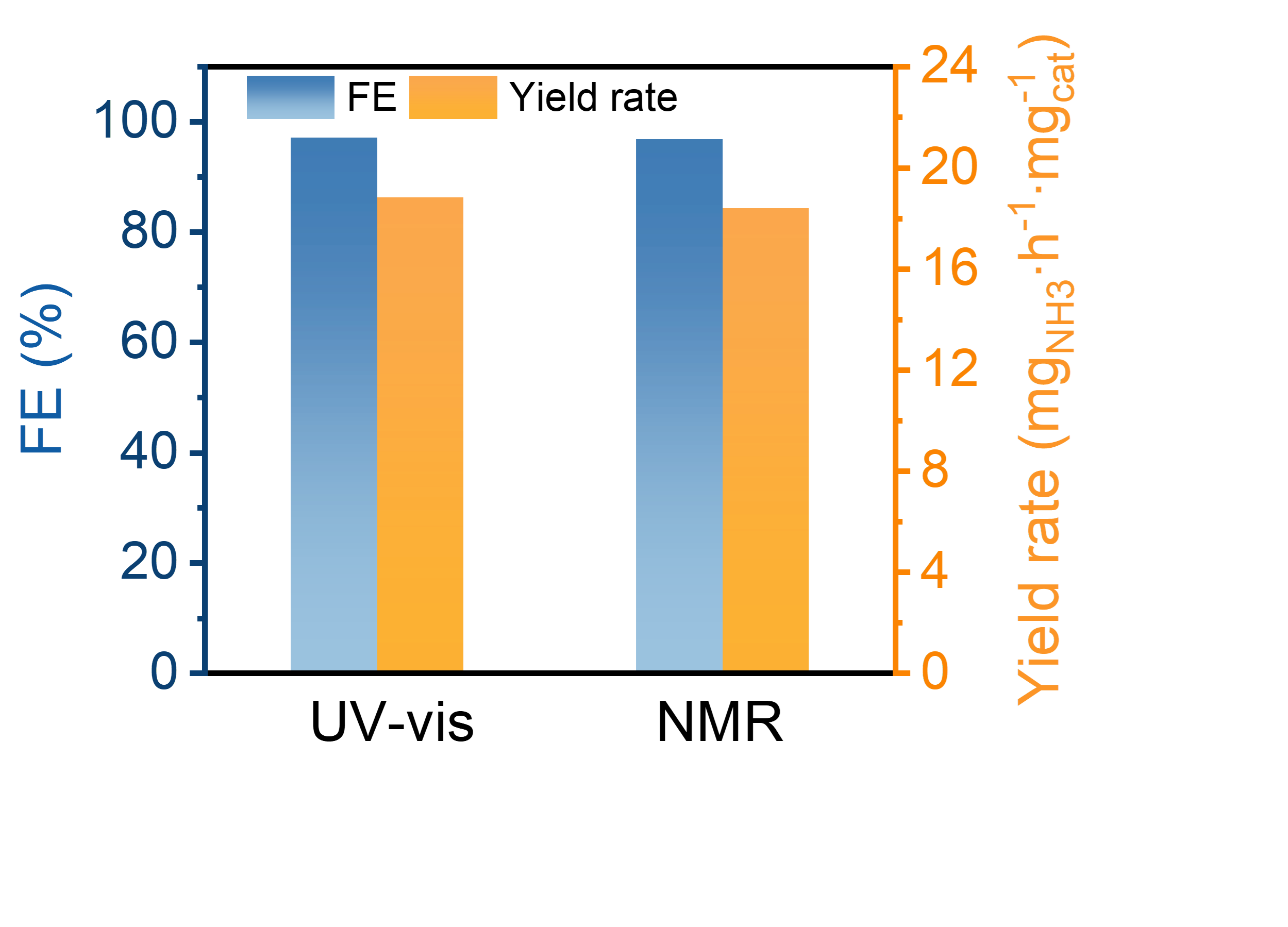


**Figure S21** The FE and The Yield rate of NH_3_ after the NO_3_RR, using ^14^NO_3_^‒^ as the N source.


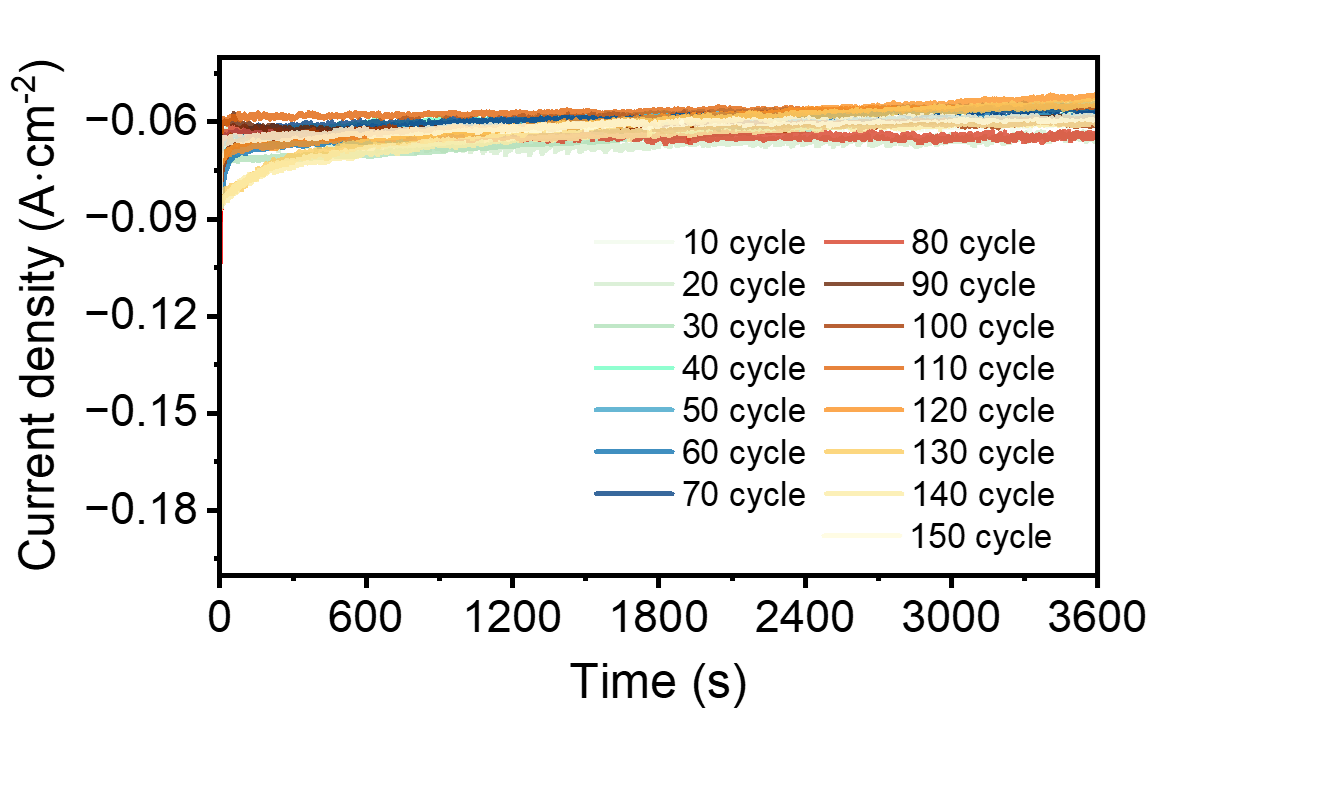


**Figure S22** Current density of CuFe_x_-NC stability test at 0.5M Na_2_SO_4_+0.1M NaNO_3_.


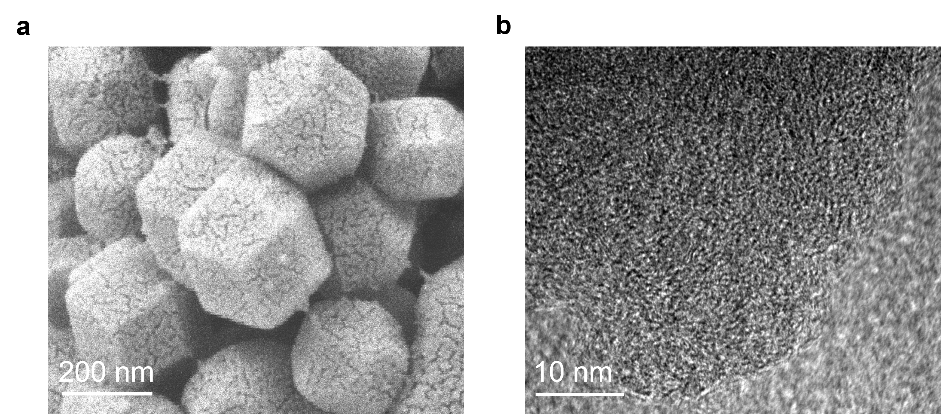


**Figure S23** (a) SEM image and (b) TEM image of the CuFe_x_-NC catalyst following the nitrate reduction reaction.


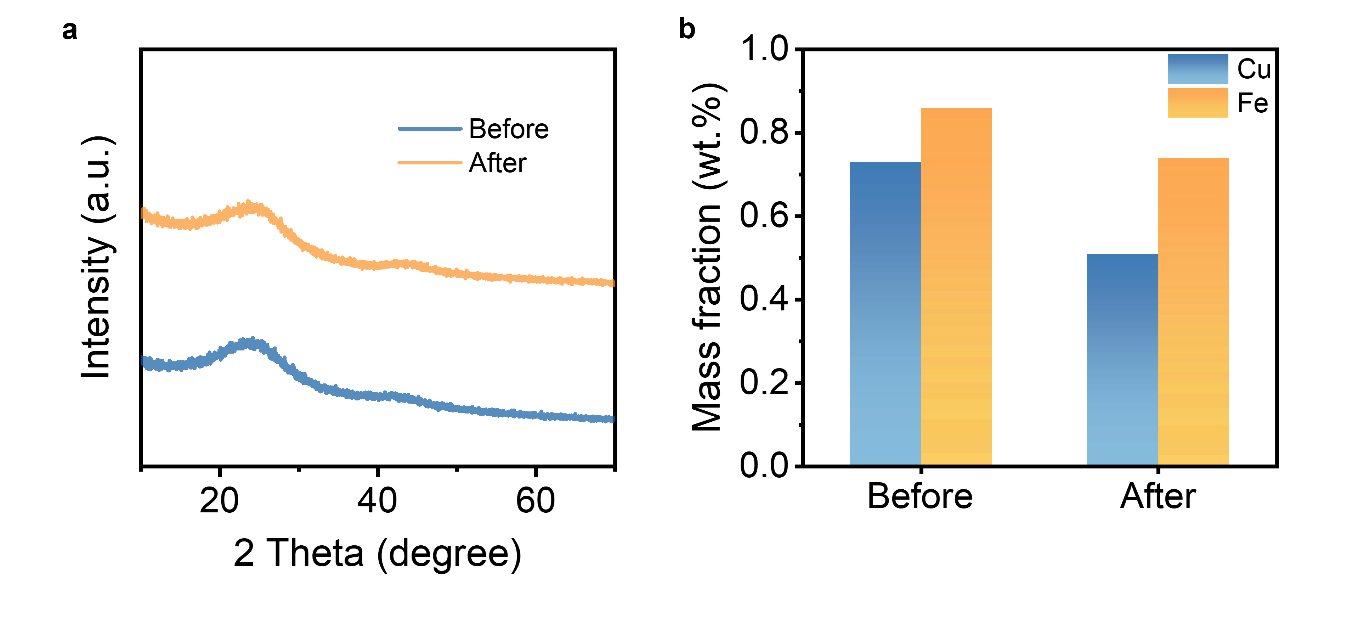


**Figure S24** (a) XRD patterns of CuFe_x_-NC before and after the reaction, (b) The ICP-OES of CuFe_x_-NC before and after the reaction.


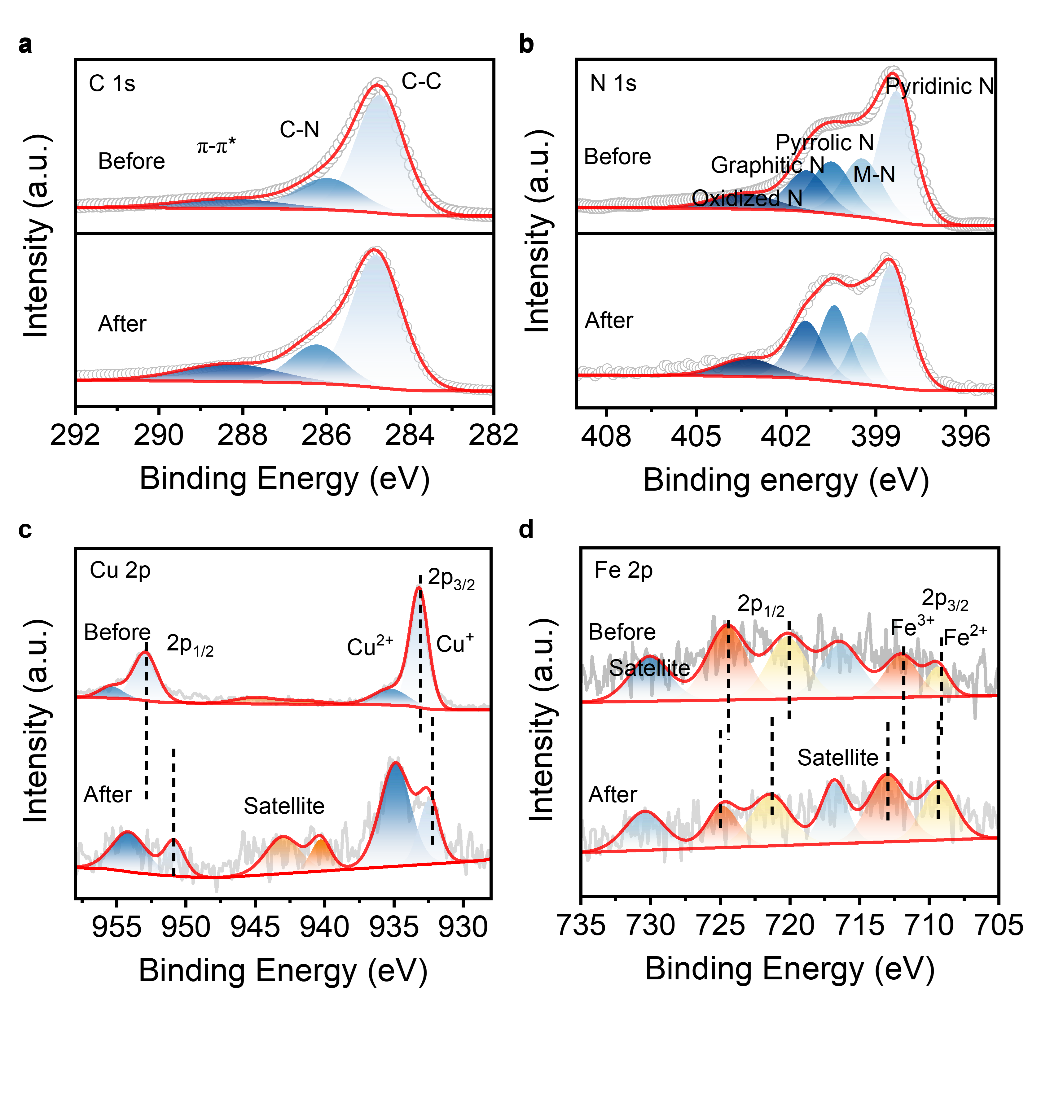


**Figure S25** XPS results before and after the reaction (a) C 1s spectra, (b) N 1s spectra, (c) Cu 2p spectra, (d) Fe 2p spectra.


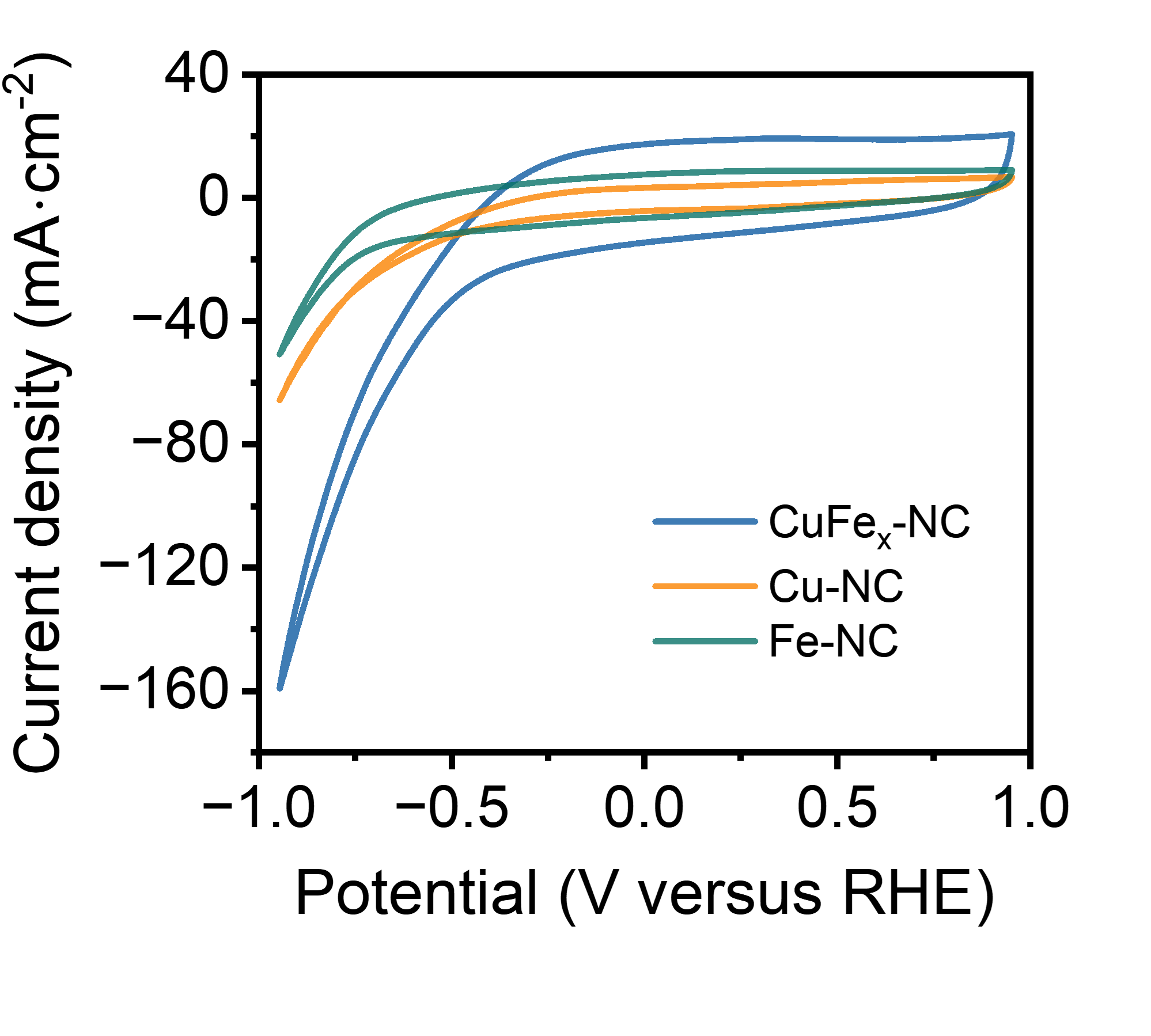


**Figure S26** CV curves of CuFe_x_-NC, Cu-NC, Fe-NC tested in 0.5M Na_2_SO_4_ + 0.1 M NaNO_3_.


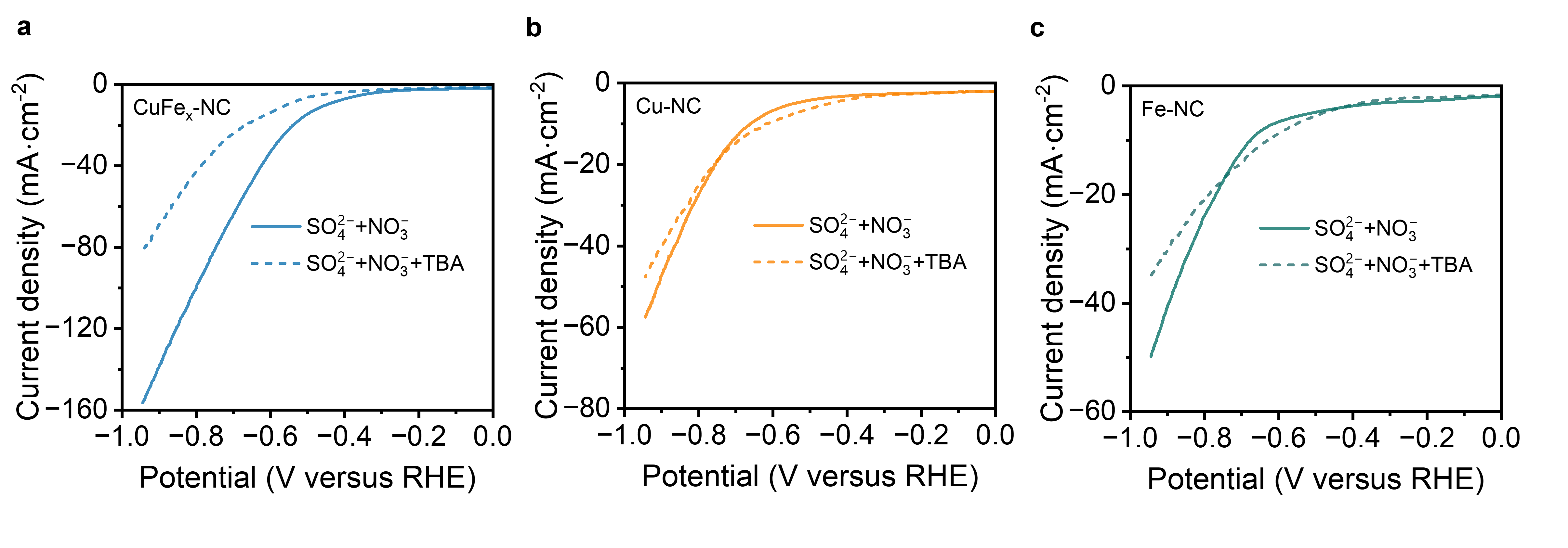


**Figure S27** CV curves of (a) CuFe_x_-NC, (b) Cu-NC and (c) Fe-NC tested in 0.5M Na_2_SO_4_ + 0.1 M NaNO_3_.


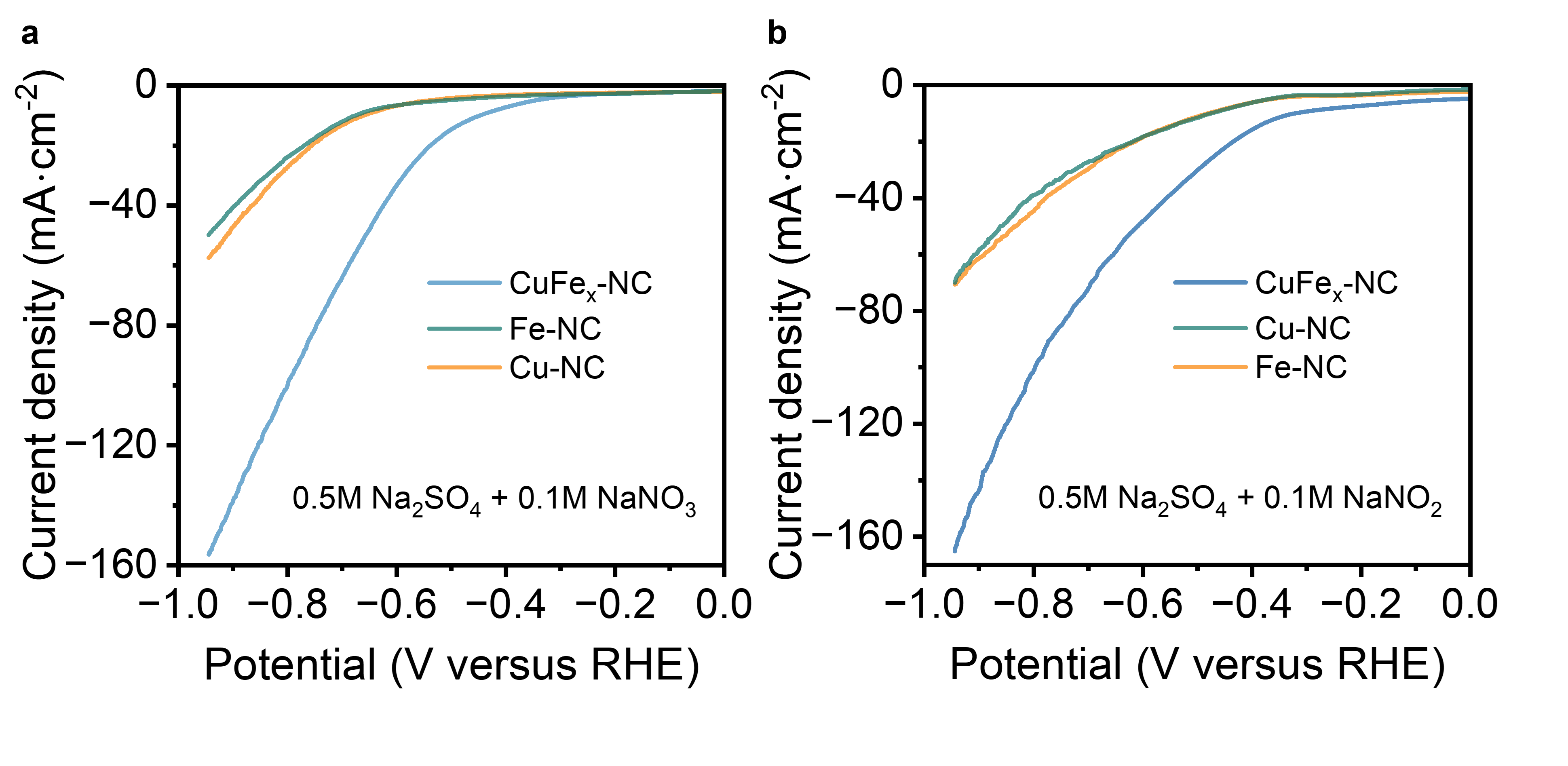


**Figure S28** LSV curves of CuFe_x_-NC, Cu-NC, Fe-NC tested in (a) 0.5M Na_2_SO_4_ + 0.1 M NaNO_3_ and (b) 0.5M Na_2_SO_4_ + 0.1 M NaNO_2_.


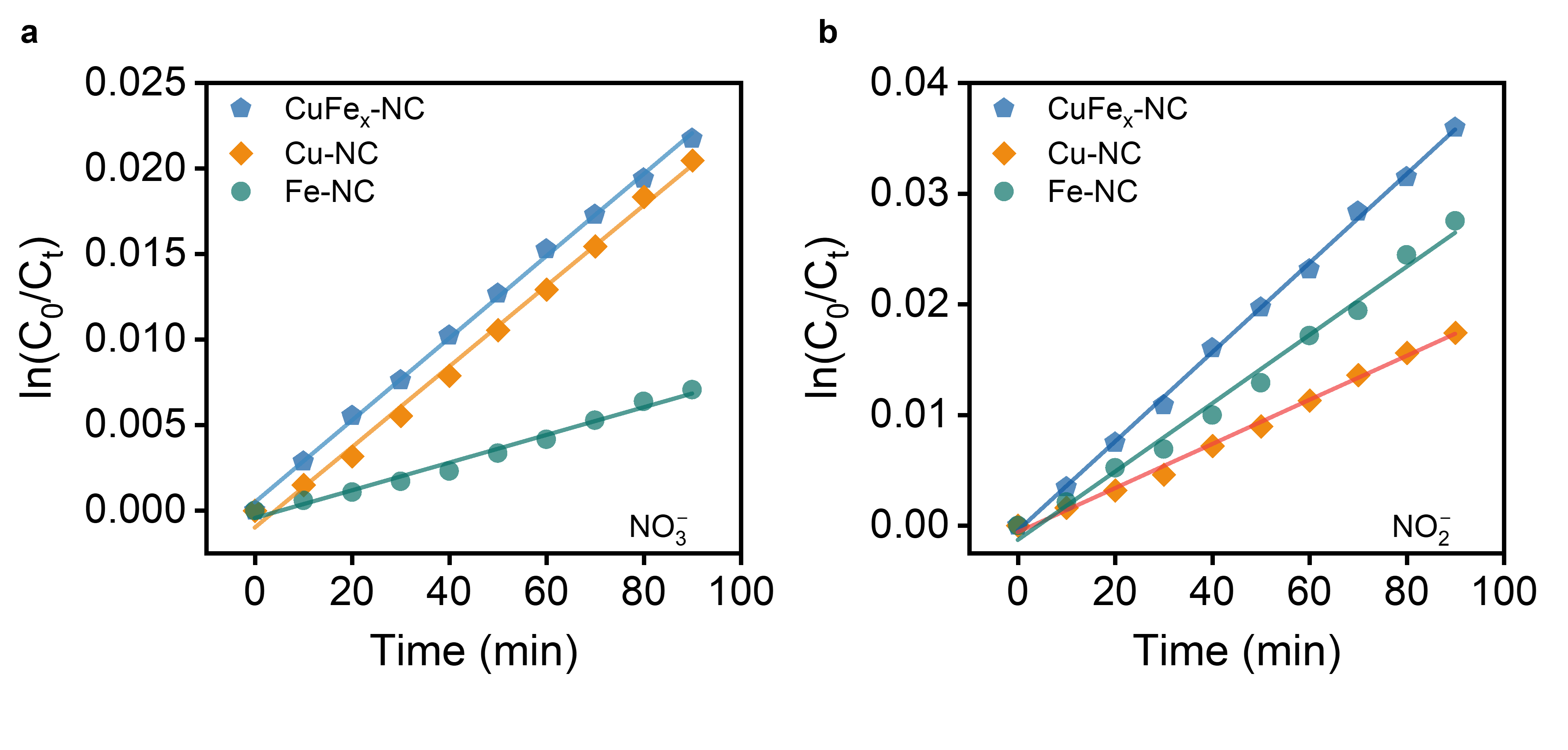


**Figure S29** Time profiles and the first-order kinetic fitting curve for electrochemical reduction (a) NO_3_^⁻^, (b) NO_2_^⁻^.


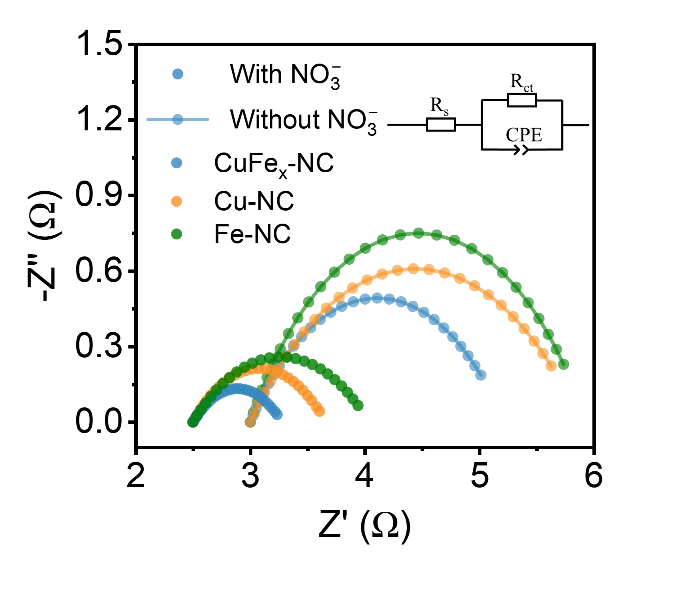


**Figure S30** Electrochemical impedance spectra of CuFe_x_-NC, Cu-NC, and Fe-NC measured in both nitrate-containing and nitrate-free electrolytes.


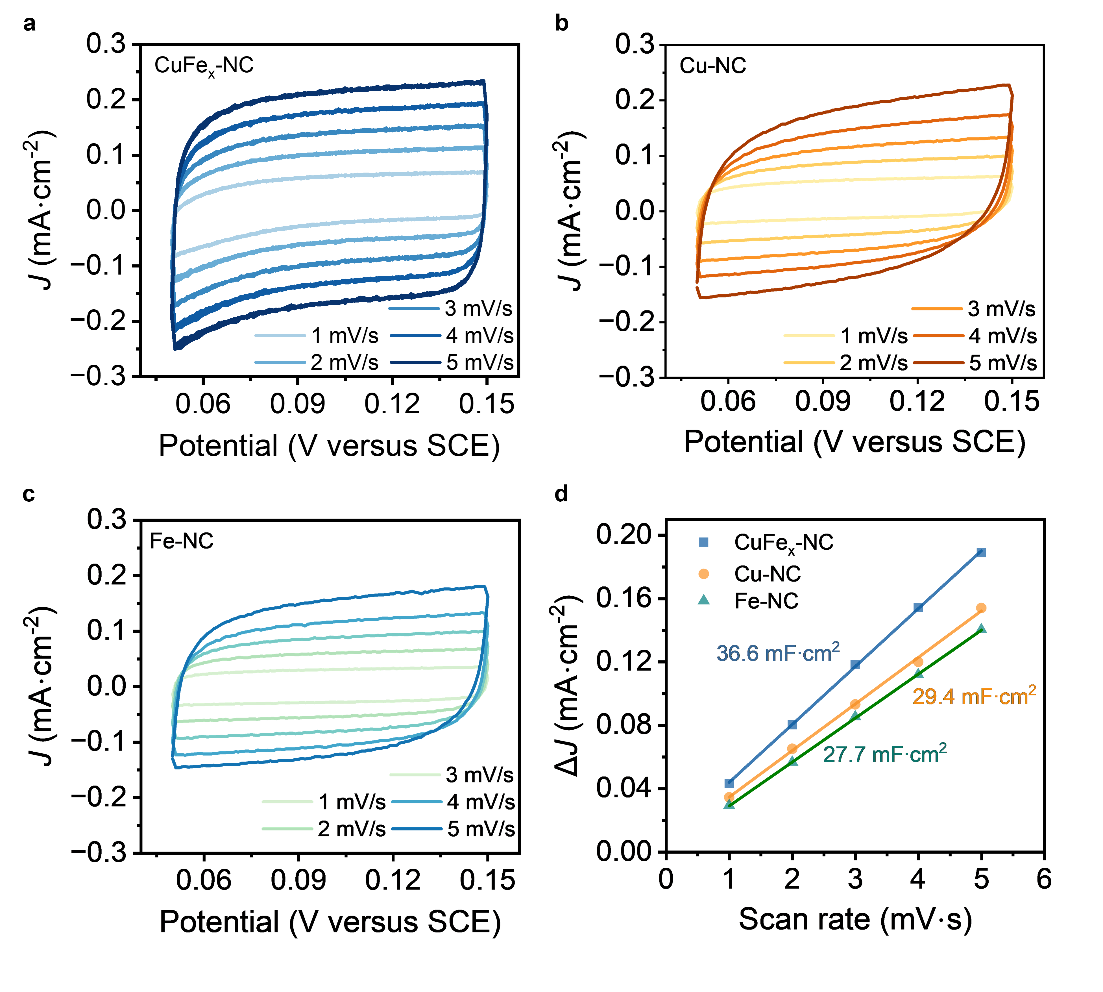


**Figure S31** CV measurements at different scanning rates for (a) CuFe_x_-NC, (b) Cu-NC, and (c) Fe-NC. (d) The calculated ESCA.


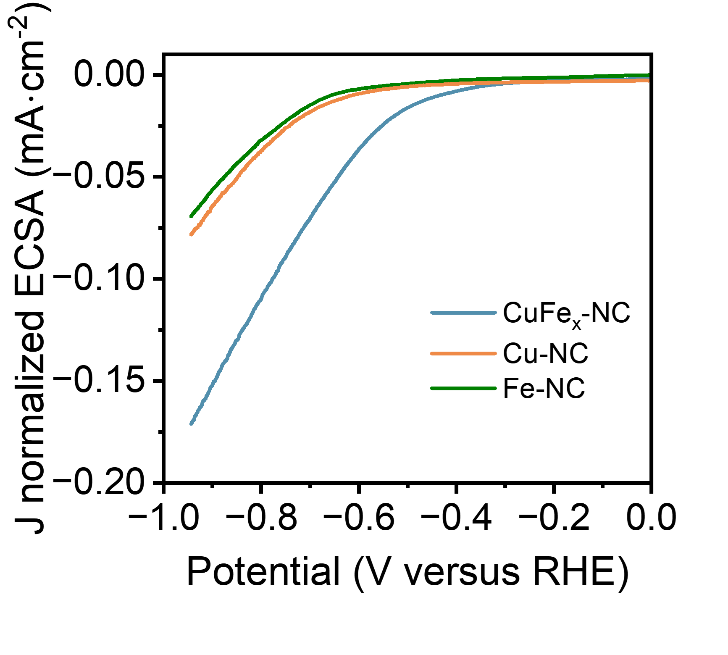


**Figure S32** The normalized LSV curve after ECSA processing.


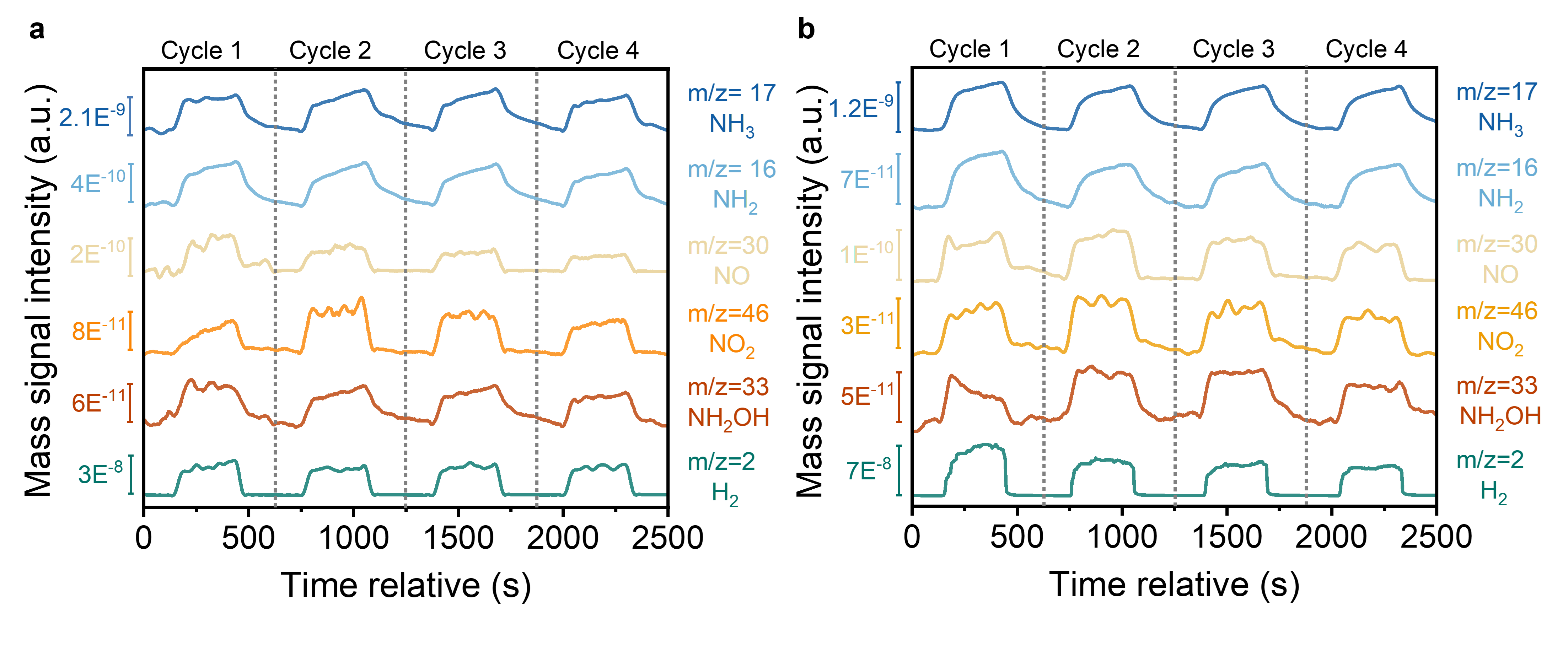


**Figure S33** electrochemical online DEMS of (a) Cu-NC and (b) Fe-NC.


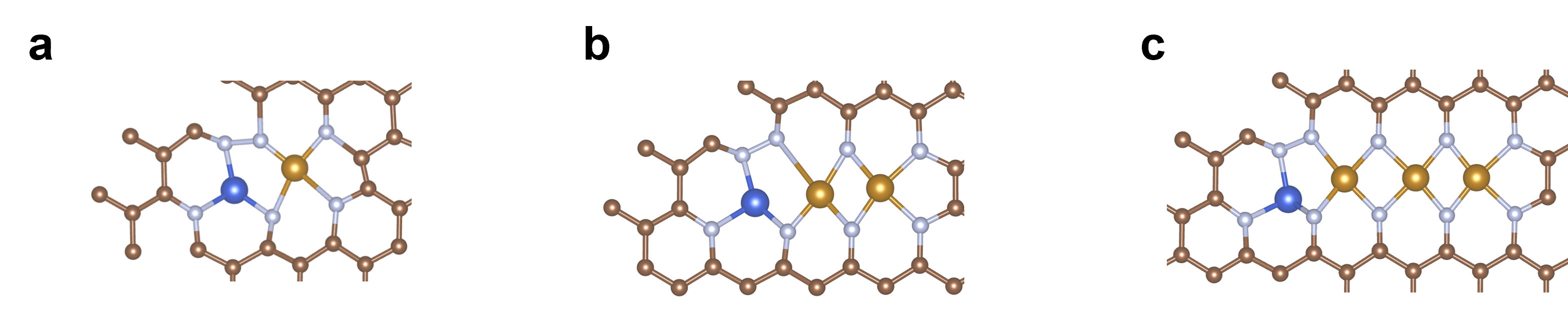


**Figure S34** Models of (a) Cu-N_3_/Fe-N_4_, (b) Cu-N_3_/Fe_2_-N_6_ and (c) Cu-N_3_/Fe_3_-N_8_.


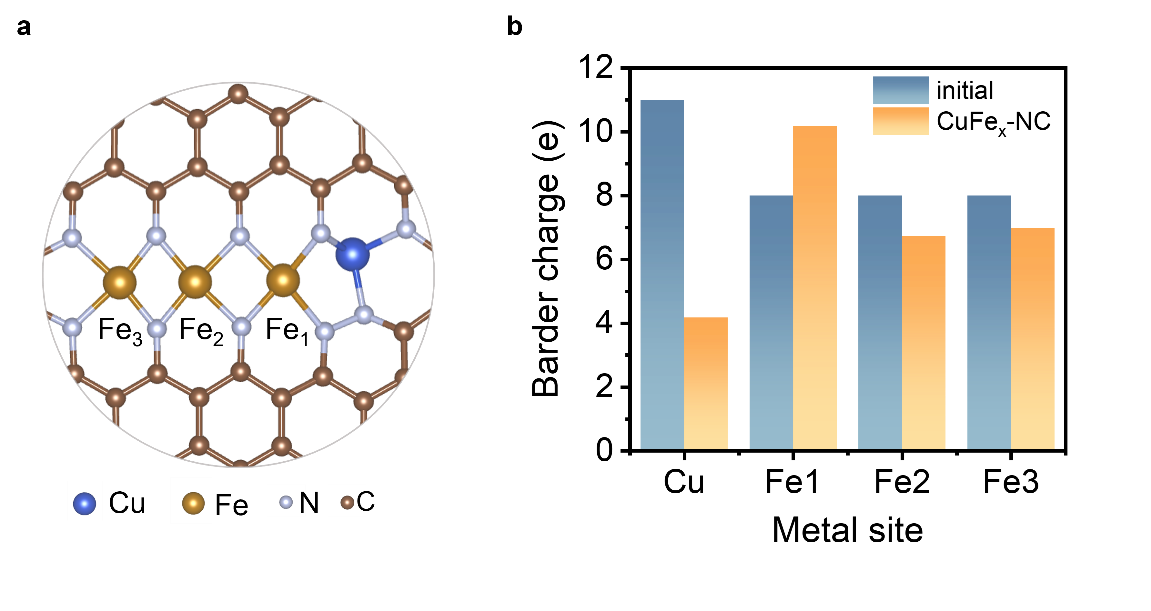


**Figure S35** (a) The distribution of Fe sites in Cu-N_3_/Fe_3_-N_8_, (b) Bader charge analysis of CuFe_x_-NC.


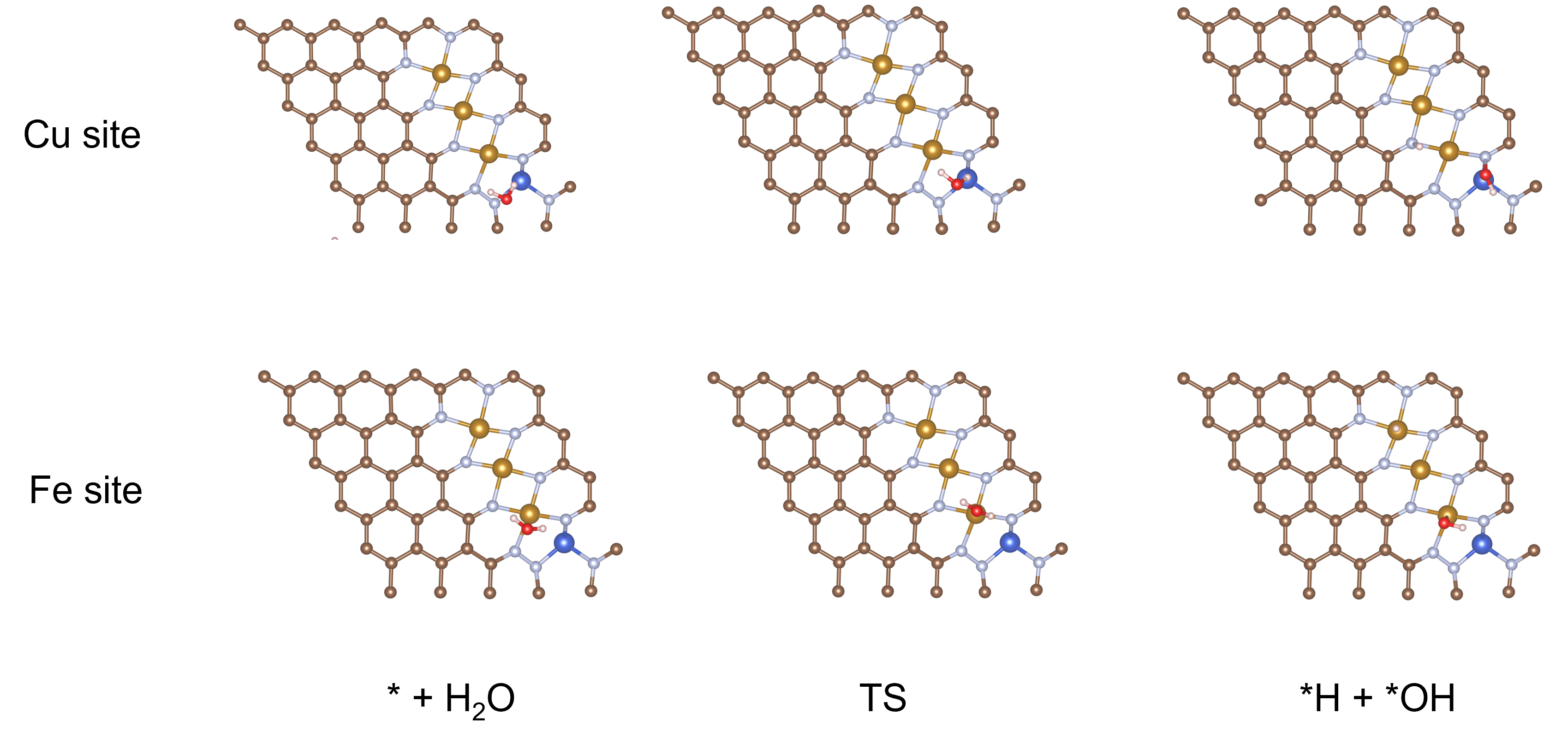


**Figure S36** Structure models diagram of water activation process on Cu site and Fe site of CuFe_x_-NC.


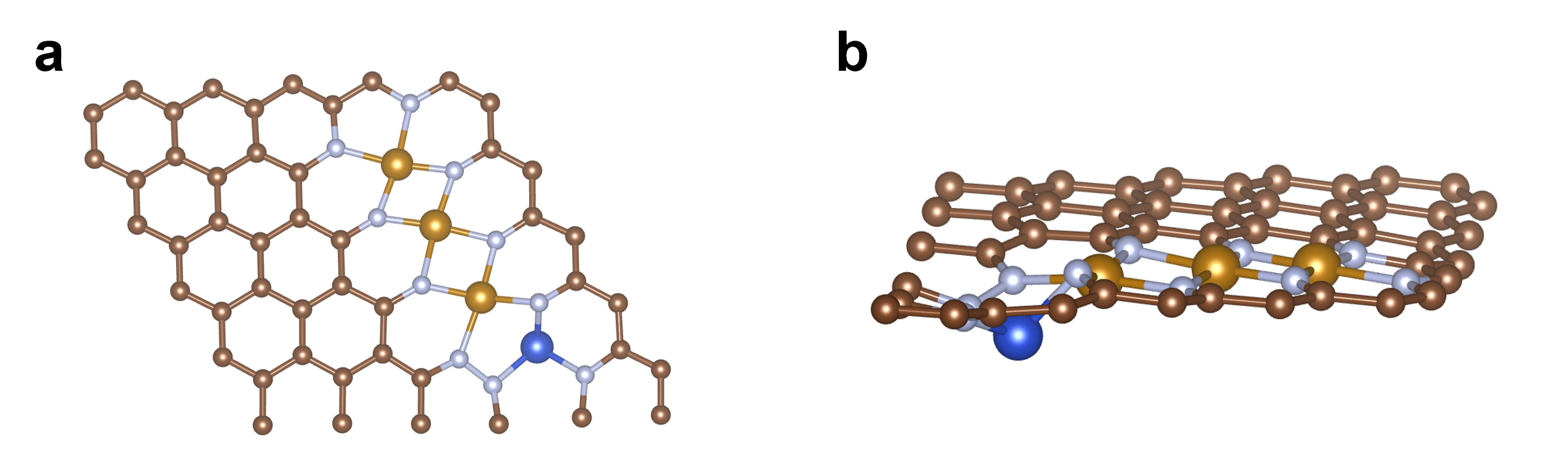


**Figure S37** Slab models of CuFe_x_-NC (a) top view and (b) side view.


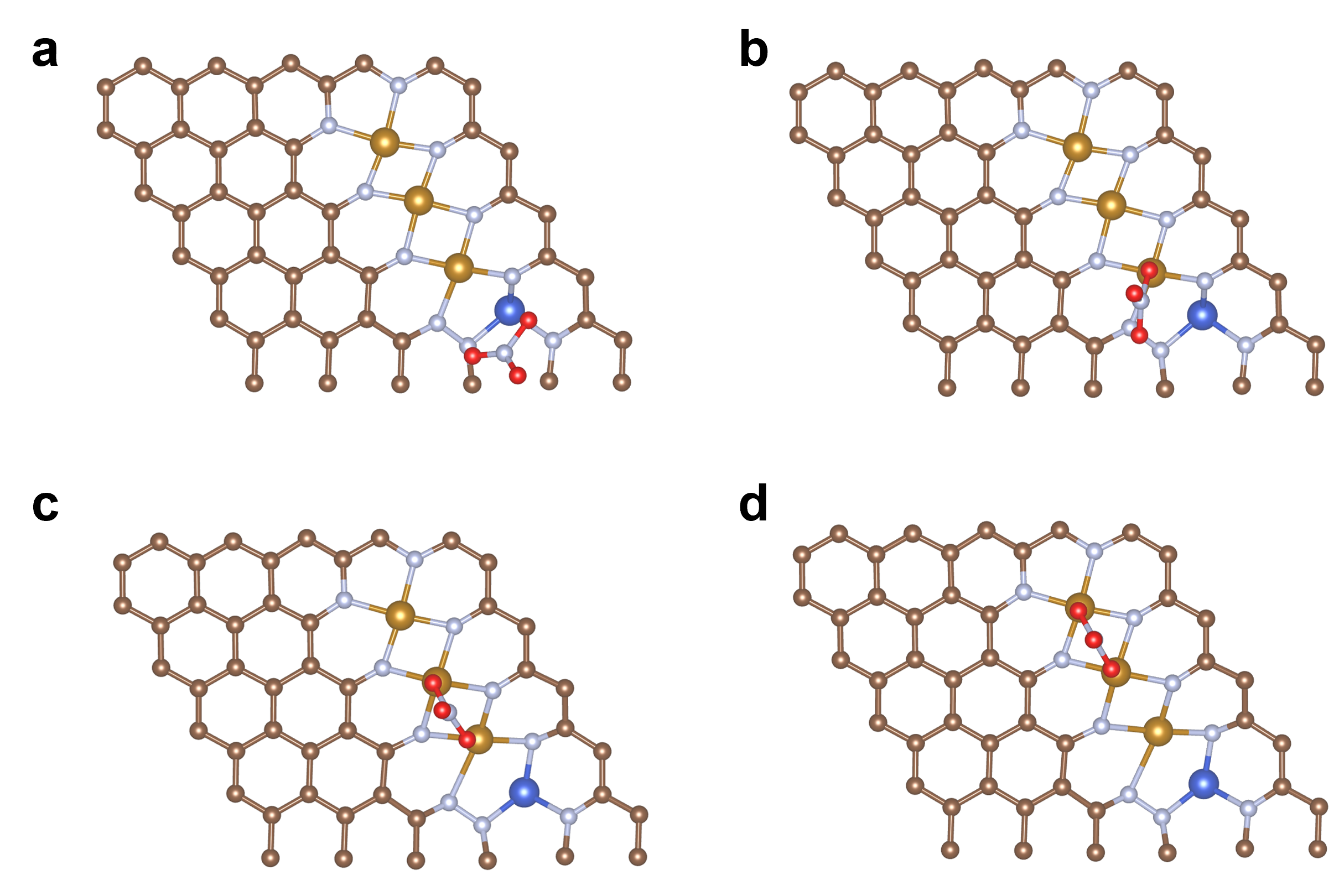


**Figure S38** Adsorption configuration of NO_3_^‒^ at (a) Cu, (b) Fe_1_, (c) Fe_2_ and (d) Fe_3_ sites on CuFe_x_-NC.


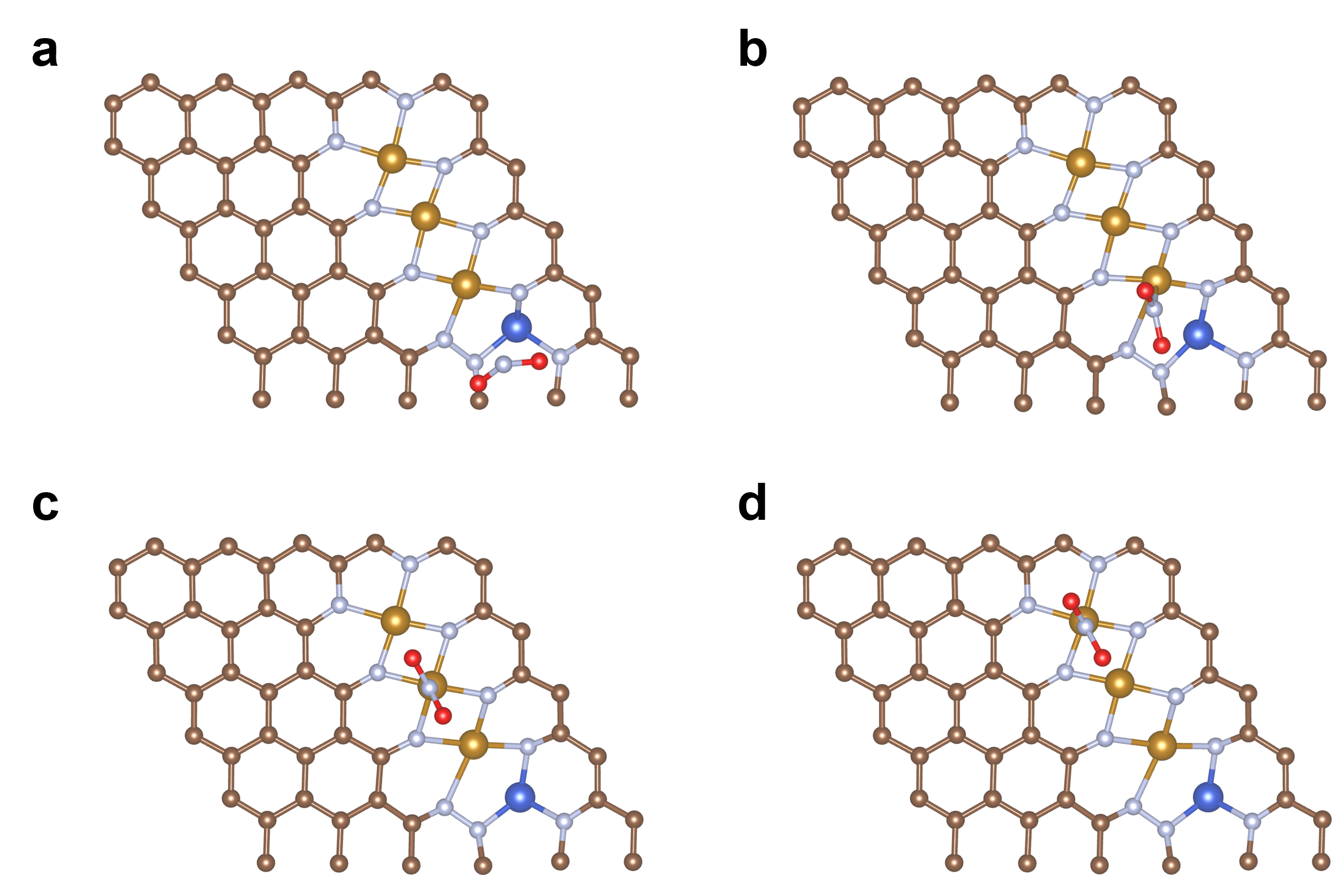


**Figure S39** Adsorption configuration of NO_2_^‒^ at (a) Cu, (b) Fe_1_, (c) Fe_2_ and (d) Fe_3_ sites on CuFe_x_-NC.


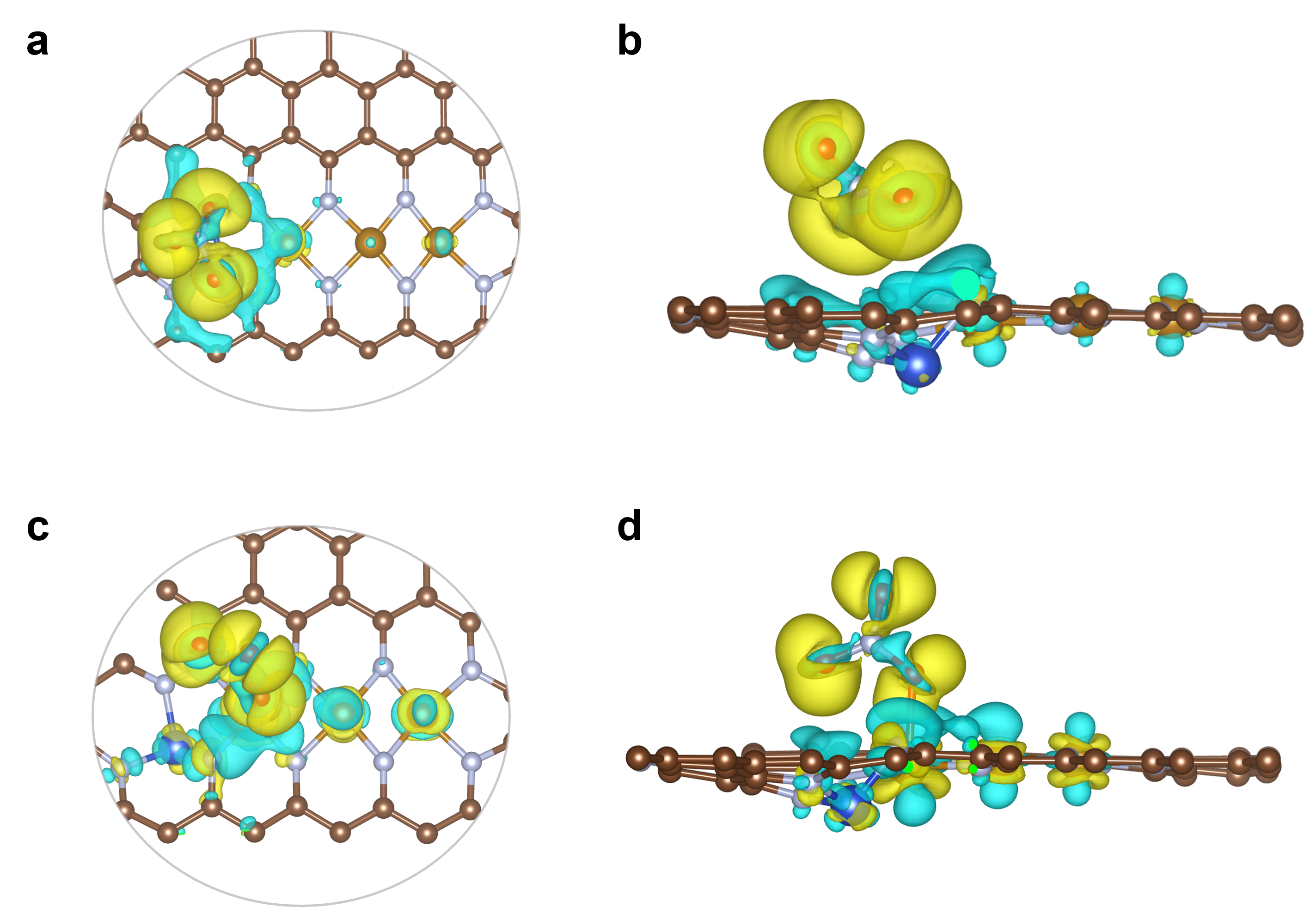


**Figure S40** Charge densities of *NO_3_ adsorber at Cu site of CuFe_x_-NC surface (a) top view, (b) slide view; and charge densities of *NO_3_ adsorber at Fe site of CuFe_x_-NC surface (c) top view, (d) slide view.


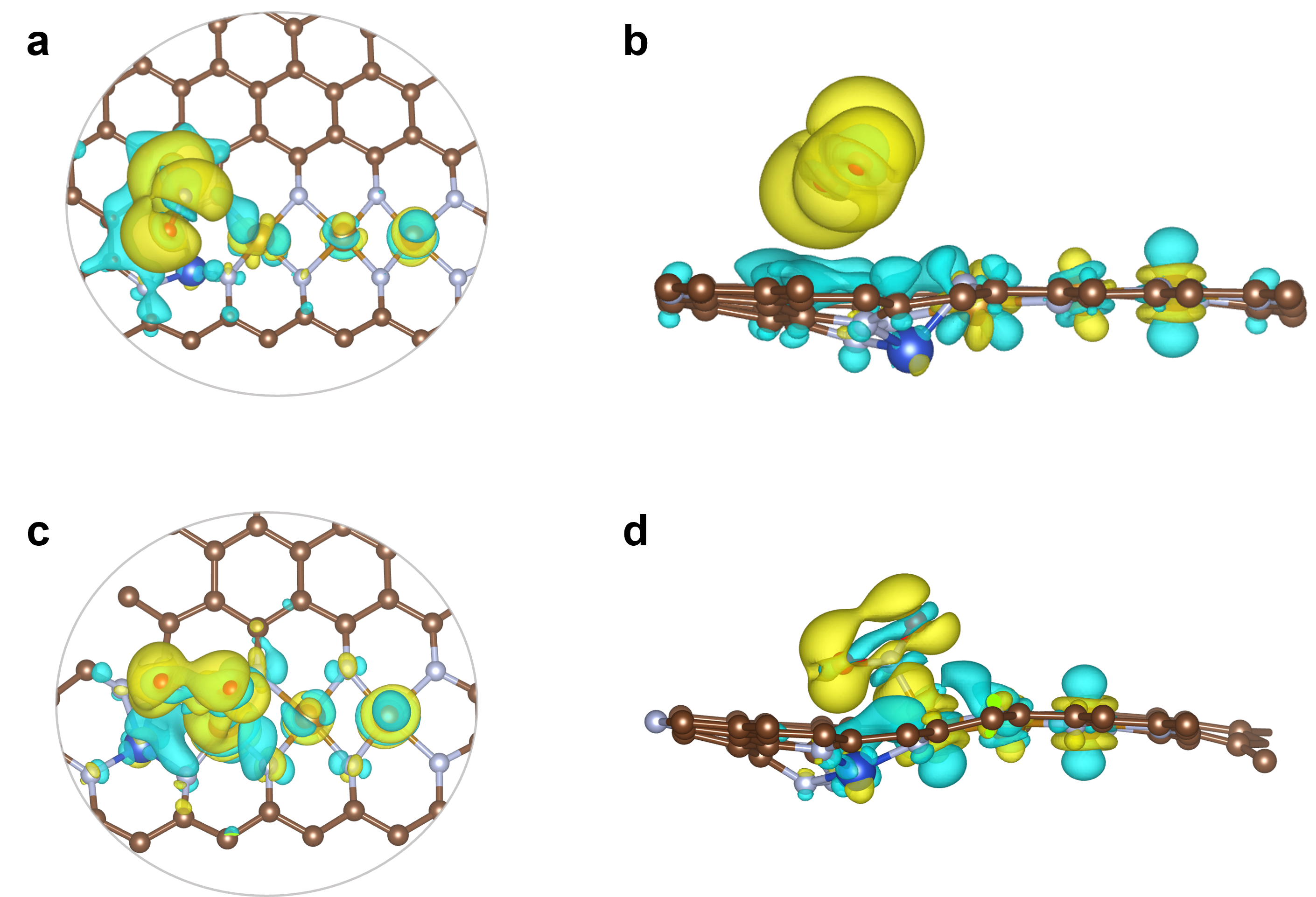


**Figure S41** Charge densities of *NO_2_ adsorber at Cu site of CuFe_x_-NC surface (a) top view, (b) slide view; and charge densities of *NO_2_ adsorber at Fe site of CuFe_x_-NC surface (c) top view, (d) slide view.

**Table S1** N content data of samples collected from XPS.

| Content (at. %)  Sample | Pyridinic N | M-N | Pyrrolic N | Graphitic N | Oxidized N |
| --- | --- | --- | --- | --- | --- |
| CuFe_x_-NC | 7.58797 | 3.81171 | 3.39296 | 2.66475 | 1.59262 |
| Cu-NC | 3.67288 | 1.09516 | 2.29926 | 1.37527 | 1.02743 |
| Fe-NC | 4.01541 | 0.9955 | 1.82557 | 1.37183 | 0.85169 |

**Table S2** EXAFS fitting parameters for Cu-NC and Fe-NC.

| Sample | shell | CN | R(Å) | σ^2^(Å ^-2^) | △E_0_(eV) | R factor |
| --- | --- | --- | --- | --- | --- | --- |
| Cu-NC | Cu-N | 4.0±0.1 | 1.94±0.01 | 0.011 | 1.39±0.07 | 0.0007 |
| Fe-NC | Fe-N | 4.0±0.1 | 1.99±0.01 | 0.013 | 1.59±0.18 | 0.0021 |

CN: coordination numbers; R(Å): bond distance; σ^2^: Debye-Waller factors; △E_0_(eV): the inner potential correction. R factor: goodness of fit.

**Table S3** The Cu K-edge EXAFS fitting parameters for various samples.

| Sample | shell | CN | R(Å) | σ^2^(Å ^-2^) | △E_0_(eV) | R factor |
| --- | --- | --- | --- | --- | --- | --- |
| Cu-N_3_/Fe_3_-N_8_-1 | Cu-N | 3.2±0.1 | 1.95±0.01 | 0.010 | 2.81±0.18 | 0.0017 |
|  | Cu-Fe | 1.0±0.2 | 2.69±0.01 | 0.019 | 6.40±2.78 |  |
| Cu-N_3_/Fe_3_-N_8_-2 | Cu-N | 3.2±0.1 | 1.95±0.01 | 0.006 | 2.64±0.12 | 0.0015 |
|  | Cu-Fe | 0.8±0.1 | 2.71±0.01 | 0.022 | 6.13±1.82 |  |
| Cu-N_3_/FeN_x_@Fe_x_-1 | Cu-N | 3.1±0.1 | 1.95±0.01 | 0.005 | -2.85±0.94 | 0.0048 |
|  | Cu-Fe | 0.8±0.3 | 2.48±0.03 | 0.009 | 2.14±3.46 |  |
| Cu-N_3_/FeN_x_@Fe_x_-2 | Cu-N | 3.0±0.1 | 1.94±0.01 | 0.005 | -2.22±0.26 | 0.0026 |
|  | Cu-Fe | 0.9±0.1 | 2.50±0.01 | 0.009 | -2.34±0.66 |  |
| Cu-N_3_/Fe_3_-N_8_-3 | Cu-N | 3.3±0.1 | 1.95±0.01 | 0.010 | 2.49±0.15 | 0.0022 |
|  | Cu-Fe | 0.9±0.1 | 2.99±0.01 | 0.017 | -9.78±1.19 |  |

**Table S4** The Fe K-edge EXAFS fitting parameters for various samples.

| Sample | shell | CN | R(Å) | σ^2^(Å ^-2^) | △E_0_(eV) | R factor |
| --- | --- | --- | --- | --- | --- | --- |
| Cu-N_3_/Fe_3_-N_8_-1 | Fe-N | 3.8±0.1 | 2.08±0.01 | 0.010 | 6.36±0.67 | 0.0017 |
|  | Fe-Fe | 1.8±0.2 | 2.58±0.01 | 0.009 | 4.24±4.07 |  |
|  | Fe-Cu | 1.3±0.1 | 2.51±0.03 | 0.015 | -5.86±1.34 |  |
| Cu-N_3_/Fe_3_-N_8_-2 | Fe-N | 2.6±0.1 | 2.08±0.01 | 0.006 | 9.17±0.36 | 0.0037 |
|  | Fe-Fe | 2.0±0.2 | 2.57±0.01 | 0.009 | 0.95±0.43 |  |
|  | Fe-Cu | 1.4±0.1 | 2.63±0.09 | 0.033 | 4.30±8.44 |  |
| Cu-N_3_/FeN_x_@Fe_x_-1 | Fe-N | 3.5±0.4 | 2.02±0.02 | 0.010 | 4.34±1.33 | 0.0057 |
|  | Fe-Fe | 2.0±0.1 | 2.63±0.01 | 0.006 | 5.15±1.12 |  |
|  | Fe-Cu | 1.0±0.1 | 2.50±0.04 | 0.001 | 7.97±0.50 |  |
| Cu-N_3_/FeN_x_@Fe_x_-2 | Fe-N | 3.5±0.4 | 2.01±0.01 | 0.010 | 2.28±1.25 | 0.0037 |
|  | Fe-Fe | 2.0±0.1 | 2.64±0.01 | 0.010 | 3.97±1.46 |  |
|  | Fe-Cu | 1.0±0.1 | 2.50±0.01 | 0.004 | -4.01±2.04 |  |
| Cu-N_3_/Fe_3_-N_8_-3 | Fe-N | 3.6±0.1 | 2.07±0.01 | 0.011 | 8.27±0.67 | 0.0024 |
|  | Fe-Fe | 2.3±0.1 | 2.56±0.01 | 0.009 | 3.04±0.41 |  |
|  | Fe-Cu | 0.6±0.3 | 3.18±0.03 | 0.014 | 2.59±3.30 |  |

**Table S5** The content of H_2_ and the Faraday efficiency under different reaction potentials

| Potential (V verse RHE) | Concentration (%) | | | FE (%) | | |
| --- | --- | --- | --- | --- | --- | --- |
|  | CuFe_x_-NC | Cu-NC | Fe-NC | CuFe_x_-NC | Cu-NC | Fe-NC |
| -0.55 | 0.8034 | 1.2754 | 2.1527 | 1.02972 | 4.23269 | 8.09776 |
| -0.65 | 1.1935 | 4.5206 | 3.7199 | 0.78296 | 10.60401 | 10.49573 |
| -0.75 | 2.6353 | 4.5129 | 8.7930 | 1.14255 | 8.84687 | 19.28154 |
| -0.85 | 4.7251 | 3.9996 | 4.7278 | 1.51963 | 5.6488 | 7.65944 |
| -0.95 | 8.8343 | 6.1426 | 9.8516 | 2.03008 | 3.82842 | 4.85503 |

**Table S6** The ICP-OES of CuFe_x_-NC before and after the reaction.

| ICP-OES (wt.%) | initial | After NO_3_RR |
| --- | --- | --- |
| Cu | 0.73 | 0.51 |
| Fe | 0.86 | 0.71 |

**Table S7** Performance comparison with the state-of-the-art catalysts.

| Catalysts | Solution | Potential | FE | Yield | Stability | Ref. |
| --- | --- | --- | --- | --- | --- | --- |
| CuFe_x_-NC | 0.5 M Na_2_SO_4_ and 0.1 M NaNO_3_ | −0.65 V (versus RHE) | 97.1% | 18.83 mg∙h^‒1^∙mg_cat_^‒1^ | 150 h | This work |
| Fe/Cu-HNG | 1 M KOH and 0.1 M KNO_3_ | −0.3 V (versus RHE) | 92.51% | 1.08 mmol∙h^–1^∙mg^–1^ (at -0.5 V versus RHE) | 24 h | ^[11]^ |
| Bi_1_-CuCo_2_O_4_ | 0.5 M Na_2_SO_4_ and 0.1 M KNO_3_ | −0.8 V (versus RHE) | 95.53% | 448.74 µmol∙h^−1^∙cm^−2^ | 12 h | ^[12]^ |
| Cu-N-C | 0.5 M Na_2_SO_4_ and 50 mg∙L^–1^NaNO_3_ | −1.5 V (versus SHE) | 94% | 9.23 mg∙h^−1^∙mg^−1^ | 8 h | ^[13],[14]^ |
| Cu-NC | 0.2 M Na_2_SO_4_ and 0.2 M NaNO_3_ | −0.89 V (versus RHE) | 82.1% | 4731.0 μg∙h^−1^∙mg^-1^ (at -1.09 V versus RHE) | 20 h | ^[15],[14]^ |
| Cu_1_-N_4_ | 1 M KOH and 0.1 M KNO_3_ | −1 V (versus RHE) | 95.5 % | 13.8 mol∙g^−1^∙h^−1^ | 20 h | ^[16],[14]^ |
| Cu@Th-BPYDC | 1 M KOH and 0.1 M KNO_3_ | −0 V (versus RHE) | 92.6% | 225.3 μmol∙h^–1^∙cm^–2^ | 1000 CV | ^[17],[14]^ |
| **1**-Cu | 0.5 M Na_2_SO_4_ and 5 mM NaNO_3_ | −0.9 V (versus RHE) | 85.5% | 66 μmol∙h^–1^∙cm^–2^ | 12 h | ^[18],[14]^ |
| Cu-N-C SAC | 0.1 M KOH and 0.1 M KNO_3_ | −1 V (versus RHE) | 84.7 % | 4.5 mg∙cm^–2^∙h^–1^ | 20 h | ^[19],[14]^ |
| O-Cu–PTCDA | 0.1 M PBS and 500 ppm KNO_3_ | −0.4 V (versus RHE) | 85.9 % | 436 ± 85 μg∙h^−1^∙cm^−2^ | 4 h | ^[20],[14]^ |
| Cu-*cis*-N_2_O_2_ | 0.5 M Na_2_SO_4_ and 1000 ppm KNO_3_ | −1.6 V (versus RHE) | 93.19% | 27.84 mg∙h^−1^∙cm^−2^ | 2000 h | ^[21],[14]^ |
| Cu(I)-N_3_C_1_ | 0.05 M Na_2_SO_4_ and 100 mg∙L^-1^ NaNO_3_ | −0.64 V (versus RHE) | 94.8% | 5466 mmol∙g^–1^∙h^–1^ | 14 h | ^[22],[14]^ |
| Cu/Ni-NC | 0.05 M Na_2_SO_4_ and 100 ppm NaNO_3_ | −0.7 V (versus RHE) | 97.28% | 5480 mg∙h^−1^∙mg^−1^ | 20 h | ^[23],[14]^ |
| BCN-Cu | 1 M KOH and 0.1 M KNO_3_ | −0.5 V (versus RHE) | 98.23% | 1900.07 μg∙h^−1^∙cm^−2^ | 16 h | ^[24],[14]^ |
| BCN@Cu/BCN | 0.1 M KOH and 0.1 M KNO_3_ | −0.6 V (versus RHE) | 95.32% | 172226.5 μg∙h^−1^∙mg^−1^ | 10 h | ^[25],[14]^ |
| Cu_3_N | 0.1 M Na_2_SO_4_ and 0.1 M KNO_3_ | −0.6 V (versus RHE) | 93.1% | 2.9 mg∙cm^2^∙h^−1^ | 17 h | ^[26]^ |
| TTA-TPH-CuCo | 0.5 M K_2_SO_4_ and 0.1 M KNO_3_ | −0.65 V (versus RHE) | 90.48% | 14.22 mg·h^−1^·cm^−2^ | 50 h | ^[27]^ |
| RuFe nanoflowers | 0.5 M Na_2_SO_4_ and 0.1 M NaNO_3_ | −0.3 V (versus RHE) | 92.9% | 38.68 mg∙h^‒1^∙mg_cat_^‒1^ (−0.65 V versus RHE) | 20 h | ^[28]^ |
| Cu_1_Co_1_-HDS | 1 M KOH and 0.5 M KNO_3_ | −0.5 V (versus RHE) | 96.15% | 4.625 mmol·h^-1^·cm^-2^ | 20 h | ^[29]^ |
| Zn_5_-NiS_4_TP MOF | 0.05 M K_2_SO_4_ and 0.5 M KNO_3_ | −1.2 V (versus RHE) | 92.57% | 23650.63 µg·h^−1^·mg_cat_^−1^ | 20 h | ^[30]^ |
| NiTP-CoTAPP | 0.5 M KNO_3_ | −0.8 V (versus RHE) | 85.6% | 160.2 mmol·h^−1^·g^−1^ | 8 h | ^[31]^ |
| RuCu DAs/NGA | 0.1 M KOH and 0.1 M KNO_3_ | −0.4 V (versus RHE) | 95.7% | 3.1 mg·h^−1^·cm^−2^ | 10 h | ^[32]^ |
| Cu-CoP | 0.1 M K_2_SO_4_ and 0.01 M KNO_3_ | −1 V (versus RHE) | 85.1% | 7.65 mg·h^−1^·cm^−2^ | 80 h | ^[33]^ |
| Fe@Cu_1_FeO_x_ | 0.1 M K_2_SO_4_ and 50 mg·L^–1^ KNO_3_ | −1.3 V (versus SCE) | 95.4% | 1.98 mg·h^−1^·cm^−2^ | 60 h | ^[34]^ |
| ImPy-COF-Mn | 1 M KOH and 2 mg·L^–1^ KNO_3_ | −0.7 V (versus RHE) | 95.64% | 1927 mmol·h^−1^·g^−1^ | 14 h | ^[35]^ |
| Cu-Fe-N-C | 1 M KOH and 0.1 M KNO_3_ | −0.8 V (versus RHE) | 95.08% | 1.22 mmol·h^−1^·cm^−2^ | 100 h | ^[36]^ |
| Si/I-NiO@CC | 0.05 M H_2_SO_4_ and 0.5 M KNO_3_ | −0.7 V (versus RHE) | 96.8% | 10.6 mg·h^−1^·cm^−2^ | 420 h | ^[37]^ |
| NiCo-DSAC/MXene | 1 M KOH and 0.2M KNO_3_ | −0.5 V (versus RHE) | 92.46% | 8.46 mg·h^−1^·cm^−2^ | 10 h | ^[38]^ |
| Cu_1_/WO_3_ | 50.0 mM Na_2_SO_4_ and 22.5 mg·L^−1^ | −0.6 V (versus RHE) | 93.7% | 1274.4 mg·h^−1^·g^−1^ | 2880 min | ^[39]^ |
| D−Cu−Co_3_O_4_ | 0.5 M Na_2_SO_4_ and 1000 ppm NaNO_3_ | −0.4 V (versus RHE) | 96.4% | 1574.84  µg·h^−1^·mg_cat_^−1^ | 16 h | ^[40]^ |

**Table S8** Nitrate reaction kinetics of various catalysts.

| Catalysts | Electrolytes | Potential at -10mA cm^-2^(V vs. RHE) | Reaction constant(min^-1^) at FE Peak (10^-4^) |
| --- | --- | --- | --- |
| Cu-NC | 0.5M Na_2_SO_4_ + 0.1M NaNO_3_ | -0.66 | K_1_=2.05 |
|  | 0.5M Na_2_SO_4_ + 0.1M NaNO_2_ | -0.475 | K_2_=1.01 |
| Fe-NC | 0.5M Na_2_SO_4_ + 0.1M NaNO_3_ | -0.675 | K_1_=0.81 |
|  | 0.5M Na_2_SO_4_ + 0.1M NaNO_2_ | -0.47 | K_2_=2.08 |
| CuFe_x_-NC | 0.5M Na_2_SO_4_ + 0.1M NaNO_3_ | -0.447 | K_1_=2.60 |
|  | 0.5M Na_2_SO_4_ + 0.1M NaNO_2_ | -0.319 | K_2_=2.80 |

**Table S9** Adsorption energy of NO_3_^─^ and NO_2_^─^ at different metal sites.

| Intermediate | Cu | Fe_1_ | Fe_2_ | Fe_3_ |
| --- | --- | --- | --- | --- |
| NO_3_^─^ | -0.97184 | -0.69983 | -0.26454 | -0.24527 |
| NO_2_^─^ | -0.77184 | -0.97346 | -0.81028 | -0.78106 |

**References**

[1] B. Ravel, M. Newville, *J. Synchrotron Radiat.* 2005, *12*, 537.

[2] H. Funke, A. C. Scheinost, M. Chukalina, *Phys. Rev. B* 2005, *71*, 094110.

[3] H. Funke, M. Chukalina, A. C. Scheinost, *J. Synchrotron Radiat.* 2007, *14*, 426.

[4] S. Grenier, Y. Joly, *J. Phys.: Conf. Ser.* 2014, *519*, 012001.

[5] Y. Joly, Y. Ramos, O. Bunău, in *Int. Tables Crystallogr.*, Vol. I, 2024, 2.11.

[6] G. Kresse, J. Hafner, *Phys. Rev. B* 1993, *47*, 558.

[7] G. Kresse, J. Hafner, *Phys. Rev. B* 1994, *49*, 14251.

[8] J. Perdew, K. Burke, M. J. P. r. l. Ernzerhof, *Phys. Rev. Lett.* 1996, *77 18*, 3865.

[9] G. Kresse, D. J. P. R. B. Joubert, *Phys. Rev. B* 1999, *59*, 1758.

[10] G. Henkelman, B. P. Uberuaga, H. Jónsson, *J. Chem. Phys.* 2000, *113*, 9901.

[11] S. Zhang, J. Wu, M. Zheng, X. Jin, Z. Shen, Z. Li, Y. Wang, Q. Wang, X. Wang, H. Wei, J. Zhang, P. Wang, S. Zhang, L. Yu, L. Dong, Q. Zhu, H. Zhang, J. Lu, *Nat. Commun.* 2023, *14*, 3634.

[12] H. Lin, J. Wei, Y. Guo, Y. Li, X. Lu, C. Zhou, S. Liu, Y.-y. Li, *Adv. Funct. Mater.* 2024, *34*, 2409696.

[13] H. Chen, C. Zhang, L. Sheng, M. Wang, W. Fu, S. Gao, Z. Zhang, S. Chen, R. Si, L. Wang, B. Yang, *J. Hazard. Mater.* 2022, *434*, 128892.

[14] J. Wei, Y. Li, H. Lin, X. Lu, C. Zhou, Y.-y. Li, *Environmental Science and Ecotechnology* 2024, *20*, 100383.

[15] J. Cheng, W. Sun, G. Dai, X. Yang, R. Xia, Y. Xu, X. Yang, W. Tu, *Fuel* 2023, *332*, 126106.

[16] M. Xu, Q. Xie, D. Duan, Y. Zhang, Y. Zhou, H. Zhou, X. Li, Y. Wang, P. Gao, W. Ye, *ChemSusChem* 2022, *15*, e202200231.

[17] Z. Gao, Y. Lai, Y. Tao, L. Xiao, L. Zhang, F. Luo, *ACS Central Science* 2021, *7*, 1066.

[18] Y.-T. Xu, M.-Y. Xie, H. Zhong, Y. Cao, *ACS Catalysis* 2022, *12*, 8698.

[19] J. Yang, H. Qi, A. Li, X. Liu, X. Yang, S. Zhang, Q. Zhao, Q. Jiang, Y. Su, L. Zhang, J.-F. Li, Z.-Q. Tian, W. Liu, A. Wang, T. Zhang, *J. Am. Chem. Soc.* 2022, *144*, 12062.

[20] G.-F. Chen, Y. Yuan, H. Jiang, S.-Y. Ren, L.-X. Ding, L. Ma, T. Wu, J. Lu, H. Wang, *Nature Energy* 2020, *5*, 605.

[21] X.-F. Cheng, J.-H. He, H.-Q. Ji, H.-Y. Zhang, Q. Cao, W.-J. Sun, C.-L. Yan, J.-M. Lu, *Adv. Mater.* 2022, *34*, 2205767.

[22] Y. Xue, Q. Yu, Q. Ma, Y. Chen, C. Zhang, W. Teng, J. Fan, W.-x. Zhang, *Environ. Sci. Technol.* 2022, *56*, 14797.

[23] Y. Wang, H. Yin, F. Dong, X. Zhao, Y. Qu, L. Wang, Y. Peng, D. Wang, W. Fang, J. Li, *Small* 2023, *19*, 2207695.

[24] X. Zhao, X. Jia, Y. He, H. Zhang, X. Zhou, H. Zhang, S. Zhang, Y. Dong, X. Hu, A. V. Kuklin, G. V. Baryshnikov, H. Ågren, G. Hu, *Applied Materials Today* 2021, *25*, 101206.

[25] X. Zhao, X. Li, H. Zhang, X. Chen, J. Xu, J. Yang, H. Zhang, G. Hu, *J. Hazard. Mater.* 2022, *424*, 127319.

[26] J. Wei, G. Ye, H. Lin, Z. Li, J. Zhou, Y.-y. Li, *J. Colloid Interface Sci.* 2024, *670*, 798.

[27] J. Zhong, H. Duan, M. Cai, Y. Zhu, Z. Wang, X. Li, Z. Zhang, W. Qu, K. Zhang, D. Han, D. Cheng, Y. Shen, M. Xie, E. Cortes, D. Zhang, *Angew. Chem. Int. Ed.* 2025, e202507956.

[28] Y. Wang, M. Sun, J. Zhou, Y. Xiong, Q. Zhang, C. Ye, X. Wang, P. Lu, T. Feng, F. Hao, F. Liu, J. Wang, Y. Ma, J. Yin, S. Chu, L. Gu, B. Huang, Z. Fan, *Proc. Natl. Acad. Sci. U. S. A.* 2023, *120*, e2306461120.

[29] W. Jang, D. Oh, J. Lee, J. Kim, J. E. Matthews, H. Kim, S.-W. Lee, S. Lee, Y. Xu, J. M. Yu, S. W. Hwang, T. F. Jaramillo, J.-W. Jang, S. Cho, *J. Am. Chem. Soc.* 2024, *146*, 27417.

[30] Z. Zhang, Y. Lv, Y. Gu, X. Zhou, B. Tian, A. Zhang, Z. Yang, S. Chen, J. Ma, M. Ding, J.-L. Zuo, *Angew. Chem. Int. Ed.* 2025, *64*, e202418272.

[31] Z. Zhang, M. Wang, H.-R. Xing, X. Zhou, L. Gao, S. Chen, Y. Chen, H. Xu, W. Li, S. Yuan, C.-H. Li, Z. Jin, J.-L. Zuo, *Angew. Chem. Int. Ed.* 2025, e202505580.

[32] K. Liu, Z. Sun, X. Peng, X. Liu, X. Zhang, B. Zhou, K. Yu, Z. Chen, Q. Zhou, F. Zhang, Y. Wang, X. Gao, W. Chen, P. Chen, *Nat. Commun.* 2025, *16*, 2167.

[33] W. Yang, Z. Chang, X. Yu, P. Wu, R. Shen, L. Wang, X. Cui, J. Shi, *Advanced Science* 2025, *12*, 2416386.

[34] B. Zhou, L. Yu, W. Zhang, X. Liu, H. Zhang, J. Cheng, Z. Chen, H. Zhang, M. Li, Y. Shi, F. Jia, Y. Huang, L. Zhang, Z. Ai, *Angew. Chem. Int. Ed.* 2024, *63*, e202406046.

[35] X. Li, S. Xia, S. Yang, X. Yang, S. Zheng, X. Xu, Y. Wang, Q. Xu, Z. Jiang, *Angew. Chem. Int. Ed.* 2025, e202507479.

[36] X. Zhang, X. Liu, Z.-F. Huang, L. Gan, S. Zhang, R. Jia, M. Ajmal, L. Pan, C. Shi, X. Zhang, G. Yang, J.-J. Zou, *Energy Environ. Sci.* 2024, *17*, 6717.

[37] C. Feng, K. Bo, J. Wan, H. Zhang, Y. Wang, *Angew. Chem. Int. Ed.* 2025, e202505211.

[38] J. Park, J. Theerthagiri, N. Yodsin, W. Limphirat, P. Junmon, M. Y. Choi, *Adv. Mater.* 2025, 2506137.

[39] F. Shen, S. He, X. Tang, Y. Liu, Y. Wang, Y. Yin, X. Lv, W. Fu, Y. Zou, G. Jiang, L. a. Hou, *Angew. Chem. Int. Ed.* 2025, *64*, e202423154.

[40] W. Gao, Z. Yan, S. Tian, J. Cui, B. Xie, Q. Jiao, C. Zhong, J. Liu, *Applied Catalysis B: Environment and Energy* 2025, *377*, 125495.
